# Food-based indices for the assessment of nutritive value and environmental impact of meals and diets: A systematic review

**DOI:** 10.1371/journal.pone.0346150

**Published:** 2026-04-01

**Authors:** Eva-Leanne Thomas, David Livingstone, Anne P. Nugent, Jayne V. Woodside, Leona Lindberg, Paul Brereton

**Affiliations:** 1 Institute for Global Food Security, School of Biological Sciences, Queen’s University Belfast, Belfast, United Kingdom; 2 Institute of Food and Health, School of Agriculture and Food Science, University College Dublin, Dublin, Ireland; 3 Centre for Public Health, School of Medicine, Dentistry and Biomedical Sciences, Queen’s University Belfast, Belfast, United Kingdom; Veracruzana University: Universidad Veracruzana, MEXICO

## Abstract

Food production and consumption impact both human and planetary health. Clearly conveying how meals and diets influence nutritional adequacy and environmental sustainability is essential for informed decision-making among consumers. This systematic review identifies and examines existing food-based indices that classify or rank meals and diets based on both nutritive value and environmental impact, that the authors termed Nutritive and Environmental Combined Indices (NECIs). Following PRISMA 2020 guidelines, six bibliographic databases were searched in August 2025 using four search concepts: nutrition, environment, index, and meal/diet. Studies assessing both nutritive value and environmental impact of meals or diets, published between January 2009 and August 2025, were included. Two independent reviewers screened studies and extracted data on NECI characteristics. The review protocol was registered with PROSPERO (CRD42024537149). Twenty-five NECIs, presenting 27 methodological approaches, were identified: six specific to meals, nine for diets, and ten offering scope to be applied to either. Nutritive and Environmental Combined Indices varied widely in scoring methods, with 13 different nutritional and six different environmental scoring approaches. Presentation formats also differed: 19 used a single integrated metric, four reported scores in parallel, and two used both. Additionally, 12 NECIs considered other dimensions of sustainability, primarily economic (n = 10) followed by socio-cultural (n = 6). Among integrated NECIs, variations were observed in dimension weighting, methodological approaches, and ranking criteria. A strength of this review is its focus on NECIs beyond individual food products, assessing their applicability to meals and diets. The review synthesises factors such as nutritional and environmental scoring methodologies, functional units, system boundaries, composite scoring techniques, weighting approaches, index scoring frameworks, and databases used. However, significant methodological variation among NECIs posed challenges for direct comparison. These findings provide a foundation for the development of standardised NECIs, supporting public health efforts to promote healthy and sustainable meal and diet choices.

## 1. Introduction

Food labelling is a widely used public health strategy designed to influence consumption practices by providing consumers with accurate and transparent product information. While nutrition labelling is a mandatory and regulated component of food labelling in the United Kingdom [1], often appearing as voluntary front-of-pack (FOP) labels, there is a growing interest in incorporating sustainability information, commonly seen in the form of eco-labelling. This shift reflects increasing awareness of the food system’s role in climate change, resource depletion, and biodiversity loss [2,3]. However, sustainability is a broad and evolving concept encompassing multiple dimensions, such as affordability and cultural acceptability [4], leading to inconsistencies in its application. This review moves beyond the broader concept of “sustainability” to specifically assess environmental impact, alongside nutritive value, as these are the most developed dimensions for public health and food system transformation [5]. Presenting both nutritional and environmental information, either in parallel or as a single metric, may help mitigate potential consumer misperceptions, such as the “health halo” effect that has been associated with eco-labelling [6], and improve knowledge regarding healthy and environmentally responsible food choices.

Methodological approaches combining nutritive value and environmental impacts of foods are now appearing within the literature [7,8]. One previous literature review identified eighty-one methodological frameworks that combine the assessment of the environmental impacts of the food supply chain with the nutritional content of food intakes [8]. However, that review included methodological approaches that only list the environmental and nutritional impact and do not allow for ranking, such as programming optimisation and statistical analysis. A subsequent systematic review by Bunge et al. (2020) identified ten sustainable-food profiling models that scored individual foods according to at least two environmental impacts, of which six additionally scored individual foods based on nutritional quality [7]. However, Bunge et al. (2020) focused only on models that scored individual food items leaving a gap in understanding approaches applicable to meals and diets. Therefore, it is important to identify approaches that assess and rank the nutritive value and environmental impact of meals and diets. Filling this knowledge gap could be an important public health tool to inform consumer choice in settings such as restaurants, while also underpinning public health policy, such as menu reformulation or marketing restrictions.

Other work has been done to assess how sustainable healthy diets are defined [9] and how dietary indicators can be applied to the environmental assessment of foods [10]. Harrison et al. (2022) reviewed how sustainable healthy diets have been defined in the literature since 2010, identifying an exponential growth of publications after 2017 [9]. However, their scoping review focused on dietary indicators rather than methodological approaches to combining indicators of nutritive value and environmental impact.

More recently, Reguant-Closa et al. (2024) reviewed studies that applied nutritional health and environmental (NHE) assessments of foods, but found that not all included papers considered both dimensions simultaneously [10]. They emphasised the growing recognition of the need for an integrated or combined approach, with recommendations to ensure methodological transparency, comparability across indices, and where possible, integrated assessment to capture trade-offs and synergies. These recommendations are directly aligned with the purpose of the present review, which focuses specifically on identifying indices that combine, or present simultaneously, nutritive value and environmental impact, while also outlining their underpinning methodology.

In addition to these methodological recommendations, Reguant-Closa et al. (2024) [10] proposed a group classification system for dietary and health indices. This system categorised indices into five groups (A–E) including: nutrient/food quantity-based indices, guideline-based indices, and diversity-based indices, to nutrient quality-based indices and health-based indices. The strength of this approach lies in its recognition that indices differ not only in structure but also in their underlying purpose, and therefore should not be judged or applied in the same way. Building on this concept, the present review also considers the purpose for which indices are developed, not only their methodological characteristics, but also their intended public health purpose.

The primary aims of this review are to investigate what food-based indices exist that assess nutritive value and environmental impact for the classification or ranking of meals or diets, and to better understand the methodology and key characteristics underpinning these indices. Therefore, for the purposes of this systematic review, these indices will be collectively referred to as Nutritive and Environmental Combined Indices (NECIs). When referring to an individual measure, the term Nutritive and Environmental Combined Index (NECI) is used. The secondary aim of this review is to outline the public health purpose of each identified index. By synthesising this information, this review intends to support food system stakeholders and public health actors in the future development, or implementation of, a standardised index to promote healthy and environmentally friendly meal and diet choices for consumers.

## 2. Methods

### 2.1. Search strategy and selection criteria

This systematic review followed the Preferred Reporting Items for Systematic Reviews and Meta-Analyses (PRISMA) guidelines [11]. The protocol was registered in PROSPERO (CRD42024537149) and published as a study protocol [12]. The first search was undertaken on 12^th^ April 2024 and updated using steps outlined by Bramer and Bain [13] to capture studies published up to 21^st^ August 2025. Searches were conducted by the primary reviewer across six bibliographic databases (CAB Abstracts, EMBASE, FSTA, MEDLINE, Web of Science Core Collection (Science Citation Index & Social Science Citation Index) and Scopus) with the search terms (“nutrition” OR “nutritive value” OR “nutrient profiling”) AND (“environmental impact” OR “sustainability”) AND (“index” OR “model” OR “score” OR “tool”) AND (“meal” OR “diet”) AND NOT (“animals”). Full search details are available in the Supplementary Material (Supplemental Table 1 in S1 File). No language restrictions were applied. Where potentially eligible studies were published in languages other than English, full texts were translated using the document translation function of Google Translate to enable screening, and where appropriate, data extraction, and eligibility assessment. Translated manuscripts were checked for sufficient and coherent methodological detail relevant to data extraction. Searches were limited to original articles published between January 2009 and August 2025. Review articles were excluded, but relevant reference lists were screened for additional publications.

**Fig 1:**
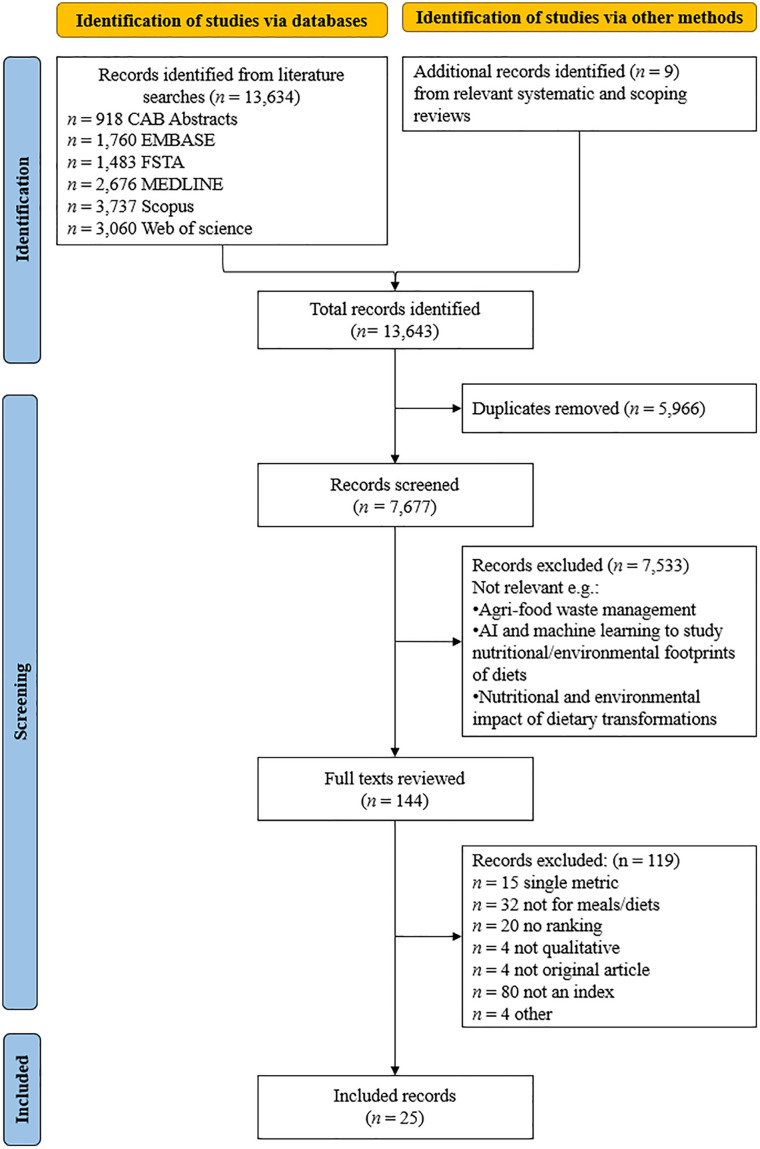
PRISMA 2020 flow diagram for the systematic review of studies reporting on food-based indices classifying or ranking meals or diets in terms of nutritive value and environmental impact and their intended public health purpose. From: [11].

Given the heterogeneous terminology and inconsistent reporting practices in this field, the search strategy was intentionally broad, as established through extensive search string scoping and piloting, to ensure capture of all key studies [12]. Consequently, eligibility criteria relating to index integration and analytical level (meal or diet) were applied conservatively at the title and abstract stage and assessed in detail at full-text screening to avoid premature exclusion of relevant studies. We included approaches assessing both nutritive value and environmental impact for meals or diets, excluding those focused solely on one aspect or on individual foods. Broader sustainability dimensions, such as economic and socio-cultural factors, were additionally considered, and approaches with any public health purpose, including, and beyond, food labelling, were included for a comprehensive review of published indices. Inclusion and exclusion criteria are detailed in Fig 1. and Supplemental Table 3 in S1 File.

A two-stage screening process was conducted independently by two blinded authors (ELT and DL), as outlined by Thomas et al. (2024) [12]. Discrepancies were resolved by a third reviewer (PB). A list of studies excluded at full-text screening, with reasons, is available in Supplemental Table 4 in S1 File.

### 2.2. Data extraction

To ensure consistency, the primary author (ELT) extracted data from all included studies, with the second author (DL) extracting every fifth article. Key study characteristics, methodological details of index creation, study aims, and public health purposes were collected, as detailed in the study protocol [12]. A comprehensive overview of extracted data is available in Supplemental Tables 5-8 in S1 File. All extracted data were cross-checked by ELT and DL.

### 2.3. Quality assessment

A replicability assessment was used as a quality metric, as it is more appropriate than traditional risk-of-bias assessments for this type of review [7]. Replicable, in this review, refers to the degree to which the methodology of an NECI is transparently described and can be independently reproduced. A replicable NECI provides clear details on how nutritional and environmental scores are calculated, including equations, weighting, system boundaries, reference amounts, and data sources. Details of this assessment are provided in Supplemental Table 9 in S1 File. The assessment was conducted independently by ELT and DL, with discrepancies resolved through discussion.

## 3. Results

A total of 13,643 records were retrieved, including 13,634 from six bibliographic databases and nine additional records from reference lists of relevant reviews (Fig 1). After removing duplicates, 7,677 records remained for title and abstract screening, where 5,966 were excluded. Full-text screening was conducted on 144 publications, with 119 excluded (Supplemental Table 3 in S1 File). In total, 25 studies met the inclusion criteria for analysis, 19 presented a single index [14–38], while two [30,34] provided one index with two scoring methods – separate scores and a combined score – resulting in 27 methodological approaches. Six indices were designed for meals [19,20,24,28,31,35], nine for diets [16–18,21,26,27,30,32,36], and ten could assess either [14,15,22,23,25,29,33,34,37,38] (Table 1). Sustainability dimensions beyond nutrition and environmental impact were incorporated in 12 indices (Table 1). These additional dimensions varied across indices and were not mutually exclusive. The most common additional dimension was economic sustainability (n = 10) [17–19,21,26,27,30,32,35,36], followed-by socio-cultural considerations (n = 6) [17,21,26,27,30,32]. One index additionally considered food production and management [31], and one included human health [23] as an additional sustainability dimension.

**Table 1:** The application, sustainability dimensions, and score type of food-based indices for the assessment of nutritive value and environmental impact of meals and diets.

		Index application			Index dimensions		Index score type	
		Meals	Diets	Scope for meals or diets	Nutritive and environmental	Nutritive, environmental and others*	Combined metric	Individual scores
**Publication citation**	**Index name**							
**Aidoo et al. (2023)** **[14]**	**Environmental Impact Weighted Daily Value score (EIWDVs) model**			✓	✓		✓	
**Bach et al. (2023)** **[15]**	**Sustainability Score**			✓		✓	✓	
**Batlle-Bayer et al. (2019)** **[16]**	**Index not clearly named**		✓		✓		✓	
**Batlle-Bayer et al. (2020)** **[17]**	**Index not clearly named**		✓			✓	✓	
**Cooreman-Algoed et al. (2020)** **[18]**	**Index not clearly named**		✓		✓			✓
**Costa da Silva et al. (2023)** **[19]**	**Healthy and sustainable preparation index (HSPI)**	✓				✓	✓	
**Dourmad et al. (2019)** **[20]**	**Index not clearly named**	✓				✓		✓
**Fresán et al. (2020)** **[21]**	**[new] sustainable diet index (SDI)**		✓			✓	✓	
**Goss and Sharewood (2024) [22]**	**Performance-weighted environmental sustainability (PwES)**			✓	✓		✓	
**Guido et al. (2020)** **[23]**	**The Food-Triad**			✓		✓	✓	
**Haupt et al. (2016)** **[24]**	**Eco-nutritional-efficiency (ENE)**	✓			✓		✓	
**Hooker et al. (2024)** **[25]**	**Sustainability Index**			✓		✓	✓	
**Kong et al. (2025) [26]**	**Food consumption sustainability index**		✓			✓	✓	
**Li et al. (2023)** **[27]**	**Comprehensive assessment index (CAI)**		✓			✓	✓	
**Lukas et al. (2016)** **[28]**	**Nutritional Footprint**	✓			✓		✓	
**Meier et al. (2024) [29]**	**Planet Health Conformity Index (PHC)**			✓	✓		✓	
**Rӧӧs et al. (2015)** **[30]**	**‘Method 1’** ^†^		✓		✓			✓
	**‘Method 2’** ^†^		✓		✓		✓	
**Schaubroeck et al. (2018)** **[31]**	**Index not clearly named**	✓				✓		✓
**Seconda et al. (2019)** **[32]**	**Sustainable diet index (SDI)**		✓			✓	✓	
**Sonesson et al. (2019)** **[33]**	**Dietary dependent nutrient quality index (NQI)**			✓	✓		✓	
**Strid et al. (2021)** **[34]**	**Parallel climate-nutrient score**			✓	✓			✓
	**Integrated climate-nutrient score**			✓	✓		✓	
**Takacs et al. (2025)** **[35]**	**Index not clearly named**	✓				✓	✓	
**Trijsburg et al. (2021)** **[36]**	**World Index for Sustainability and Health (WISH)**		✓		✓		✓	
**van Dooren et al. (2014)** **[37]**	**Index not clearly named**			✓	✓			✓
**Werner, Flysjo & Tholstrup. (2014)** **[38]**	**The Nutrient Density of Climate Impact (NDCI) index**			✓	✓		✓	

Note: Indices are listed alphabetically according to the first author of the cited publication.

*Other dimensions of sustainability include economic, socio-cultural, production and management of food and human health.

^†^Index not clearly named, however, author distinguishes between the two presentation methods of the index by defining each method as either ‘Method 1’ or ‘Method 2’.

### 3.1. Key characteristics of NECIs

Of the 25 indices, 15 were developed by a single institution [14,16–20,22–26,30,31,33,35]. while 10 resulted from institutional collaborations, including but not limited to companies, consultancy firms and non-governmental organisations [15,21,27–29,32,34,36–38]. Across all indices universities were the most frequently involved institutions (n = 23) [14–19,21–32,34–38], followed by research institutes (n = 6) [21,27,28,32–34] (Supplemental Table 5 in S1 File). Most indices were published from 2019 onwards (n = 19) [14–23,25–27,29,32–36] (Table 1). Of the 25 indices, 24 were intended for the adult population [14–21,23–38], while one index targeted the general population, implicitly including children – the performance-weighted environmental sustainability (PwES) index [22]. Four indices were specifically developed for university populations [18,19,21,31]. Most indices were developed for European populations (n = 17) [16–18,20–22,24,28–35,37,38], while others were created for Brazilian (n = 2) [19,23], American (n = 2) [14,25], Chinese (n = 2) [26,27] and Vietnamese (n = 1) [36] adults. The Sustainability Score was wider in scope, having been created for high-income nations more broadly rather than for a specific country or geographic region [15].

### 3.2. Methodological development of NECIs

Among the 25 NECIs reviewed, 13 distinct nutritional scoring methods categories and six distinct environmental scoring method categories were identified, with several NECIs employing the same methodological approaches (Table 2, Supplemental Table 6, 7 in S1 File).

**Table 2:** Overview of nutritional scoring methods, environmental scoring methods, functional units, validation, nutritional indicators, and system boundaries in food-based indices for the assessment of nutritive value and environmental impact of meals and diets.

	Nutritional Scoring Method					Environmental Scoring Method					
Index Name*	Name	Functional unit		Nutritional indicators included		Name	Functional unit		System boundary		
		Mass	Energy	Nutrients	Food groups		Amount of food^†^	Time span^‡^	Cradle-to-plate	Cradle-to-gate	Other^¶^
**Environmental Impact Weighted Daily Value score (EIWDVs) model** **[14]**	Weighted Daily Value Score (WDVS).	§	§	✓		Life Cycle Assessment (LCA)	✓			✓	
**Sustainability Score** **[15]**	SAIN:LIM	✓		✓		LCA	✓		§	§	§
**Batlle-Bayer et al. (2019)** **[16]**	Nutritional Score (NS) -based on Nutrient Rich Diet Index (NRD9.3)	✓		✓		LCA		✓	✓		
**Batlle-Bayer et al. (2020)** **[17]**	Nutritional Quality assessed via Nutrient Rich Diet Index (NRD9.3)	✓		✓		LCA		✓			✓
**Cooreman-Algoed et al. (2020)** **[18]**	Weighted Nutrient Density Score (WNDS) & nutritional thresholds.	✓	✓	✓		LCA	✓		✓		
**Healthy and sustainable preparation index (HSPI)** **[19]**	Energy Density (ED) & Nutritional Density (ND)	✓	✓	✓		Water, carbon and ecological footprint	✓		§	§	§
**Dourmad et al. (2019)** **[20]**	Not clearly stated	✓		✓		LCA	✓		✓		
**[new] sustainable diet index (SDI)** **[21]**	Nutritional Quality Index	§	§		✓	Environmental Impact Index (Fresan 2017)	✓			✓	
**Performance-weighted environmental sustainability (PwES)** **[22]**	Not clearly stated	✓		✓	✓	Share of Safe Operating Space	✓	✓			✓
**The Food-Triad** **[23]**	Nutrient Percentage	✓		✓		Environmental Dimension	§	§	✓		
**Eco-nutritional-efficiency (ENE)** **[24]**	Extent of Compliance Indicator (ECI)		✓	✓		LCA	✓		✓		
**Sustainability index** **[25]**	Nutrient Rich Foods Index 9.3 (NRF9.3)		✓	✓		Not clearly stated	✓				✓
**Food consumption sustainability index** **[26]**	Nutritional Quality	§	§		✓	Ecological Effect	✓		✓		
**Comprehensive assessment index (CAI)** **[27]**	Diet Balance Index_16 (DBI_16)	✓			✓	Environmental footprint	✓				✓
**Nutritional Footprint** **[28]**	Health Indicator (nutritional footprint health)	✓		✓		Environmental Indicators (nutritional footprint environment)	✓	✓	§	§	§
**Planet Health Conformity Index (PHC) [29]**	Not clearly stated – based on nutriRECIPE	✓		✓		Not clearly stated	✓				✓
**Rӧӧs et al. (2015)** **[30]**	Method One: Nutrient Density Score (NDS)	§	§	✓		LCA^**§**^	✓		✓		
**Rӧӧs et al. (2015)** **[30]**	Method Two: Nutrient-Rich Diet Score 9.3 (NRD 9.3), NRD 11.4-Riksmaten & NRD-LCHF	§	§	✓		LCA	✓		✓		
**Schaubroeck et al. (2018)** **[31]**	Not clearly stated		✓	✓		LCA	✓				✓
**Sustainable diet index (SDI)** **[32]**	PANDiet index & energy	✓	✓	✓		LCA		✓	✓		
**Dietary dependent nutrient quality index (NQI)** **[33]**	Nutrient Quality Index (NQI) developed from Nutrient Rich Foods Index 9.3 (NRF 9.3)	✓	✓	✓		LCA	✓		§	§	§
**Parallel climate-nutrient score** **[34]**	Nutrient Rich Foods Index 11.3 (NRF11.3)	✓	✓	✓		Climate Impact -based on LCA	✓				✓
**integrated climate-nutrient score** **[34]**	Nutrient Rich Foods Index 11.3 (NRF11.3)	✓	✓	✓		Climate Impact – based on LCA	✓				✓
**Takacs et al. (2025)** **[35]**	Nutrient Rich Food Index (NRF 9.3 and 17.3)	✓		✓		LCA	✓		✓		
**World Index for Sustainability and Health (WISH)** **[36]**	EAT-Lancet Report Recommendations	✓			✓	LCA	§	§	§	§	§
**van Dooren et al. (2014)** **[37]**	Health Score	✓	✓	✓		Sustainability Score (based on LCA)		✓		✓	
**The Nutrient Density of Climate Impact (NDCI) index** **[38]**	Nutritional value & nutrient density	✓		✓		Carbon Footprint	✓		✓		

Note: Indices are listed alphabetically according to the first author of the cited publication.

* Publication citation used where no index name is available.

^†^Functional units of the environmental indicator based on amount of food including, per serving weight, per unit of food, per kg or L, per hot meal served, per capita, per 100kcal and per 100g.

^‡^Functional units of the environmental indicator based on time span including, per year and per day.

^¶^Other system boundaries include cradle-to-grave, cradle-to-shelf/canteen, cradle-to-farm/plant gate, cradle-to-consumption (full details of system boundaries used for the environmental dimension of each NECI can be found in the supplementary material (Supplemental Table 7 in S1 File).

^§^Information not clearly given in article.

#### 3.2.1. Nutritional scoring methods of NECIs.

Of the 27 methodological approaches used, the most common nutritional scoring method was a variation of, or based on, the Nutrient Rich Diet (NRD) score (n = 8 of 27 methodological approaches) [16,17,25,30,33–35], including NRD 9.3 [16,17,25,30], Nutrient Rich Foods index 9.3 (NRF9.3) [33,35], NRF 11.3 [30], NRF 10.4 [30], and NRF 17.3 [35]. Most methodological approaches (n = 27) calculated nutrition scores based on mass (n = 12) [15–17,20,22,23,27–29,35,36,38], while some used energy (n = 3) [24,25,31], and some used both (n = 7) [18,19,32–34,37]. Four studies (five methodological approaches) did not specify this information [14,21,26,30]. Nutritional scoring was primarily based on individual nutrients (n = 22 methodological approaches) [14–20,23–26,28–35,37,38], while four approaches assessed nutrition via food groups [21,27,36] and one used both [22]. Further details on nutrients, food groups, and scoring methods are presented in Supplemental Table 6 in S1 File.

#### 3.2.2. Environmental scoring methods of NECIs.

The most common environmental assessment methodological approach was life cycle assessment (LCA) (n = 17 of 27 methodological approaches) [14,16–18,22,24,26,30–37] (Table 2). Most NECIs calculated environmental scores based on food quantity (n = 19 of 27 methodologies) [14,15,18–21,24–27,29–31,33–35,38], for example per kg or per 100g, per serving weight or per meal. Other NECIs environmental scoring approaches were based on time span (n = 4 of 27 methodologies) [16,17,32,37], for example per year or per day, two NECIs used both functional units [22,28], for example per kg per year, and two did not specify [23,36] (Table 2). System boundaries varied, including cradle-to-plate (n = 11 of 27 methodologies) [16,18,20,23,24,26,30,32,35,38] cradle-to-gate (n = 3) [14,21,37] cradle-to-grave (n = 2) [17,27], cradle-to-shelf or canteen (n = 2) [25,31], cradle-to-farm gate/plant gate (n = 2) [29,34], cradle-to-consumption (n = 1) [22]. Five studies did not specify system boundaries [19,20,28,33,36]. Further details on environmental scoring method used, including environmental impact indicators, system boundaries and scoring methods are detailed in Supplemental Table 7 in S1 File.

#### 3.2.3. Final scoring of NECIs for the assessment of nutritive value and environmental impact of meals and diets.

Among the 27 methodological approaches used to develop NECIs, the most common was the composite indicator approach, where dimensions were standardised or normalised before aggregation (n = 9 of 27 methodological approaches) [15,21,25–28,32,35,36]. The next most common approach divided one dimension by the other (n = 5), with two indices using nutrition as the numerator (healthy and sustainable preparation index (HSPI) and The Nutrient Density of Climate Impact (NDCI) index) [19,38] and three using the environmental dimension as the numerator (Environmental Impact Weighted Daily Value score (EIWDVs), the integrated climate-nutrient score and the Planet Health Conformity (PHC) index) as numerator [14,29,34]. Other approaches included using nutrition as a functional unit for LCA (n = 3 of 27 methodological approaches) [16,17,33], environmental impact per nutrient density score [30], data envelopment analysis [24], assessing nutritional units in proportion to food supply share of safe operating spaces [26], and calculating the area between plotted dimensions [23] (Table 3). Six of the 25 indices presented dimensions in parallel rather than combining them [20,25,30,31,34,37].

**Table 3:** Overview of criterion and scoring/ranking of food-based indices for the assessment of nutritive value and environmental impact of meals and diets.

	Index creation							Index scoring/ ranking	
	Methodological approach				Weighting			Relative	Absolute
Index Name*	Composite Indicator	Division of one dimension by another	Functional unit of LCA^†^	Other‡	Equal	Non-equal	Weighting N/A^¶^		
**Environmental Impact Weighted Daily Value score (EIWDVs) model** **[14]**		✓			§	§	§		✓
**Sustainability Score** **[15]**	✓					✓		✓	
**Batlle-Bayer et al. (2019)** **[16]**			✓		§	§	§		✓
**Batlle-Bayer et al. (2020)** **[17]**			✓		§	§	§		✓
**Cooreman-Algoed et al. (2020)** **[18]**	§	§	§	§			✓		✓
**Healthy and sustainable preparation index (HSPI)** **[19]**		✓			✓				✓
**Dourmad et al. (2019)** **[20]**				✓			✓		✓
**[new] sustainable diet index (SDI)** **[21]**	✓				✓			✓	
**Performance-weighted environmental sustainability (PwES)** **[22]**				✓			✓		✓
**The Food-Triad** **[23]**				✓	✓				✓
**Eco-nutritional-efficiency (ENE)** **[24]**				✓			✓		✓
**Sustainability index** **[25]**	✓				✓			✓	
**Food consumption sustainability index [26]**	✓					✓			✓
**Comprehensive assessment index (CAI)** **[27]**	✓					✓			✓
**Nutritional Footprint** **[28]**	✓				✓				✓
**Planet Health Conformity Index (PHC)** **[29]**				✓	✓				✓
**Rӧӧs et al. (2015)** **[30]**				✓			✓	✓	
**Rӧӧs et al. (2015)** **[30]**				✓	§	§	§	✓	
**Schaubroeck et al. (2018)** **[31]**				✓			✓		✓
**Sustainable diet index (SDI)** **[32]**	✓				✓			✓	
**Dietary dependent nutrient quality index (NQI)** **[33]**			✓		§	§	§		✓
**Parallel climate-nutrient score** **[34]**		✓					✓	✓	
**Integrated climate-nutrient score** **[34]**		✓			✓			✓	
**Takacs et al. (2025) [35]**	✓				✓			✓	
**World Index for Sustainability and Health (WISH)** **[36]**	✓				✓				✓
**van Dooren et al. (2014)** **[37]**				✓			✓	✓	
**The Nutrient Density of Climate Impact (NDCI) index** **[38]**		✓			§	§	§		✓

Note: Indices are listed alphabetically according to the first author of the cited publication.

* Publication citation used where no index name is available.

† Life Cycle Assessment.

‡Other methodological approaches included calculating the environmental impact per nutrient density score, data envelopment analysis and calculating the area between plotted values, or nutritional and environmental dimensions are presented in parallel.

¶ Weighting is not applicable to NECIs that present the dimensions in parallel (multi-component NECIs) or NECIs that avoid subjective weighting, for example by data envelopment analysis.

§ Information not clearly given in article.

Of the 21 single-metric indices, ten applied equal weighting to dimensions [19,21,23,25,28,29,32,34–36]. Three indices used unequal weighting, The Sustainability Score (0.66: 0.16: 0.16, environment: nutrition, affordability) and the Food Consumption Sustainability Index both prioritised the environmental dimension (0.32: 0.24: 0.23: 0.20, environment: social influence: nutrition: economic benefit) [15,26], while the comprehensive assessment index (CAI) favoured nutrition (0.3: 0.26: 0.25: 0.19, nutrition, environment, economy, socio-culture, respectively) [27]. The Eco-nutritional-efficiency (ENE), avoided subjective weighting [24], the performance-weighted environmental sustainability (PwES) weighted environmental impact by their nutritional content [22] and six indices did not specify weighting methods [14,16,17,30,33,38] (Table 3).

Scoring was more often absolute (n = 17 of 25 indices) [14,16–20,22–24,26–29,31,33,36,38] than relative (n = 8 of 25 indices) [15,21,25,30,32,34,35,37] (Table 3). Relative indices compare the performance of meals or diets in relation to one another by ranking or scoring by comparison within the dataset. Absolute indices are anchored to an external benchmark, threshold or normative standard to express how, or if, a meal or diet, meets, or exceeds, predetermined targets, limits or boundaries.

One index did not clearly report the data sources used for either dimension [23], while Schaubroeck et al. did not specify the source for the nutritive dimension [31], and Dourmad et al. lacked this information for the environmental dimension [20]. All other indices retrieved data from secondary sources. The most common data source for the nutritive dimension was nutrient composition databases (n = 13) [14,16–18,21,22,24,25,29,30,33,34,38]. For the environmental dimension, the most frequent data source was the primary literature (n = 14) [14–17,19,21,22,24–27,35,36,38].

Of the 25 indices, 21 were deemed replicable [14–18, 21–38], one possibly replicable [19], and one not replicable [20]. Validation details of each NECI are detailed in supplemental Table 8 in S1 File.

### 3.3. Public health purpose of NECIs

Key public health aims of the identified NECIs included policy development [14,17,18,23,26,27,30–32], decision-making support for stakeholders beyond policymakers [14,24,31,33–35,38], research and innovation [16,36], consumer focus and knowledge development [20,22,24,25,28,29,35] and menu or dietary guideline development [15,19,21,30,34,37]. Several indices aimed to inform public health and sustainability policies, including food system sustainability [14,27,30,38] and university canteen management [18,31]. Others supported stakeholders by aiding menu development [19], sustainability planning [24,33], or providing tools to promote sustainable consumption [20,32]. Two indices specifically targeted future food system research [16,36]. Guiding consumers toward healthier and more sustainable choices was a key objective for multiple indices [17,20,23,25,28,37,38], while some were designed to inform and refine dietary guidelines [15,21,34,37].

## 4. Discussion

This review identified food-based indices that assess both the nutritive value and environmental impact of meals and diets, analysed their key characteristics, and explored their public health purpose. Assessing indices beyond individual foods is essential, as people consume composite meals rather than individual ingredients, which collectively shape dietary patterns. Additionally, examining indices with purposes beyond food labelling is important, given that front-of-pack nutrition labelling has been shown to improve consumer understanding and intention to purchase in experimental settings, but demonstrates only modest and inconsistent effects on real-world purchasing behaviour [39]. The review examined 25 indices (27 methodological approaches), highlighting their key characteristics, including their creation methods and intended public health purpose. In doing so, the review identified significant inconsistencies in NECIs development, driven by diverse methodological approaches, which pose challenges to widespread adoption. These findings support prior research [7–10] calling for harmonised criteria to enable consistent comparisons and support the development of a standardised index to guide public health actors in promoting healthy and environmentally responsible meal and diet choices.

In 2021, Bunge et al. identified ten sustainable food profiling models that assessed the nutritive value and environmental impact of individual foods [7]. While the present review extends beyond individual food items to encompass meals and dietary patterns, the earlier work in comparison to this work highlights the expanding body of literature examining dual outcomes of nutritive quality and environmental impact across various levels of the food system. The growth of research in this area shows both the importance of the field and the inherent challenge of capturing all relevant publications within a defined period. Though it was necessary to establish a clear temporal cut-off to ensure the feasibility and completeness of the search process, other publications within this field continue to be published [40–42].

One such study described the development of the Sus-Health Index (published September 2025), a combined measure for describing environmental impact and nutritive value of foods and meals [42]. This index integrates the UK Ofcom nutrient profiling model, utilising the Nutri-Score five-scale score, with The European Food Environmental Footprint Single Index, utilising the Enviroscore five-scale score, using a composite indicator approach to jointly assess food and meal performance. Designed to support consumer food choices through labelling applications, the Sus-Health Index exemplifies ongoing efforts to create integrated tools for sustainability communication. Although the Sus-Health Index shares conceptual similarities with several indices included in this review, it further highlights the methodological diversity, and variation in intended use, that can be seen across such tools.

While 19 indices used a single combined score, this approach risks oversimplification, potentially obscuring trade-offs and reducing transparency. Therefore, indices presenting nutrition and environmental impact as parallel scores were also included. Six studies [18,20,30,31,34,37] presented multi-component indices, five of which aimed to either inform policy [18,30,31] or dietary guideline development [34,37]. Multi-component indices may offer greater transparency, potentially making them more suitable for policymaking, whereas single-metric indices could be more effective for consumer-focused applications like food labelling or menu development. Alternatively, Takacs et al., (2025), suggested the use of individual and integrated assessments in parallel, to provide a more nuanced understanding of the different sustainability dimensions of meals [35], as was done by two of the included indices in the review [30,34]. Regarding different dimensions of sustainability, economic and socio-cultural dimensions were also considered in some indices, reflecting growing awareness of affordability in food sustainability and the importance of cultural, social, and ethical factors [43]. Kong et al. (2025) reported synergistic effects between the dimensions included in the Food Consumption Sustainability Index (nutrition, environment, economic benefit and social influence) and implied that co-ordination of all dimensions of sustainability could help achieve sustainable food consumption and is therefore a future recommendation [26].

However, the inclusion of both single and multi-component indices, as well as those incorporating additional dimensions beyond nutrition and environment, added complexity to data extraction and overall interpretation. Striking a balance between including a broad range of indices whilst ensuring accurate categorisation and naming, proved challenging. To align with the review’s focus, a standardised naming approach was necessary, leading to the adoption of the term Nutritive and Environmental Combined Indices (NECIs). These indices were primarily developed by university research groups, often in collaboration with private research institutions and industry stakeholders, reflecting the need for multi-sectoral cooperation in food system transformation.

Nutritive and Environmental Combined Indices’ (NECIs) nutritional scores are primarily based on nutrients rather than food groups and comparing them is challenging due to variations in included indicators. For example, the healthy and sustainable preparation index (HSPI) [19] is micronutrient-based whereas, the Eco-nutritional-efficiency (ENE) [24], the Nutritional Footprint [28], and the indices by Cooreman-Algoed et al. (2020) [18] and by Schaubroeck et al. (2018) [31] focus on macronutrients or functional nutrients (fibre). The Nutrient Rich Foods index 9.3 (NRF9.3), along with its variations (NRF11.3, NRF10.4) and the Nutrient Rich Diet (NRD) score is the most commonly used method for assessing the nutritional dimensions of NECIs, supporting previous findings [8]. Its widespread use is likely due to applicability beyond individual foods [44], and its validation [45].

Environmental indicators also vary significantly across NECIs. The Environmental Impact Weighted Daily Value score (EIWDVs) [14] included 10 impact categories and The Food-Triad [23] included 22 indicators across seven groups. Contrastingly, some NECIs [16,18,24,33,34,38] considered only one impact category, typically, Greenhouse Gas (GHG) emissions (Global Warning Potential (GWP) or CO_2_e), the most commonly used metric across all NECIs. While GHG emissions correlate well with acidification and eutrophication [46], they do not always reflect broader impacts, such as blue water use [46]. This limitation is particularly relevant for aquatic animal source foods, which, despite lower GHG emissions, contribute significantly to biodiversity loss, habitat destruction, and water pollution [47]. Applying uniform scoring across all food categories may underestimate environmental impacts, especially in indices prioritising GHG emissions [5]. In contrast to previous work none of the included NECIs incorporated category specific scoring [7].

Life Cycle Assessment (LCA) was the most frequently used method for quantifying environmental impact, consistent with prior findings [7,8]. Several indices [14,15,17,20,31,32,35,36] assessed multiple environmental impact indicators, including: GHG emissions, eutrophication, acidification, land use and blue water footprint, providing a holistic view of a meal’s or diet’s environmental impact. However, methodological differences, lack of standardisation, and varying system boundaries complicate comparison across NECIs. Two recent papers describing indices included in this review reinforce these concerns, each highlighting limitations in the use of LCA, particularly nutritional LCA (nLCA), for assessing the environmental impacts of foods, meals, or diets [22,29]. Goss et al. (2025) emphasised that conventional LCA mid-point indicators, such as greenhouse gas emissions per calorie, may not accurately capture overall sustainability or enable consistent comparison across different impact categories [22]. Similarly, Meier et al. (2024) highlighted several methodological constraints, including the limited range of environmental indicators typically considered, restricted consideration of foods, nutrients, and health outcomes, and insufficient alignment with planetary boundaries [29]. Ensuring LCA remains an effective tool for NECIs requires standardisation and further research.

In all of the NECIs, nutritive and environmental impact values were derived from secondary data sources. Nutrition and Environmental Combined Indices (NECIs) nutritional assessments primarily relied on nutrition databases and food composition tables, for example the Food Composition and Nutrition Tables by Souci et al. (2008) [48] used in the Nutritional Footprint [28]. Conversely, environmental impact calculations relied more on the published literature, (e.g., Poore and Nemecek [3], used in the Sustainability Index [25], the PwES index [22], and The Sustainability Score [15]). This disparity reflects the greater maturity and standardisation of nutrition compared to environmental databases in relation to food [5]. Index implementation is reliant on the quality and availability of their underpinning data. High variability in nutrient composition across products already poses challenges for assessing food quality, and this variability is magnified when scaled up to meal or dietary level [29]. Environmental impact assessments lack comprehensive and standardised datasets, and limited access to high-quality primary data further weakens their reliability. Many supply chain actors struggle to quantify and share environmental impact data, leading to reliance on low-quality or aggregated sources. Consequently, an index can only be as robust as the data upon which it is built. Improving the quality, coverage, and accessibility of both environmental and nutritional datasets is therefore critical to advancing NECIs development and is essential to enable accurate, comparable, and meaningful assessments of food sustainability.

Beyond variations in scoring methods, there is no standardised approach to deriving a final NECI score, whether multi-component or combined. The most common method was the creation of a composite indicator, a validated approach for summarising complex, multidimensional issues to support decision-making [49]. Composite indicators, offering both continuous and discrete scores, are particularly useful for consumer guidance and policy development by simplifying complex information. This may explain their popularity, given that many NECIs aim to promote healthier and more environmentally sustainable meal/diet choices. However, challenges remain, including the integration of nutritional and environmental dimensions with differing cut-off points and the potential bias introduced by weighting these dimensions, which may introduce bias toward either nutrition or environmental impact.

There is no consensus on how to allocate weight across sustainability dimensions, leading most NECIs to apply equal weighting. The SDGs Wedding Cake model suggests prioritising environmental sustainability as the foundation of their model [50], which some NECIs reflect such as (i) The Sustainability Score [15] that prioritised environmental impact (0.66) over nutrition and affordability (0.16 each), and (ii) the Food Consumption Sustainability Index [26] that prioritised environmental impact (0.32) over social influence (0.24), nutrition (0.23) and economic benefit (0.20). Conversely, The CAI [27], placed heavier weighting on the nutrition dimension (0.3 vs 0.26, 0.25 and 0.19, for environmental, economic and socio-cultural dimensions, respectively). Similar challenges arise when one dimension is divided by another, as was done in the HSPI [19], The Nutrient Density of Climate Impact (NDCI) index [38], the EIWDVs [14], the integrated climate-nutrient index [34], and the Planet Health Conformity Index (PHCI) [29], raising concerns about methodology validation despite the simplicity and replicability of this approach. Another approach is integrating nutrition into the LCA functional unit [8], though this tends to prioritise environmental factors and does not offer a user-friendly outcome, making decision-making support difficult. Only one paper in the studies looked at applying different weighting approaches to their index after initially opting for equal weighting [35]. The analysis showed that varying the weights did not substantially alter the relative ranking of meals, as the most sustainable meals, those with higher nutritional quality, lower environmental impact, and lower cost, remained consistently plant-based, while meals with animal-based ingredients were consistently less sustainable (lower nutritional quality, higher environmental impact and higher cost). However, this analysis was limited to 13 variations of four meal types (chilli, lasagne, curry, and teriyaki), assessed in relation to one another. It remains uncertain whether weighting would have a greater influence if applied to absolute indices, rather than relative comparisons, or if assessed across a broader range of meals, or at the dietary level. Assessing the most appropriate weighting for NECIs is beyond the scope of this work and requires future exploration.

Hence, it is important to consider whether an index is relative or absolute. Relative ranking, preferred by consumers when looking at carbon emissions of individual food products [51], could be appropriate for NECIs aiming to identify the “best” and “worst” meals within a menu, as demonstrated by Takacs et al. (2025) [35]. However, it may mislead consumers, as the top-ranked option is not necessarily nutritionally adequate or environmentally sustainable. Absolute ranking, on the other hand, allows universal comparability and enables long-term monitoring, as shown by Trijsburg et al. (2021) [36]. Additionally, absolute scoring could enable cross comparison of different environmental impacts of different foods as is demonstrated in the PwES index where the climate change PwES of broccoli can be compared to the freshwater use PwES of apples [22].

A final important consideration is that indices are not neutral tools, but rather designed with specific objectives in mind. The public health rationale underpinning their development fundamentally shapes both their methodology and their eventual application. As our review shows, indices vary widely in the degree to which they align with different outlined public health priorities – whether that is guiding consumer choice, informing policy, underpinning future food-system research, or enabling dietary guideline development. This emphasises the need to critically examine not only how indices are constructed, but also the purpose they are intended to serve. Perhaps in addition to the group classification system for dietary and health indices outlined by Reguant-Closa et al. (2024) [10], a classification system based on intended public health purpose or application could be developed. By outlining their public health purpose, their suitability for different contexts can be better evaluated, avoid misapplication, and identify gaps where new or adapted indices may be required.

A rigorous and comprehensive literature search was conducted across multiple databases ensuring that all relevant studies were identified and included within the search time-frame. However, the lack of standardisation in NECI development, including differences in methodologies, scoring approaches, and weighting, made direct comparisons challenging. This inconsistency presents a significant barrier to their practical application by public health actors and food system stakeholders. To enhance usability and impact, future efforts should focus on developing standardised guidelines for NECI construction dependant on their intended use, ensuring transparency, consistency, and alignment with their intended public health and policy goals. No language restrictions were applied, ensuring a thorough literature search. While technical translation poses risks of altered meaning and is a potential limitation, including studies regardless of language ensured confidence that all relevant NECIs were captured. Though the focus was on indices assessing nutrition and environmental sustainability dimensions, those incorporating additional dimensions were included but not analysed in detail beyond their weighting. Further research should explore these broader aspects. Additionally, the term “NECI” may not fully reflect the broader scope of some indices but was used for consistency in this systematic review.

Future research should explore indices that assess multiple sustainability dimensions and evaluate their effectiveness in communication, implementation, and achieving public health goals. Additionally, the potential role of NECIs in food and meal reformulation should be investigated to support the development of more sustainable and nutritious options. Further research is also needed to examine the acceptance of NECIs among food system stakeholders and identify barriers to their adoption, as limited evidence exists on their practical integration into policy, industry, and consumer decision-making.

## 5. Conclusion

This systematic review is the first to identify and evaluate food-based indices that assess both the nutritive value and environmental impact of meals and diets. The considerable variation in NECIs methodologies highlights challenges in standardisation, limiting their comparability and widespread adoption. Despite this, NECIs have significant potential as public health tools for meal reformulation, marketing regulations, and dietary guideline development, ultimately aiming to improve the nutritive value and environmental impact of meals and diets. By providing clear and accessible information, NECIs can empower consumers to make more informed food choices, supporting shifts toward healthier and more environmentally responsible eating patterns. To maximise their impact, future research should prioritise standardisation while also exploring the practical adoption of NECIs, addressing barriers to implementation, and ensuring they effectively support healthier and more environmentally sustainable food choices.

## Supporting information

S1 File Supplemental Tables. (DOCX)

S2 File PRISMA 2020 Abstracts Checklist. (DOCX)

S3 File PRISMA 2020 Checklist. (DOCX)

## References

[1] Population Health Division, Department of Health and Social Care. Technical guidance on nutrition labelling London, United Kingdom. 2016.

[2] Crippa M, Solazzo E, Guizzardi D, Monforti-Ferrario F, Tubiello FN, Leip A. Food systems are responsible for a third of global anthropogenic GHG emissions. Nat Food. 2021;2(3):198–209. doi: 10.1038/s43016-021-00225-9 37117443

[3] Poore J, Nemecek T. Reducing food’s environmental impacts through producers and consumers. Science. 2018;360(6392):987–92. doi: 10.1126/science.aaq0216 29853680

[4] Ehgartner U. Discourses of the food retail industry: Changing understandings of ‘the consumer’ and strategies for sustainability. Sustain Produc Consump. 2018;16:154–61. doi: 10.1016/j.spc.2018.08.002

[5] Geyik Ö, Seferidi P, Barrett EM, Jones A, Pettigrew S, Wu JHY, et al. Learning from nutrient profile models to inform environmental profile models. Lancet Planet Health. 2024;8(12):e974–6. doi: 10.1016/S2542-5196(24)00269-9 39674201

[6] Wolfson JA, Musicus AA, Leung CW, Gearhardt AN, Falbe J. Effect of Climate Change Impact Menu Labels on Fast Food Ordering Choices Among US Adults. JAMA Netw Open. 2022;5(12):e2248320. doi: 10.1001/jamanetworkopen.2022.48320 PMC9857560 36574248

[7] Bunge AC, Wickramasinghe K, Renzella J, Clark M, Rayner M, Rippin H, et al. Sustainable food profiling models to inform the development of food labels that account for nutrition and the environment: a systematic review. Lancet Planet Health. 2021;5(11):e818–26. doi: 10.1016/S2542-5196(21)00231-X 34774122

[8] Grigoriadis V, Nugent A, Brereton P. Working towards a combined measure for describing environmental impact and nutritive value of foods: A review. Trends Food Sci Technol. 2021;112:298–311. doi: 10.1016/j.tifs.2021.03.047

[9] Harrison MR, Palma G, Buendia T, Bueno-Tarodo M, Quell D, Hachem F. A Scoping Review of Indicators for Sustainable Healthy Diets. Front Sustain Food Syst. 2022;5. doi: 10.3389/fsufs.2021.822263

[10] Reguant-Closa A, Pedolin D, Herrmann M, Nemecek T. Review of Diet Quality Indices that can be Applied to the Environmental Assessment of Foods and Diets. Curr Nutr Rep. 2024;13(2):351–62. doi: 10.1007/s13668-024-00540-0 38625631 PMC11133024

[11] Page MJ, McKenzie JE, Bossuyt PM, Boutron I, Hoffmann TC, Mulrow CD, et al. The PRISMA 2020 statement: an updated guideline for reporting systematic reviews. BMJ. 2021;n71. doi: 10.1136/bmj.n71 PMC8005924 33782057

[12] Thomas E-L, Livingstone D, Nugent AP, Woodside JV, Brereton P. Food-based indices for the assessment of nutritive value and environmental impact of meals and diets: A systematic review protocol. PLoS One. 2024;19(12):e0315894. doi: 10.1371/journal.pone.0315894 39705263 PMC11661603

[13] Bramer W, Bain P. Updating search strategies for systematic reviews using EndNote. J Med Libr Assoc. 2017;105(3):285–9. doi: 10.5195/jmla.2017.183 28670219 PMC5490709

[14] Aidoo R, Romana CK, Kwofie EM, Baum JI. An integrated environmental nutrition model for dietary sustainability assessment. J Clean Produc. 2023;399:136473. doi: 10.1016/j.jclepro.2023.136473

[15] Yacoub Bach L, Jana BE, Adaeze Egwatu CF, Orndorff CJ, Alanakrih R, Okoro J, et al. A sustainability analysis of environmental impact, nutritional quality, and price among six popular diets. Front Sustain Food Syst. 2023;7. doi: 10.3389/fsufs.2023.1021906

[16] Batlle-Bayer L, Bala A, Lemaire E, Albertí J, García-Herrero I, Aldaco R, et al. An energy- and nutrient-corrected functional unit to compare LCAs of diets. Sci Total Environ. 2019;671:175–9. doi: 10.1016/j.scitotenv.2019.03.332 30928747

[17] Batlle-Bayer L, Bala A, Albertí J, Xifré R, Aldaco R, Fullana-i-Palmer P. Food affordability and nutritional values within the functional unit of a food LCA. An application on regional diets in Spain. Resourc Conserv Recycl. 2020;160:104856. doi: 10.1016/j.resconrec.2020.104856

[18] Cooreman-Algoed M, Huysveld S, Lachat C, Dewulf J. How to integrate nutritional recommendations and environmental policy targets at the meal level: A university canteen example. Sustain Produc Consump. 2020;21:120–31. doi: 10.1016/j.spc.2019.10.004

[19] Da Silva TTC, Falco BB, De Castro IG, Zanon RB, Guerra JVV, Yaginuma KY, et al. Carbon, Water, Ecological Footprints, Energy and Nutritional Densities of Omnivore and Vegan Culinary Preparations. FNS. 2023;14(07):626–37. doi: 10.4236/fns.2023.147041

[20] Dourmad JY, van der Werf HMG, Mairesse G, Schmitt B, Chesneau G, Kerhoas N. Multidimensional evaluation and development of a tool for the improvement of sustainability of menus. Cahiers de Nutrition et de Diététique. 2019;54:223–9. doi: 10.1016/j.cnd.2019.03.002

[21] Fresán U, Martínez-González MA, Segovia-Siapco G, Sabaté J, Bes-Rastrollo M. A three-dimensional dietary index (nutritional quality, environment and price) and reduced mortality: The “Seguimiento Universidad de Navarra” cohort. Prev Med. 2020;137:106124. doi: 10.1016/j.ypmed.2020.106124 32437702

[22] Goss MA, Sherwood J. An absolute environmental sustainability assessment of food. Food Frontiers. 2024;5(3):855–66. doi: 10.1002/fft2.371

[23] de Almeida Sampaio Guido Y, Fonseca G, de Farias Soares A, da Silva ECN, Gonçalves Ostanik PA, Perobelli JE. Food-triad: An index for sustainable consumption. Sci Total Environ. 2020;740:140027. doi: 10.1016/j.scitotenv.2020.140027 32563875

[24] Haupt M, Clemente Polo G, García-Segovia P, Sanjuán Pellicer N. Approach to the integration of the carbon footprint and nutritional aspects for sustainable food consumption. Rev Española Nutric Comun. 2016;22(1):2–9. doi: 10.14642/RENC.2016.22.1.5124

[25] Hooker K, Sanjeevi N, Monsivais P. Identifying optimally sustainable foods: A four-dimensional analysis of sustainable foods in the American diet. Sustainability. 2024;16(2). doi: 10.3390/su16020551

[26] Kong F, Cui W, Bao S. Dynamic changes and sustainability assessment of food consumption footprint in megacities: A comparative analysis from four Chinese municipalities. Sustain Cities Soc. 2025;127:106433. doi: 10.1016/j.scs.2025.106433

[27] Li Y, Filimonau V, Wang L, Cheng S. A set of preliminary indicators for holistic sustainability assessment of household food consumption in rural and urban China. Resourc Conserv Recycl. 2023;188:106727. doi: 10.1016/j.resconrec.2022.106727

[28] Lukas M, Rohn H, Lettenmeier M, Liedtke C, Wiesen K. The nutritional footprint – integrated methodology using environmental and health indicators to indicate potential for absolute reduction of natural resource use in the field of food and nutrition. J Clean Produc. 2016;132:161–70. doi: 10.1016/j.jclepro.2015.02.070

[29] Meier T, Schade S, Forner F, Eberle U. Bridging Nutritional and Environmental Sustainability Within Planetary Boundaries in Food Life Cycle Assessments: SWOT Review and Development of the Planet Health Conformity Index. Sustainability. 2024;16(23):10658. doi: 10.3390/su162310658

[30] Röös E, Karlsson H, Witthöft C, Sundberg C. Evaluating the sustainability of diets–combining environmental and nutritional aspects. Environ Sci Policy. 2015;47:157–66. doi: 10.1016/j.envsci.2014.12.001

[31] Schaubroeck T, Ceuppens S, Luong AD, Benetto E, De Meester S, Lachat C, et al. A pragmatic framework to score and inform about the environmental sustainability and nutritional profile of canteen meals, a case study on a university canteen. J Clean Produc. 2018;187:672–86. doi: 10.1016/j.jclepro.2018.03.265

[32] Seconda L, Baudry J, Pointereau P, Lacour C, Langevin B, Hercberg S, et al. Development and validation of an individual sustainable diet index in the NutriNet-Santé study cohort. Br J Nutr. 2019;121(10):1166–77. doi: 10.1017/S0007114519000369 30973117

[33] Sonesson U, Davis J, Hallström E, Woodhouse A. Dietary-dependent nutrient quality indexes as a complementary functional unit in LCA: A feasible option? J Clean Produc. 2019;211:620–7. doi: 10.1016/j.jclepro.2018.11.171

[34] Strid A, Hallström E, Sonesson U, Sjons J, Winkvist A, Bianchi M. Sustainability Indicators for Foods Benefiting Climate and Health. Sustainability. 2021;13(7):3621. doi: 10.3390/su13073621

[35] Takacs B, Kalea AZ, Borrion A. Menu Dilemmas: An Integrated Assessment of the Nutritional Quality, Environmental Impact, and Cost of Vegan, Vegetarian, and Meat-Based Versions of Meals. Nutrients. 2025;17(9):1569. doi: 10.3390/nu17091569 40362878 PMC12073478

[36] Trijsburg L, Talsma EF, Crispim SP, Garrett J, Kennedy G, de Vries JHM, et al. Method for the Development of WISH, a Globally Applicable Index for Healthy Diets from Sustainable Food Systems. Nutrients. 2020;13(1):93. doi: 10.3390/nu13010093 33396659 PMC7824146

[37] van Dooren C, Marinussen M, Blonk H, Aiking H, Vellinga P. Exploring dietary guidelines based on ecological and nutritional values: A comparison of six dietary patterns. Food Policy. 2014;44:36–46. doi: 10.1016/j.foodpol.2013.11.002

[38] Werner LB, Flysjö A, Tholstrup T. Greenhouse gas emissions of realistic dietary choices in Denmark: the carbon footprint and nutritional value of dairy products. Food Nutr Res. 2014;58:10.3402/fnr.v58.20687. doi: 10.3402/fnr.v58.20687 24959114 PMC4053929

[39] Nohlen H, Bakogianni I, Grammatikaki E, Ciriolo E, Pantazi M, Diaz JA, et al. Front-of-pack nutrition labelling schemes: an update of the evidence. 2022 [cited 2023 Dec 12]. Available from: https://publications.jrc.ec.europa.eu/repository/handle/JRC130125

[40] Adiyan NN, Beyhan Y, Dayi T. Evaluation of the Carbon Footprint, Water Footprint, Nutrient Profiles and Cost of Sustainable Menus Planned With Digital Modeling. Food Sci Nutr. 2025;13(9):e70977. doi: 10.1002/fsn3.70977 40979572 PMC12445120

[41] Muzzioli L, Di Vincenzo O, Casado Mansilla D, Pintavalle M, Maddaloni L, Piciocchi C, et al. Developing an augmented nutrient profiling system in the perspective of healthy and sustainable diets. Int J Food Sci Nutr. 2025;76(7):701–8. doi: 10.1080/09637486.2025.2568676 41052876

[42] Grigoriadis V, Livingstone D, Thomas E-L, Brereton P, Woodside J, Nugent A, et al. Developing the Sus-Health Index: a combined measure for describing environmental impact and nutritive value of foods and meals. Philos Trans R Soc Lond B Biol Sci. 2025;380(1935):20240160. doi: 10.1098/rstb.2024.0160 40963361 PMC12444373

[43] van Bussel LM, Kuijsten A, Mars M, van ‘t Veer P. Consumers’ perceptions on food-related sustainability: A systematic review. J Clean Produc. 2022;341:130904. doi: 10.1016/j.jclepro.2022.130904

[44] Bullock SL, Miller HM, Ammerman AS, Viera AJ. Comparisons of Four Diet Quality Indexes to Define Single Meal Healthfulness. J Acad Nutr Diet. 2022;122(1):149–58. doi: 10.1016/j.jand.2021.06.010 34303634 PMC8688208

[45] Fulgoni VL 3rd, Keast DR, Drewnowski A. Development and validation of the nutrient-rich foods index: a tool to measure nutritional quality of foods. J Nutr. 2009;139(8):1549–54. doi: 10.3945/jn.108.101360 19549759

[46] Vellinga RE, van de Kamp M, Toxopeus IB, van Rossum CTM, de Valk E, Biesbroek S, et al. Greenhouse Gas Emissions and Blue Water Use of Dutch Diets and Its Association with Health. Sustainability. 2019;11(21):6027. doi: 10.3390/su11216027

[47] Gephart JA, Henriksson PJG, Parker RWR, Shepon A, Gorospe KD, Bergman K, et al. Environmental performance of blue foods. Nature. 2021;597(7876):360–5. doi: 10.1038/s41586-021-03889-2 34526707

[48] Souci SW, Fachmann W, Kraut H. Food composition and nutrition tables. 7ème edition ed. Stuttgart: MedPharm; 2008.

[49] Nardo M, Saisana M, Saltelli A, Tarantola S. Tools for composite indicators building. Brussels: European Comission, 2005 EUR 21682 EN Contract No.: JRC31473.

[50] Stockholm Resilience Centre. The SDGs wedding cake Stockholm, Sweden. 2016 [cited 2024 22 Aug]. Available from: https://www.stockholmresilience.org/research/research-news/2016-06-14-the-sdgs-wedding-cake.html

[51] Shapre A, Wheeler M. Reducing householders’ grocery carbon emissions: Carbon literacy and carbon label preferences. AMJ. 2013;21(4):240–9. doi: 10.1016/j.ausmj.2013.08.004

