# Supplementary material for: Food-based indices for the assessment of nutritive value and environmental impact of meals and diets: A systematic review
Source: PLoS One. 2026 Apr 1;21(4):e0346150. doi: 10.1371/journal.pone.0346150 (PMC13042827; doi:10.1371/journal.pone.0346150)
Supplement: S1 File — (DOCX) [file pone.0346150.s001.docx]

**Supplementary Material: Supplemental Tables**

Food-based indices assessing nutritive value and environmental impact of meals and diets: a systematic review.

Contents

[Supplemental Table 1: Search strategies on August 18 2025 to August 21 2025 2](#_Toc212732316)

[Table 1.1 Database: CAB Abstracts 2](#_Toc212732317)

[Table 1.2. Database: Ovid Embase 3](#_Toc212732318)

[Table 1.3 Database: FSTA 4](#_Toc212732319)

[Table 1.4. Database: Ovid MEDLINE(R) 5](#_Toc212732320)

[Table 1.5 Database: Science Citation Index & Social Citation Index 6](#_Toc212732321)

[Table 1.6 Database: Scopus 7](#_Toc212732322)

[Supplemental Table 2: Searches identified in peer reviewed literature August 2025 8](#_Toc212732323)

[Supplemental Table 3: Inclusion and exclusion criteria used for the eligibility assessment of all potential NECI* 9](#_Toc212732324)

[Supplemental Table 4: Excluded studies and models during the full text screening stage with reason(s) 10](#_Toc212732325)

[Supplemental Table 5: Nutritive and Environmental Combined Indices (NECIs) and Associated Creating Institutions 15](#_Toc212732326)

[Supplemental Table 6: The nutritional scoring method, nutritional indicators included in the score, and an overview of the methodology used to quantify the nutritional dimension of food-based indices for the assessment of nutritive value and environmental impact of meals and diets. 18](#_Toc212732327)

[Supplemental Table 7: The environmental scoring method, environmental indicators included in the score, and an overview of the methodology used to quantify the environmental dimension of food-based indices for the assessment of nutritive value and environmental impact of meals and diets. 32](#_Toc212732328)

[Supplemental Table 8. Overview of criterion and scoring/ranking of food-based indices for the assessment of nutritive value and environmental impact of meals and diets. 43](#_Toc212732329)

[Supplemental Table 9: Index replicability assessment 54](#_Toc212732330)

[References: 55](#_Toc212732331)

# Supplemental Table 1: Search strategies on August 18 2025 to August 21 2025

## Table 1.1 Database: CAB Abstracts

*Search strategy:*

| # ▲ | **Searches** | **Results** |
| --- | --- | --- |
| 1 | (nutri* NEAR/2 (quality or footprint or food* or profiling or density or score or index or adequacy)) or (food* NEAR/2 (label* or suppl* or consumption or choice or environment or pattern*)) or (eating NEAR/2 (indicator or score* or impact*)) or (feeding behavio$r* NEAR/2  (indicator or score* or impact*)) or (public health NEAR/2 (indicator or score* or impact*)) or (health* NEAR/2 (indicator or score* or impact*)) (Topic) | 380897 |
| 2 | (sustainab* NEAR/2 (impact or assess* or evaluat* or indicator? or health*)) or (environment* NEAR/2 (impact or assess* or evaluat* or indicator? or health*)) or (climate* NEAR/2 (impact or assess* or evaluat* or indicator? or health*)) or (land* NEAR/2 (clear* or "use*" or usage)) or (soil NEAR/2 (clear* or "use*" or usage)) or (water NEAR/2 ("use*" or usage or foot*)) or (life cycle NEAR/2 (evaluation or assessment)) or eutrophication or (carbon NEAR/2 (foot* or ecosystem)) or (fossil NEAR/2 fuels) (Topic) | 634826 |
| 3 | (model* or algorithm* or metric* or scor* or rank* or framework* or index or indices or tool* or inventor* or validation or "multi-criteria analys*" or guideline* or impact* or optim*) (Topic) | 7409757 |
| 4 | (meal* or menu* or recipe* or diet*) (Topic) | 822660 |
| 5 | 4 AND 3 AND 2 AND 1 | 5367 |
| 6 | animals or animal or mice or mus or mouse or murine or woodmouse or rats or rat or murinae or muridae or cottonrat or cottonrats or hamster or hamsters or cricetinae or rodentia or rodent or rodents or pigs or pig or swine or swines or piglets or piglet or boar or boars or sus scrofa or ferrets or ferret or polecat or polecats or “mustela putorius” or “guinea pigs” or “guinea pig” or cavia or callithrix or marmoset or marmosets or cebuella or hapale or octodon or chinchilla or chinchillas or gerbillinae or gerbil or gerbils or jird or jirds or merione or meriones or rabbits or rabbit or hares or hare or diptera or flies or fly or dipteral or drosphila or drosophilidae or cats or cat or carus or felis or nematoda or nematode or nematoda or nematode or nematodes or sipunculida or dogs or dog or canine or canines or canis or sheep or sheeps or mouflon or mouflons or ovis or goats or goat or capra or capras or rupicapra or chamois or haplorhini or monkey or monkeys or anthropoidea or anthropoids or saguinus or tamarin or tamarins or leontopithecus or hominidae or ape or apes or pan or paniscus or “pan paniscus” or bonobo or bonobos or troglodytes or pan troglodytes or gibbon or gibbons or siamang or siamangs or nomascus or symphalangus or chimpanzee or chimpanzees or prosimians or “bush baby” or prosimian or “bush babies” or galagos or galago or pongidae or gorilla or gorillas or pongo or pygmaeus or “pongo pygmaeus” or orangutans or pygmaeus or lemur or lemurs or lemuridae or horse or horses or pongo or equus or cow or calf or bull or chicken or chickens or gallus or quail or bird or birds or quails or poultry or poultries or fowl or fowls or reptile or reptilia or reptiles or snakes or snake or lizard or lizards or alligator or alligators or crocodile or crocodiles or turtle or turtles or amphibian or amphibians or amphibia or frog or frogs or bombina or salientia or toad or toads or “epidalea calamita” or salamander or salamanders or eel or eels or fish or fishes or pisces or catfish or catfishes or siluriformes or arius or heteropneustes or sheatfish or perch or perches or percidae or perca or trout or trouts or char or chars or salvelinus or “fathead minnow” or minnow or cyprinidae or carps or carp or zebrafish or zebrafishes or goldfish or goldfishes or guppy or guppies or chub or chubs or tinca or barbels or barbus or pimephales or promelas or “poecilia reticulata” or mullet or mullets or seahorse or seahorses or “mugil curema” or “atlantic cod” or shark or sharks or catshark or anguilla or salmonid or salmonids or whitefish or whitefishes or salmon or salmons or sole or solea or “sea lamprey” or lamprey or lampreys or pumpkinseed or sunfish or sunfishes or tilapia or tilapias or turbot or turbots or flatfish or flatfishes or sciuridae or squirrel or squirrels or chipmunk or chipmunks or suslik or susliks or vole or voles or lemming or lemmings or muskrat or muskrats or lemmus or otter or otters or marten or martens or martes or weasel or badger or badgers or ermine or mink or minks or sable or sables or gulo or gulos or wolverine or wolverines or minks or mustela or llama or llamas or alpaca or alpacas or camelid or camelids or guanaco or guanacos or chiroptera or chiropteras or bat or bats or fox or foxes or iguana or iguanas or “xenopus laevis” or parakeet or parakeets or parrot or parrots or donkey or donkeys or mule or mules or zebra or zebras or shrew or shrews or bison or bisons or buffalo or buffaloes or deer or deers or bear or bears or panda or pandas or “wild hog” or “wild boar” or fitchew or fitch or beaver or beav or jerboa or jerboas or capybara or capybaras (Topic) | 6108569 |
| 7 | 5 NOT 6 | 903 |
| 8 | 5 NOT 6 and 2024 or 2023 or 2022 or 2021 or 2020 or 2019 or 2018 or 2017 or 2016 or 2015 or 2014 or 2013 or 2012 or 2011 or 2010 or 2009 (Publication Years) | 901 |
| 9 | 8 and Journal Article (Document Types) | 918 |

## Table 1.2. Database: Ovid Embase

*Search strategy:*

| # ▲ | **Searches** | **Results** |
| --- | --- | --- |
| 1 | (nutri* adj2 (quality or footprint or food* or profiling or density or score or index or adequacy)).ti,kw,ab. | 42597 |
| 2 | Food Labeling/ | 14264 |
| 3 | (food* adj2 label*).ti,kw,ab. | 3733 |
| 4 | exp Food Supply/ or food supply chain.ti,kw,ab. | 27692 |
| 5 | exp Eating/ or exp Feeding Behavior/ | 245729 |
| 6 | ((eating or feeding behavio?r* or public health or health*) adj2 (indicator or score* or impact*)).ti,kw,ab. | 82530 |
| 7 | (food* adj2 (consumption or choice or environment or pattern*)).ti,kw,ab. | 41815 |
| 8 | 1 or 2 or 3 or 4 or 5 or 6 or 7 | 423866 |
| 9 | carbon footprint/ or ecosystem/ or exp biodiversity/ | 504564 |
| 10 | Greenhouse Gases/ | 9206 |
| 11 | exp fossil fuels/ | 7507 |
| 12 | exp climate change/ or greenhouse effect/ | 79080 |
| 13 | exp soil pollutants/ | 17707 |
| 14 | waste water/ or exp water pollutants/ or water supply/ | 139362 |
| 15 | exp Forests/ | 46198 |
| 16 | ((sustainab* or environment*) adj2 (impact or assess* or evaluat* or indicator? or health*)).ti,kw,ab. | 95826 |
| 17 | (land* adj2 (clear* or "use*" or usage)).ti,kw,ab. | 30038 |
| 18 | (water adj2 ("use*" or usage or foot*)).ti,kw,ab. | 24772 |
| 19 | (life cycle adj2 (evaluation or assessment)).ti,kw,ab. | 6969 |
| 20 | eutrophication.ti,kw,ab. | 10661 |
| 21 | 9 or 10 or 11 or 12 or 13 or 14 or 15 or 16 or 17 or 18 or 19 or 20 | 882037 |
| 22 | (model* or algorithm* or metric* or scor* or rank* or framework* or index or indices or tool* or inventor* or validation or "multi-criteria analys*" or guideline* or impact* or optim*). ti,kw,ab. | 12668645 |
| 23 | Menu Planning/ or Meals/ or Cookbook/ or exp Diet/ | 671251 |
| 24 | (meal* or menu* or recipe* or diet*).ti,kw,ab. | 1096094 |
| 25 | 23 or 24 | 1523636 |
| 26 | 8 and 21 and 22 and 25 | 3982 |
| 27 | exp animals/ not humans/ | 15733557 |
| 28 | 26 not 27 | 2870 |
| 29 | limit 28 to yr="2009 -Current" | 2795 |
| 30 | limit 29 to (article and journal) | 1760 |

## Table 1.3 Database: FSTA

*Search strategy:*

| # ▲ | **Searches** | **Results** |
| --- | --- | --- |
| 1 | (nutri* NEAR/2 (quality or footprint or food* or profiling or density or score or index or adequacy)) or (food* NEAR/2 (label* or suppl* or consumption or choice or environment or pattern*)) or (eating NEAR/2 (indicator or score* or impact*)) or (feeding behavio$r* NEAR/2  (indicator or score* or impact*)) or (public health NEAR/2 (indicator or score* or impact*)) or (health* NEAR/2 (indicator or score* or impact*)) (Topic) | 116779 |
| 2 | (sustainab* NEAR/2 (impact or assess* or evaluat* or indicator? or health*)) or (environment* NEAR/2 (impact or assess* or evaluat* or indicator? or health*)) or (climate* NEAR/2 (impact or assess* or evaluat* or indicator? or health*)) or (land* NEAR/2 (clear* or "use*" or usage)) or (soil NEAR/2 (clear* or "use*" or usage)) or (water NEAR/2 ("use*" or usage or foot*)) or (life cycle NEAR/2 (evaluation or assessment)) or eutrophication or (carbon NEAR/2 (foot* or ecosystem)) or (fossil NEAR/2 fuels) (Topic) | 38374 |
| 3 | (model* or algorithm* or metric* or scor* or rank* or framework* or index or indices or tool* or inventor* or validation or "multi-criteria analys*" or guideline* or impact* or optim*) (Topic) | 638798 |
| 4 | (meal* or menu* or recipe* or diet*) (Topic) | 293989 |
| 5 | 4 AND 3 AND 2 AND 1 | 2175 |
| 6 | (animals or animal or mice or mus or mouse or murine or woodmouse or rats or rat or murinae or muridae or cottonrat or cottonrats or hamster or hamsters or cricetinae or rodentia or rodent or rodents or pigs or pig or swine or swines or piglets or piglet or boar or boars or sus scrofa or ferrets or ferret or polecat or polecats or “mustela putorius” or “guinea pigs” or “guinea pig” or cavia or callithrix or marmoset or marmosets or cebuella or hapale or octodon or chinchilla or chinchillas or gerbillinae or gerbil or gerbils or jird or jirds or merione or meriones or rabbits or rabbit or hares or hare or diptera or flies or fly or dipteral or drosphila or drosophilidae or cats or cat or carus or felis or nematoda or nematode or nematoda or nematode or nematodes or sipunculida or dogs or dog or canine or canines or canis or sheep or sheeps or mouflon or mouflons or ovis or goats or goat or capra or capras or rupicapra or chamois or haplorhini or monkey or monkeys or anthropoidea or anthropoids or saguinus or tamarin or tamarins or leontopithecus or hominidae or ape or apes or pan or paniscus or “pan paniscus” or bonobo or bonobos or troglodytes or pan troglodytes or gibbon or gibbons or siamang or siamangs or nomascus or symphalangus or chimpanzee or chimpanzees or prosimians or “bush baby” or prosimian or “bush babies” or galagos or galago or pongidae or gorilla or gorillas or pongo or pygmaeus or “pongo pygmaeus” or orangutans or pygmaeus or lemur or lemurs or lemuridae or horse or horses or pongo or equus or cow or calf or bull or chicken or chickens or gallus or quail or bird or birds or quails or poultry or poultries or fowl or fowls or reptile or reptilia or reptiles or snakes or snake or lizard or lizards or alligator or alligators or crocodile or crocodiles or turtle or turtles or amphibian or amphibians or amphibia or frog or frogs or bombina or salientia or toad or toads or “epidalea calamita” or salamander or salamanders or eel or eels or fish or fishes or pisces or catfish or catfishes or siluriformes or arius or heteropneustes or sheatfish or perch or perches or percidae or perca or trout or trouts or char or chars or salvelinus or “fathead minnow” or minnow or cyprinidae or carps or carp or zebrafish or zebrafishes or goldfish or goldfishes or guppy or guppies or chub or chubs or tinca or barbels or barbus or pimephales or promelas or “poecilia reticulata” or mullet or mullets or seahorse or seahorses or “mugil curema” or “atlantic cod” or shark or sharks or catshark or anguilla or salmonid or salmonids or whitefish or whitefishes or salmon or salmons or sole or solea or “sea lamprey” or lamprey or lampreys or pumpkinseed or sunfish or sunfishes or tilapia or tilapias or turbot or turbots or flatfish or flatfishes or sciuridae or squirrel or squirrels or chipmunk or chipmunks or suslik or susliks or vole or voles or lemming or lemmings or muskrat or muskrats or lemmus or otter or otters or marten or martens or martes or weasel or badger or badgers or ermine or mink or minks or sable or sables or gulo or gulos or wolverine or wolverines or minks or mustela or llama or llamas or alpaca or alpacas or camelid or camelids or guanaco or guanacos or chiroptera or chiropteras or bat or bats or fox or foxes or iguana or iguanas or “xenopus laevis” or parakeet or parakeets or parrot or parrots or donkey or donkeys or mule or mules or zebra or zebras or shrew or shrews or bison or bisons or buffalo or buffaloes or deer or deers or bear or bears or panda or pandas or “wild hog” or “wild boar” or fitchew or fitch or beaver or beav or jerboa or jerboas or capybara or capybaras) (Topic) | 448998 |
| 7 | 5 NOT 6 | 1551 |
| 8 | 5 NOT 6 and 2024 or 2023 or 2022 or 2021 or 2020 or 2019 or 2018 or 2017 or 2016 or 2015 or 2014 or 2013 or 2012 or 2011 or 2010 or 2009 (Publication Years) | 1489 |
| 9 | 8 and Journal Article (Document Types) | 1483 |

## Table 1.4. Database: Ovid MEDLINE(R)

*Search Strategy:*

| # ▲ | **Searches** | **Results** |
| --- | --- | --- |
| 1 | (nutri* adj2 (quality or footprint or food* or profiling or density or score or index or adequacy)) .ti,kw,ab. | 35228 |
| 2 | Food Labeling/ | 4649 |
| 3 | (food* adj2 label*).ti,kw,ab. | 2735 |
| 4 | exp Food Supply/ or food supply chain.ti,kw,ab. | 18336 |
| 5 | exp Eating/ or exp Feeding Behavior/ | 271216 |
| 6 | ((eating or feeding behavio?r* or public health or health*) adj2 (indicator or score* or impact*)).ti,kw,ab. | 64638 |
| 7 | (food* adj2 (consumption or choice or environment or pattern*)).ti,kw,ab. | 32641 |
| 8 | 1 or 2 or 3 or 4 or 5 or 6 or 7 | 396520 |
| 9 | carbon footprint/ or ecosystem/ or exp biodiversity/ | 239061 |
| 10 | Greenhouse Gases/ | 2865 |
| 11 | exp fossil fuels/ | 31481 |
| 12 | exp climate change/ or greenhouse effect/ | 38380 |
| 13 | exp soil pollutants/ | 53797 |
| 14 | waste water/ or exp water pollutants/ or water supply/ | 207300 |
| 15 | exp Forests/ | 17966 |
| 16 | ((sustainab* or environment*) adj2 (impact or assess* or evaluat* or indicator? or health*)).ti,kw,ab. | 84106 |
| 17 | (land* adj2 (clear* or "use*" or usage)).ti,kw,ab. | 27693 |
| 18 | (water adj2 ("use*" or usage or foot*)).ti,kw,ab. | 21539 |
| 19 | (life cycle adj2 (evaluation or assessment)) .ti,kw,ab. | 4339 |
| 20 | eutrophication.ti,kw,ab. | 8503 |
| 21 | 9 or 10 or 11 or 12 or 13 or 14 or 15 or 16 or 17 or 18 or 19 or 20 | 653433 |
| 22 | (model* or algorithm* or metric* or scor* or rank* or framework* or index or indices or tool* or inventor* or validation or "multi-criteria analys*" or guideline* or impact* or optim*).ti,kw,ab. | 9647952 |
| 23 | Menu Planning/ or Meals/ or Cookbook/ or exp Diet/ | 342705 |
| 24 | (meal* or menu* or recipe* or diet*).ti,kw,ab. | 801966 |
| 25 | 23 or 24 | 927831 |
| 26 | 8 and 21 and 22 and 25 | 3256 |
| 27 | exp animals/ not humans/ | 6165434 |
| 28 | 26 not 27 | 2993 |
| 29 | limit 28 to yr="2009 -Current" | 2449 |
| 30 | limit 29 to journal article | 2676 |

## Table 1.5 Database: Science Citation Index & Social Citation Index

*Search strategy:*

| # ▲ | **Searches** | **Results** |
| --- | --- | --- |
| 1 | (nutri* NEAR/2 (quality or footprint or food* or profiling or density or score or index or adequacy)) or (food* NEAR/2 (label* or suppl* or consumption or choice or environment or pattern*)) or (eating NEAR/2 (indicator or score* or impact*)) or (feeding behavio$r* NEAR/2  (indicator or score* or impact*)) or (public health NEAR/2 (indicator or score* or impact*)) or (health* NEAR/2 (indicator or score* or impact*)) (Topic) | 383291 |
| 2 | (sustainab* NEAR/2 (impact or assess* or evaluat* or indicator? or health*)) or (environment* NEAR/2 (impact or assess* or evaluat* or indicator? or health*)) or (climate* NEAR/2 (impact or assess* or evaluat* or indicator? or health*)) or (land* NEAR/2 (clear* or "use*" or usage)) or (soil NEAR/2 (clear* or "use*" or usage)) or (water NEAR/2 ("use*" or usage or foot*)) or (life cycle NEAR/2 (evaluation or assessment)) or eutrophication or (carbon NEAR/2 (foot* or ecosystem)) or (fossil NEAR/2 fuels) (Topic) | 897986 |
| 3 | (model* or algorithm* or metric* or scor* or rank* or framework* or index or indices or tool* or inventor* or validation or "multi-criteria analys*" or guideline* or impact* or optim*) (Topic) | 24482796 |
| 4 | (meal* or menu* or recipe* or diet*) (Topic) | 1164723 |
| 5 | 1 AND 2 AND 3 AND 4 | 4178 |
| 6 | (animals or animal or mice or mus or mouse or murine or woodmouse or rats or rat or murinae or muridae or cottonrat or cottonrats or hamster or hamsters or cricetinae or rodentia or rodent or rodents or pigs or pig or swine or swines or piglets or piglet or boar or boars or sus scrofa or ferrets or ferret or polecat or polecats or “mustela putorius” or “guinea pigs” or “guinea pig” or cavia or callithrix or marmoset or marmosets or cebuella or hapale or octodon or chinchilla or chinchillas or gerbillinae or gerbil or gerbils or jird or jirds or merione or meriones or rabbits or rabbit or hares or hare or diptera or flies or fly or dipteral or drosphila or drosophilidae or cats or cat or carus or felis or nematoda or nematode or nematoda or nematode or nematodes or sipunculida or dogs or dog or canine or canines or canis or sheep or sheeps or mouflon or mouflons or ovis or goats or goat or capra or capras or rupicapra or chamois or haplorhini or monkey or monkeys or anthropoidea or anthropoids or saguinus or tamarin or tamarins or leontopithecus or hominidae or ape or apes or pan or paniscus or “pan paniscus” or bonobo or bonobos or troglodytes or pan troglodytes or gibbon or gibbons or siamang or siamangs or nomascus or symphalangus or chimpanzee or chimpanzees or prosimians or “bush baby” or prosimian or “bush babies” or galagos or galago or pongidae or gorilla or gorillas or pongo or pygmaeus or “pongo pygmaeus” or orangutans or pygmaeus or lemur or lemurs or lemuridae or horse or horses or pongo or equus or cow or calf or bull or chicken or chickens or gallus or quail or bird or birds or quails or poultry or poultries or fowl or fowls or reptile or reptilia or reptiles or snakes or snake or lizard or lizards or alligator or alligators or crocodile or crocodiles or turtle or turtles or amphibian or amphibians or amphibia or frog or frogs or bombina or salientia or toad or toads or “epidalea calamita” or salamander or salamanders or eel or eels or fish or fishes or pisces or catfish or catfishes or siluriformes or arius or heteropneustes or sheatfish or perch or perches or percidae or perca or trout or trouts or char or chars or salvelinus or “fathead minnow” or minnow or cyprinidae or carps or carp or zebrafish or zebrafishes or goldfish or goldfishes or guppy or guppies or chub or chubs or tinca or barbels or barbus or pimephales or promelas or “poecilia reticulata” or mullet or mullets or seahorse or seahorses or “mugil curema” or “atlantic cod” or shark or sharks or catshark or anguilla or salmonid or salmonids or whitefish or whitefishes or salmon or salmons or sole or solea or “sea lamprey” or lamprey or lampreys or pumpkinseed or sunfish or sunfishes or tilapia or tilapias or turbot or turbots or flatfish or flatfishes or sciuridae or squirrel or squirrels or chipmunk or chipmunks or suslik or susliks or vole or voles or lemming or lemmings or muskrat or muskrats or lemmus or otter or otters or marten or martens or martes or weasel or badger or badgers or ermine or mink or minks or sable or sables or gulo or gulos or wolverine or wolverines or minks or mustela or llama or llamas or alpaca or alpacas or camelid or camelids or guanaco or guanacos or chiroptera or chiropteras or bat or bats or fox or foxes or iguana or iguanas or “xenopus laevis” or parakeet or parakeets or parrot or parrots or donkey or donkeys or mule or mules or zebra or zebras or shrew or shrews or bison or bisons or buffalo or buffaloes or deer or deers or bear or bears or panda or pandas or “wild hog” or “wild boar” or fitchew or fitch or beaver or beav or jerboa or jerboas or capybara or capybaras) (Topic) | 8574996 |
| 7 | 5 NOT 6 | 2895 |
| 8 | 7 and Timespan: 2009-01-01 to 2024-04-17 | 2826 |
| 9 | 8 and Science Citation Index Expanded (SCI-EXPANDED) or Social Sciences Citation Index (SSCI) (Web of Science Index) | 3060 |

## Table 1.6 Database: Scopus

*Search strategy:*

| # ▲ | **Searches** | **Results** |
| --- | --- | --- |
| 1 | ( ( TITLE-ABS-KEY ( ( nutri* W/2 ( quality OR footprint OR food* OR profiling OR density OR score OR index OR adequacy ) ) OR ( food* W/2 ( label* OR suppl* OR consumption OR choice OR environment OR pattern* ) ) OR ( eating W/2 ( indicator OR score* OR impact* ) ) OR ( "feeding behavio*r*" W/2 ( indicator OR score* OR impact* ) ) OR ( "public health" W/2 ( indicator OR score* OR impact* ) ) OR ( health* W/2 ( indicator OR score* OR impact* ) ) ) ) AND ( TITLE-ABS-KEY ( ( sustainab* W/2 ( impact OR assess* OR evaluat* OR indicator? OR health* ) ) OR ( environment* W/2 ( impact OR assess* OR evaluat* OR indicator? OR health* ) ) OR ( climate* W/2 ( impact OR assess* OR evaluat* OR indicator? OR health* ) ) OR ( land* W/2 ( clear* OR "use*" OR usage ) ) OR ( soil W/2 ( clear* OR "use*" OR usage ) ) OR ( water W/2 ( "use*" OR usage OR foot* ) ) OR ( "life cycle" W/2 ( evaluation OR assessment ) ) OR eutrophication OR ( carbon W/2 ( foot* OR ecosystem ) ) OR ( fossil W/2 fuels ) ) ) AND ( TITLE-ABS-KEY ( ( model* OR algorithm* OR metric* OR scor* OR rank* OR framework* OR index OR indices OR tool* OR inventor* OR validation OR "multi-criteria analys*" OR guideline* OR impact* OR optim* ) ) ) AND ( TITLE-ABS-KEY ( ( meal* OR menu* OR recipe* OR diet* ) ) ) ) AND NOT ( animals OR animal OR mice OR mus OR mouse OR murine OR woodmouse OR rats OR rat OR murinae OR muridae OR cottonrat OR cottonrats OR hamster OR hamsters OR cricetinae OR rodentia OR rodent OR rodents OR pigs OR pig OR swine OR swines OR piglets OR piglet OR boar OR boars OR sus AND scrofa OR ferrets OR ferret OR polecat OR polecats OR "mustela putorius" OR "guinea pigs" OR "guinea pig" OR cavia OR callithrix OR marmoset OR marmosets OR cebuella OR hapale OR octodon OR chinchilla OR chinchillas OR gerbillinae OR gerbil OR gerbils OR jird OR jirds OR merione OR meriones OR rabbits OR rabbit OR hares OR hare OR diptera OR flies OR fly OR dipteral OR drosphila OR drosophilidae OR cats OR cat OR carus OR felis OR nematoda OR nematode OR nematoda OR nematode OR nematodes OR sipunculida OR dogs OR dog OR canine OR canines OR canis OR sheep OR sheeps OR mouflon OR mouflons OR ovis OR goats OR goat OR capra OR capras OR rupicapra OR chamois OR haplorhini OR monkey OR monkeys OR anthropoidea OR anthropoids OR saguinus OR tamarin OR tamarins OR leontopithecus OR hominidae OR ape OR apes OR pan OR paniscus OR "pan paniscus" OR bonobo OR bonobos OR troglodytes OR pan AND troglodytes OR gibbon OR gibbons OR siamang OR siamangs OR nomascus OR symphalangus OR chimpanzee OR chimpanzees OR prosimians OR "bush baby" OR prosimian OR "bush babies" OR galagos OR galago OR pongidae OR gorilla OR gorillas OR pongo OR pygmaeus OR "pongo pygmaeus" OR orangutans OR pygmaeus OR lemur OR lemurs OR lemuridae OR horse OR horses OR pongo OR equus OR cow OR calf OR bull OR chicken OR chickens OR gallus OR quail OR bird OR birds OR quails OR poultry OR poultries OR fowl OR fowls OR reptile OR reptilia OR reptiles OR snakes OR snake OR lizard OR lizards OR alligator OR alligators OR crocodile OR crocodiles OR turtle OR turtles OR amphibian OR amphibians OR amphibia OR frog OR frogs OR bombina OR salientia OR toad OR toads OR "epidalea calamita" OR salamander OR salamanders OR eel OR eels OR fish OR fishes OR pisces OR catfish OR catfishes OR siluriformes OR arius OR heteropneustes OR sheatfish OR perch OR perches OR percidae OR perca OR trout OR trouts OR char OR chars OR salvelinus OR "fathead minnow" OR minnow OR cyprinidae OR carps OR carp OR zebrafish OR zebrafishes OR goldfish OR goldfishes OR guppy OR guppies OR chub OR chubs OR tinca OR barbels OR barbus OR pimephales OR promelas OR "poecilia reticulata" OR mullet OR mullets OR seahorse OR seahorses OR "mugil curema" OR "atlantic cod" OR shark OR sharks OR catshark OR anguilla OR salmonid OR salmonids OR whitefish OR whitefishes OR salmon OR salmons OR sole OR solea OR "sea lamprey" OR lamprey OR lampreys OR pumpkinseed OR sunfish OR sunfishes OR tilapia OR tilapias OR turbot OR turbots OR flatfish OR flatfishes OR sciuridae OR squirrel OR squirrels OR chipmunk OR chipmunks OR suslik OR susliks OR vole OR voles OR lemming OR lemmings OR muskrat OR muskrats OR lemmus OR otter OR otters OR marten OR martens OR martes OR weasel OR badger OR badgers OR ermine OR mink OR minks OR sable OR sables OR gulo OR gulos OR wolverine OR wolverines OR minks OR mustela OR llama OR llamas OR alpaca OR alpacas OR camelid OR camelids OR guanaco OR guanacos OR chiroptera OR chiropteras OR bat OR bats OR fox OR foxes OR iguana OR iguanas OR "xenopus laevis" OR parakeet OR parakeets OR parrot OR parrots OR donkey OR donkeys OR mule OR mules OR zebra OR zebras OR shrew OR shrews OR bison OR bisons OR buffalo OR buffaloes OR deer OR deers OR bear OR bears OR panda OR pandas OR "wild hog" OR "wild boar" OR fitchew OR fitch OR beaver OR beav OR jerboa OR jerboas OR capybara OR capybaras ) AND ( LIMIT-TO ( DOCTYPE , "ar" ) ) AND ( LIMIT-TO ( PUBYEAR , 2009 ) OR LIMIT-TO ( PUBYEAR , 2010 ) OR LIMIT-TO ( PUBYEAR , 2011 ) OR LIMIT-TO ( PUBYEAR , 2012 ) OR LIMIT-TO ( PUBYEAR , 2013 ) OR LIMIT-TO ( PUBYEAR , 2014 ) OR LIMIT-TO ( PUBYEAR , 2015 ) OR LIMIT-TO ( PUBYEAR , 2016 ) OR LIMIT-TO ( PUBYEAR , 2017 ) OR LIMIT-TO ( PUBYEAR , 2018 ) OR LIMIT-TO ( PUBYEAR , 2019 ) OR LIMIT-TO ( PUBYEAR , 2020 ) OR LIMIT-TO ( PUBYEAR , 2021 ) OR LIMIT-TO ( PUBYEAR , 2022 ) OR LIMIT-TO ( PUBYEAR , 2023 ) OR LIMIT-TO ( PUBYEAR , 2024 ) ) | 3737 |

# Supplemental Table 2: Searches identified in peer reviewed literature August 2025

| ***Database*** | ***Interface*** | ***Coverage*** | ***Date*** | ***Hits*** |
| --- | --- | --- | --- | --- |
| CAB Abstracts | Web of Science | 1973 to present | 21/08/2025 | 918 |
| EMBASE | Ovid | 1974 to present | 21/08/2025 | 1760 |
| FSTA | Web of Science | 1969 to present | 21/08/2025 | 1483 |
| MEDLINE | Ovid | 1946 to present | 21/08/2025 | 2676 |
| Science Citation Index & Social Science Citation  Index | Web of Science Core Collection | 1945 to present | 21/08/2025 | 3060 |
| Scopus | Scopus (Elsevier) | 1996 to present | 18/08/2025 | 3737 |
| **Total:** |  |  |  | **13,634** |
| Duplicates: |  |  |  | 6,013 |
| **Final Total:** |  |  |  | 7,621 |

# Supplemental Table 3: Inclusion and exclusion criteria used for the eligibility assessment of all potential NECI*

|  | **Inclusion criteria** | **Exclusion criteria** |
| --- | --- | --- |
| **A** | Indices assessing both nutritive value and environmental impact of meals or diets. | Ranking or classification of meals and diets according to exclusively nutritional composition or exclusively environmental impact. |
| **B** | Indices for the ranking, or classification of a meal or diet. | Describes an index appropriate for the use of individual food items or food system only. |
| **C** | Describes index public health purpose. | No clear index public health purpose. |
| **D** | Displays the nutritive value and environmental impact of meals or diets to allow for ranking or classification of meals or diets for both factors - as a single metric or as multiple scores. | Indices that only list the environmental and nutritional impact in correlation, not comparison, to one another, i.e. do not allow for ranking or classification of meals or diets for both factors. |
| **E** | Indices including quantitative nutritive and environmental scores. | Indices using qualitative analysis for nutritive or environmental scoring. E.g., dietary behaviours or environmental impact, or similarity to dietary pattern (i.e. Mediterranean diet) used to classify meal or diet’s nutritional adequacy. |
| **F** | Index developed since 01 January 2014. | Describes an index developed before 01 January 2014. |
| **G** | Index is actively implemented or an index for future use. | Indices withdrawn by authors. |
| **H** | Is a peer-reviewed original article. | Is a review article, book chapter, bibliography of publications or is a non-peer reviewed original article. |
| **I** |  | No index specified. |
| **J** |  | Other (e.g., description of already included index or inability to access full paper text). |

* Letters are used to indicate the reason(s) for exclusion in the list of excluded studies and models (S4).

# Supplemental Table 4: Excluded studies and models during the full text screening stage with reason(s)

| Aceves-Martins et al. [1] | Food-Level Analysis to Identify Dietary Choices With the Highest Nutritional Quality and Lowest Greenhouse Gas Emissions and Price | B, I |
| --- | --- | --- |
| Agustina et al. [2] | Development and preliminary validity of an Indonesian mobile application for a balanced and sustainable diet for obesity management | I |
| Agyemang et al. [3] | A multi-dimensional assessment of sustainable foods and the influence of stakeholder perceptions during nutrition interventions | B, I |
| Aidoo et al. [4] | Sustainable healthy diet modeling for a plant-based dietary transitioning in the United States | I |
| Aldaya et al. [5] | Indicators and Recommendations for Assessing Sustainable Healthy Diets | H |
| Allen et al. [6] | A Delphi approach to develop sustainable food system metrics | I |
| Angelsen et al. [7] | Healthiness and environmental impact of dinner recipes vary widely across developed countries | D, I |
| Aytekin-Sahin et al. [8] | Relationship between nutrient profiles, carbon footprint and water footprint of hospital menus | I |
| Azzini et al. [9] | The health-nutrition dimension: a methodological approach to assess the nutritional sustainability of typical agro-food products and the Mediterranean diet | B |
| Bälter et al. [10] | Is a diet low in greenhouse gas emissions a nutritious diet? - Analyses of self-selected diets in the LifeGene study | D, I |
| Barré et al., [11] | Integrating nutrient bioavailability and co-production links when identifying sustainable diets: How low should we reduce meat consumption? | E |
| Baudry et al. [12] | Sustainability analysis of the Mediterranean diet: results from the French NutriNet-Sante study | I |
| Benvenuti et al. [13] | Multi-indicator design and assessment of sustainable diet plans | I |
| Bianchi et al. [14] | Evaluating foods and diets from a multi-dimensional perspective: nutrition, health and environment | I |
| Bjornarå et al. [15] | Healthy and sustainable diet and physical activity: the rationale for and experiences from developing a combined summary score | A, I |
| Broekema & Blonk. [16] | Determining the balance between nutrition and sustainability of 173 food products using stepwise optimisation | I |
| Bryan et al. [17] | An environmental impact calculator for 24-h diet recalls | A |
| Cáceres et al. [18] | Sustainable food dishes: Selection of indicators for their evaluation and communication in Chilean foodservices | D, I |
| Cambeses-Franco et al. [19] | Driving commitment to sustainable food policies within the framework of American and European dietary guidelines | D, I |
| Ceyhun Sezgin et al. [20] | Evaluation of sustainable nutrition models | I |
| Chaudhary et al. [21] | Nutritional combined greenhouse gas life cycle analysis for incorporating canadian yellow pea into cereal-based food products | B, I |
| Clodoveo et al. [22] | Towards a new food labelling system for sustainable food production and healthy responsible consumption: the Med Index Checklist | B, I |
| Clodoveo et al. [23] | Med-index: a food product labeling system to promote adherence to the Mediterranean diet encouraging producers to make healthier and more sustainable food products | B |
| Conrad et al. [24] | Data integration for diet sustainability analyses | I |
| Cozzio et al. [25] | Point-of-consumption interventions to promote virtuous food choices of tourists with self-benefit or other-benefit appeals: a randomised field experiment | I |
| Dahmani et al. [26] | Nutritional quality and greenhouse gas emissions of vegetarian and non-vegetarian primary school meals: A case study in Dijon, France | D, I |
| De Bauw et al. [27] | A combined Nutri-Score and "Eco-Score" approach for more nutritious and more environmentally friendly food choices? Evidence from a consumer experiment in Belgium | B |
| De Carvalho et al. [28] | Measuring sustainable food systems in Brazil: A framework and multidimensional index to evaluate socioeconomic, nutritional, and environmental aspects | B |
| De Laurentiis et al. [29] | EATS: a life cycle-based decision support tool for local authorities and school caterers | I, A |
| Dernini et al. [30] | Developing a methodological approach for assessing the sustainability of diets: The Mediterranean diet as a case study | I |
| Dernini et al., [31] | The Med Diet 4.0 Framework: A Multidimensional Driver for Revitalising the Mediterranean Diet as a Sustainable Diet Model | E |
| Dogbe et al. [32] | Nutritional and environmental assessment of increasing the content of fruit and vegetables in the UK diet | I |
| Donati et al. [33] | Towards a sustainable diet combining economic, environmental and nutritional objectives | I |
| Donini et al. [34] | A Consensus Proposal for Nutritional Indicators to Assess the Sustainability of a Healthy Diet: The Mediterranean Diet as a Case Study | I |
| Doran-Browne et al. [35] | Nutrient density as a metric for comparing greenhouse gas emissions from food production | B |
| Drewnowski et al. [36] | Energy and nutrient density of foods in relation to their carbon footprint | B, I |
| Drewnowski. [37] | Measures and metrics of sustainable diets with a focus on milk, yogurt, and dairy products | B, I |
| Eini-Zinab et al. [38] | Designing a healthy, low-cost and environmentally sustainable food basket: an optimisation study | I |
| El-Abbadi et al. [39] | Development of a dietary environmental index to assess nutritional quality versus environmental effect of foods and dietary patterns | J |
| Esteve-Llorens et al. [40] | Evaluating the Portuguese diet in the pursuit of a lower carbon and healthier consumption pattern | D, I |
| Esteve-Llorens et al. [41] | Towards an environmentally sustainable and healthy Atlantic dietary pattern: Life cycle carbon footprint and nutritional quality | I |
| Esteve-Llorens et al. [42] | Linking environmental sustainability and nutritional quality of the Atlantic diet recommendations and real consumption habits in Galicia (NW Spain) | D, I |
| Fernández-Ríos et al. [43] | Design of a nutrient profiling model for life cycle assessment of “superfoods” to address nutritional deficiencies and enhance environmental protection in Spain | B |
| Fresán et al. [44] | Nutritional quality and health effects of low environmental impact diets: The "seguimiento universidad de navarra" (sun) Cohort | I |
| García et al. [45] | Carbon dioxide (CO2) emissions and adherence to Mediterranean diet in an adult population: the Mediterranean diet index as a pollution level index | A, I |
| Gazan et al. [46] | A methodology to compile food metrics related to diet sustainability into a single food database: Application to the French case | B, I |
| González-García et al. [47] | Carbon footprint and nutritional quality of different human dietary choices | H |
| Gonzalez-Santana et al. [48] | Assessment of the Nutritional Composition and Environmental Impact of Menus Served in a University Cafeteria During an Academic Year | D, I |
| Graham et al. [49] | Exploring the relationship between environmental impact and nutrient content of sandwiches and beverages available in cafés in a UK university | D, I |
| Green et al. [50] | A proposed framework to develop nutrient profiling algorithms for assessments of sustainable food: the metrics and their assumptions matter | A |
| Gustafson et al. [51] | Making Healthy, Sustainable Diets Accessible and Achievable: A New Framework for Assessing the Nutrition, Environmental, and Equity Impacts of Packaged Foods | I |
| Gustafson et al. [52] | Seven Food System Metrics of Sustainable Nutrition Security | B |
| Hallström et al. [53] | Combined climate and nutritional performance of seafoods | B, I |
| Hallström et al. [54] | Sustainable meat consumption: a quantitative analysis of nutritional intake, greenhouse gas emissions and land use from a Swedish perspective | B, I |
| Harray et al. [55] | A novel dietary assessment method to measure a healthy and sustainable diet using the mobile food record: Protocol and methodology | A, I |
| Harray et al. [56] | Healthy and Sustainable Diet Index: Development, Application and Evaluation Using Image-Based Food Records | E, I |
| Herrmann et al. [57] | A comparative nutritional life cycle assessment of processed and unprocessed soy-based meat and milk alternatives including protein quality adjustment | B |
| Hobbs et al. [58] | The role of dairy products in sustainable diets: modelling nutritional adequacy, financial and environmental impacts | B, I |
| Jolliet. [59] | Integrating Dietary Impacts in Food Life Cycle Assessment | H |
| Kägi, Zschokke & Dinkel. [60] | Nutrient based functional unit for meals | A |
| Keding et al. [61] | Healthy Diets from Sustainable Food Systems: Calculating the WISH Scores for Women in Rural East Africa | J |
| Kesse-Guyot et al. [62] | Sustainability analysis of French dietary guidelines using multiple criteria | I |
| Kesse-Guyot et al. [63] | Nutritionally adequate and environmentally respectful diets are possible for different diet groups: an optimized study from the NutriNet-Sante cohort | I |
| Kluczkovski et al. [64] | An Environmental and Nutritional Evaluation of School Food Menus in Bahia, Brazil That Contribute to Local Public Policy to Promote Sustainability | I |
| Kyttä et al. [65] | Extending the product-group-specific approach in nutritional life cycle assessment | B |
| Kyttä et al. [66] | Product-group-specific nutrient index as a nutritional functional unit for the Life Cycle Assessment of protein-rich foods | B |
| Kyttä et al. [67] | Integrating nutrition into environmental impact assessments reveals limited sustainable food options within planetary boundaries | B |
| Lima et al. [68] | AVACARD – Menu evaluation index: Construction and validation | E |
| Liyanapathirana et al. [69] | Nutrient-sensitive approach for sustainability assessment of different dietary patterns in Australia | I |
| Long et al. [70] | Carbon footprint and embodied nutrition evaluation of 388 recipes | I |
| Lukas et al. [71] | The nutritional footprint: An innovative management approach for the food sector | J |
| Martins et al. [72] | The SHED Index: A Validation Study to Assess Sustainable HEalthy Diets in Portugal | A, I |
| Marty et al. [73] | Development and validation of a web application to collect food supply data associated with their nutritional composition and environmental impacts | I |
| McAuliffe et al. [74] | Protein quality as a complementary functional unit in life cycle assessment (LCA) | B |
| Mungkung et al. [75] | The nutrition-environment nexus assessment of Thai Riceberry product for supporting environmental product declaration | B, I |
| Nakamura & Itsubo. [76] | Environmental and health-related lifecycle impact assessment of reduced-salt meals in Japan | A, I |
| Nguyen et al. [77] | Environmental impact and nutrient adequacy of derived dietary patterns in Vietnam | I |
| O’Malley et al. [78] | Vegan vs Paleo: carbon footprints and diet quality of 5 popular eating patterns as reported by US consumers | I |
| Perignon et al. [79] | How low can dietary greenhouse gas emissions be reduced without impairing nutritional adequacy, affordability and acceptability of the diet? A modelling study to guide sustainable food choices | I |
| Petruzzelli et al. [80] | Towards sustainable school meals: integrating environmental and cost implications for nutritious diets through optimisation modelling | I |
| Philippi Rosane et al. [81] | Development of a methodology to compare and evaluate health and sustainability aspects of dietary intake across countries | B |
| Pink et al. [82] | The effects of presenting health and environmental impacts of food on consumption intentions | I |
| Potter et al. [83] | Effects of environmental impact and nutrition labelling on food purchasing: an experimental online supermarket study | I |
| Prosperi. [84] | Sustainability and food and nutrition security: an indicator-based vulnerability and resilience approach for the Mediterranean Region | I |
| Rocha & Viegas. [85] | KIMEHS-proposal of an index for qualitative evaluation of children's menus-a pilot study | A |
| Röös et al. [86] | Introducing a comprehensive and configurable tool for calculating environmental and social footprints for use in dietary assessments | A |
| Rosi et al. [87] | Nutritional Composition and Environmental Impact of Meals Selected in Workplace Canteens before and after an Intervention Promoting the Adherence to the Mediterranean Diet | D, I |
| Ruini et al. [88] | Working toward Healthy and Sustainable Diets: The “Double Pyramid Model” Developed by the Barilla Center for Food and Nutrition to Raise Awareness about the Environmental and Nutritional Impact of Foods | E, I |
| Saarinen et al. [89] | Taking nutrition into account within the life cycle assessment of food products | B |
| Saleki et al. [90] | The evaluation of menus' adherence to sustainable nutrition and comparison with sustainable menu example in a Turkish university refectory | D, I |
| Schumacher et al. [91] | Development of a Scoring Tool for Australian Rural Food Retail Environments | A, I |
| Seconda et al. [92] | Comparing nutritional, economic, and environmental performances of diets according to their levels of greenhouse gas emissions | D |
| Simon et al. [93] | Assessment of the diet-environment-health-cost quadrilemma in public school canteens. an LCA case study in Galicia (Spain) | D, I |
| Singh-Povel et al. [94] | Nutritional content, protein quantity, protein quality and carbon footprint of plant-based drinks and semi-skimmed milk in the Netherlands and Europe | B, I |
| Sonesson et al. [95] | Protein quality as functional unit – A methodological framework for inclusion in life cycle assessment of food | B |
| Strasburg et al. [96] | Nutritional quality and environmental impact by the inputs of a university canteen in Uruguay | D, I |
| Strid et al. [97] | Sustainability indicators for foods benefiting climate and health | B |
| Sturtewagen et al. [98] | Resource use profile and nutritional value assessment of a typical Belgian meal, catered or home cooked, with pork or QuornTM as protein source | D, I |
| Sugimoto et al. [99] | Exploring culturally acceptable, nutritious, affordable and low climatic impact diet for Japanese diets: proof of concept of applying a new modelling approach using data envelopment analysis | I |
| Takacs, et al. [100] | An integrated assessment of the environmental and nutritional impacts of different types of meals using life cycle assessment | J |
| Tepper et al. [101] | The SHED Index: a tool for assessing a Sustainable HEalthy Diet | A, I |
| Totland et al. [102] | Development and evaluation of an index assessing adherence to the Norwegian food-based dietary guidelines: the Norwegian Dietary Guideline Index (NDGI) | A |
| Travassos et al. [103] | Environmental and nutritional perspective of a more sustainable meat consumption in Brazil | B, I |
| Van der Kamp & Temme. [104] | Plant-Based Lunch at Work: Effects on Nutrient Intake, Environmental Impact and Tastiness—A Case Study | D |
| Van Dooren et al. [105] | Proposing a Novel Index Reflecting Both Climate Impact and Nutritional Impact of Food Products | D |
| Van Dooren & Aiking [106] | Defining a nutritionally healthy, environmentally friendly, and culturally acceptable Low Lands Diet | I |
| Van Kernebeek et al. [107] | The effect of nutritional quality on comparing environmental impacts of human diets | H |
| Van Mierlo et al. [108] | A model for composing meat replacers: Reducing the environmental impact of our food consumption pattern while retaining its nutritional value | B |
| Walker et al. [109] | Methodology and optimization tool for a personalized low environmental impact and healthful diet specific to country and season | I |
| Walker et al. [110] | Comparison of Environmental Impact and Nutritional Quality among a European Sample Population – findings from the Food4Me study | D |
| Wilson et al. [111] | Foods and Dietary Patterns That Are Healthy, Low-Cost, and Environmentally Sustainable: A Case Study of Optimization Modeling for New Zealand | I |
| Wrieden et al. [112] | Is the UK diet sustainable? Assessing the environmental impact, cost and nutritional quality of household food purchases | I |
| Wright et al. [113] | Environmental and nutritional assessment of young children's diets in Norway: comparing the current diet with national dietary guidelines and the EAT-Lancet reference diet | I |
| Xu et al. [114] | Effects of different functional units on carbon footprint values of different carbohydrate-rich foods in China | B, I |
| Yue et al. [115] | Optimization of residents' dietary structure with consideration of greenhouse gas mitigation and nutritional requirements | I |
| Zakowska-Biemans et al. [116] | Searching for a measure integrating sustainable and healthy eating behaviors | I |
| Zhan et al. [117] | Planetary Health Diet Index Trends and Associations with Dietary Greenhouse Gas Emissions, Disease Biomarkers, Obesity, and Mortality in the United States (2005–2018) | D, I |
| Zhao et al. [118] | A comparative study on carbon footprints between wheat flour and potato in China considering the nutrition function of foods | B, I |
| Zulkefli & Moy. [119] | Development and validation of a sustainable diet index among Malaysian adults: Protocol | A |

# Supplemental Table 5: Nutritive and Environmental Combined Indices (NECIs) and Associated Creating Institutions

| **Publication citation** | **Index name** | **Type of associated creating institution** | **Name of institution** |
| --- | --- | --- | --- |
| **Aidoo et al. (2023)**  **[120]** | **Environmental Impact Weighted Daily Value score (EIWDVs) model** | University | McGill University,  University of Arkansas System Division of Agriculture |
| **Bach et al. (2023)**  **[121]** | **Sustainability Score** | University and education company | Minerva University,  Minerva Project |
| **Batlle-Bayer et al. (2019)**  **[122]** | **Index not clearly named** | University | University Pompeu Fabra, University of Cantabria |
| **Batlle-Bayer et al. (2020)**  **[123]** | **Index not clearly named** | University | Universitat Pompeu Fabra, University of Cantabria |
| **Cooreman-Algoed et al. (2020)**  **[124]** | **Index not clearly named** | University | Ghent University |
| **Costa da Silva et al. (2023)**  **[125]** | **Healthy and sustainable preparation index (HSPI)** | University | Federal University of Rio de Janeiro, Fulminese Federal University |
| **Dourmad et al. (2019)**  **[126]** | **Index not clearly named** | Higher education institution | Agrocampus Ouest |
| **Fresán et al. (2020)**  **[127]** | **[new] sustainable diet index (SDI)** | University and research institute | University of Navarra,  Instituto de Salud Carlos III, Instituto de Salud Pública y Laboral de Navarra,  Loma Linda University,  Harvard University,  Navarra Institute for Health Research. |
| **Goss and Sharewood (2024) [128]** | **Performance-weighted environmental sustainability (PwES)** | University | Department of Chemistry, Green Chemistry Centre of Excellence, University of York, Heslington, North Yorkshire, UK |
| **Guido et al. (2020)**  **[129]** | **The Food-Triad** | University | Universidade Federal de São Paulo |
| **Haupt et al. (2016)**  **[130]** | **Eco-nutritional-efficiency (ENE)** | University | Universitat Politècnica de València |
| **Hooker et al. (2024)**  **[131]** | **Sustainability Index** | University | Washington State University |
| **Kong et al. (2025) [132]** | **Food consumption sustainability index** | University | Nanjing Agricultural University, Zhejiang Ocean University, Beijing Normal University |
| **Li et al. (2023)**  **[133]** | **Comprehensive assessment index (CAI)** | Research institute and university | Institute of Geographic Sciences and Natural Resources Research, University of Chinese Academy of Sceinces, Univeristy of Pennsylvania,  University of Surrey, Hotelschool The Hague |
| **Lukas et al. (2016)**  **[134]** | **Nutritional Footprint** | University, research institute, and private company | Wuppertal Institute for Climate, Environment and Energy,  Institut für nachhaltiges Wirtschaften gGmbH,  ITMO University,  Aalto University,  D-mat Ltd,  Folkwang University of Arts |
| **Meier et al. (2024) [135]** | **Planet Health Conformity Index (PHC)** | University and consultancy | Institute for Sustainable Agriculture and Food Economics (La Trobe University)  Corsus – corporate sustainability & Corsus Research |
| **Rӧӧs et al. (2015)**  **[136]** | **Index not clearly named ‘Method 1’& ‘Method 2’*** | University | Swedish University of Agricultural Sciences |
| **Schaubroeck et al. (2018)**  **[137]** | **Index not clearly named** | University | Ghent University,  Luxembourg Institute of Science and Technology (LIST) |
| **Seconda et al. (2019)**  **[138]** | **Sustainable diet index (SDI)** | University and independent research group | Université Paris, Aix Marseille Université, Solagro |
| **Sonesson et al. (2019)**  **[139]** | **Dietary dependent nutrient quality index (NQI)** | Research institute | Research Institutes of Sweden |
| **Strid et al. (2021)**  **[140]** | **Parallel & Integrated climate-nutrient score** | University and research institute | University of Gothenburg and Research Institutes of Sweden (RISE) and Umea University. |
| **Takacs et al. (2025) [141]** | **Index not clearly named** | University | University College London (UCL Department of Civil, Environmental and Geomatic Engineering, Centre for Urban Sustainability and Resilience, UCL Division of Medicine, Institute of Cardiovascular Science) |
| **Trijsburg et al. (2021)**  **[142]** | **World Index for Sustainability and Health (WISH)** | University and non-profit organisations | Wageningen University, Research Bioversity International,  US AID |
| **van Dooren et al. (2014)**  **[143]** | **Index not clearly named** | Non-governmental organisation, consultancy, and university | Netherlands Nutrition Centre, Blonk sustainability,  VU university |
| **Werner, Flysjo & Tholstrup. (2014)**  **[144]** | **The Nutrient Density of Climate Impact (NDCI) index** | University and comapny | University of Copenhagen,  Arla Foods |

*Index not clearly named, however, author distinguishes between the two presentation methods of the index by defining each.

| Supplemental Table 6: The nutritional scoring method, nutritional indicators included in the score, and an overview of the methodology used to quantify the nutritional dimension of food-based indices for the assessment of nutritive value and environmental impact of meals and diets. | | | | | | |
| --- | --- | --- | --- | --- | --- | --- |
| **Index Name*** | **Nutritional Scoring Method** | | | **Nutritional indicators included** | | **Methodology** |
|  | **Name** | **Functional unit** | **Validation** | **Nutrients** | **Food groups** |  |
| **Environmental Impact Weighted Daily Value score (EIWDVs) model**  **[120]** | Weighted Daily Value Score (WDVS). | Not clearly stated. | Nutrient list is a modified version of the Standard Reference Legacy of the United States Department of Agriculture (166). | Energy, protein, total fat, saturated fat, cholesterol, carbohydrates, total dietary fiber, total sugars, calcium, iron, magnesium, phosphorous, potassium, zinc, vitamin C, vitamin B1, vitamin B2, vitamin B3, vitamin B6, vitamin B9, vitamin B12, vitamin A, vitamin D, vit K and Vit E |  | The WDVs start with deriving the Daily Value Scores (DVs). DVs are calculated as ratios of specific nutrient quantity (qa) to the US-FDA based Daily Value (Qe) for that nutrient, based on the 2000 calorie daily intake recommendation (as per the below equation):  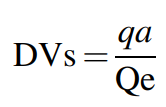  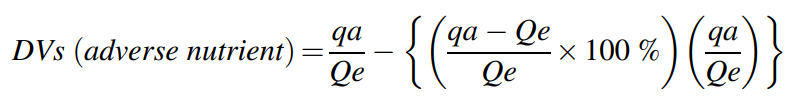However, in the case of an adverse nutrient (e.g., cholesterol, saturated fatty acids and sodium) where q_a_ is 20% higher than their Qe the following equation is used:  The ratio of the sum of the daily value scores (Sum of DVs) to the total expected daily value score (DVte) for the product is calculated as the Weighted Daily Value Score (WDVs) (as per the below equation).  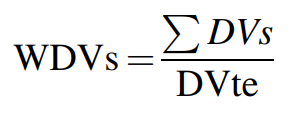   - DVs = Daily Value score for specific nutrient.   DVte = Expected daily value score (Equivalent to the total number of individual nutrients considered). |
| **Sustainability Score**  **[121]** | SAIN:LIM | per 100g | Nutrient profiling approach proposed by the French Food Standard Agency and validated by the World Health Organization (164, 165). | [SAIN]^†^ protein, fiber, calcium, vitamin C, and iron.  [LIM] fatty acids, added sugars, and sodium. |  | The SAIN nutrients are weighted based on the minimum dietary recommended intake. LIM nutrients are weighted based on the maximum recommended values. SAIN is divided by LIM to achieve a final total score.   - SAIN values greater than or equal to five indicate a positive nutrient density. - LIM values ≥7.5 indicate the high presence of nutrients that should be limited.   In the Sustainability Score the SAIN:LIM ratio is calculated for every food item based on the items category. The SAIN:LIM ratio is then multiplied by the proportion of that item within the diet, which is calculated by dividing the quantity of the item by the total amount of food in the diet.   - 1 point awarded if diets SAIN:LIM ratio is higher than the overall total/mean average of all diets   Zero points are awarded if the diets SAIN:LIM ratio is lower than the overall total/mean average of all diets. |
| **Batlle-Bayer et al. (2019)**  **[122]** | Energy Score (ES) & Nutritional Score (NS based on Nutrient Rich Diet Index (NRD9.3) | g/day | ES (149).  NS - NRD9.3 (100) based on NRF9.3 (150). | [encourage] Protein, fiber, vitamin A, vitamin C, vitamin E, calcium, iron,  magnesium, potassium  [limit] saturated fats, added sugar, and sodium |  | For diets with a lower caloric intake than recommendations, α is equal to the Energy Score (ES) defined as the ratio between the average daily energetic intake (ADEI) and the recommended one (RDEI) (below equation):  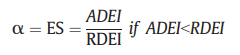  When caloric intake is larger than the recommended, α is the inverse of the ES, as per the below equation.  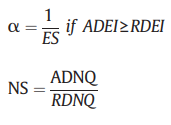  The Nutritional Score (NS) is the ratio between the nutritional quality of the average Daily diet (ADNQ) and the Recommended one (RDNQ) (below equation). Following this approach, the best score (1) is given to the recommended intake.  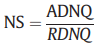  To evaluate the nutritional quality, the Nutrient Rich Diet 9.3 index was used. |
| **Batlle-Bayer et al. (2020)**  **[123]** | Energy Score (ES) & Nutritional Score (NS) assessed via Nutrient Rich Diet Index (NRD9.3) | Kg per yr^−1^ | ES (149).  NS – based on NRD9.3 (100, 150). | [encourage] Protein, fiber, vitamin A, vitamin C, vitamin E, calcium, iron,  magnesium, potassium  [limit] saturated fats, added sugar, and sodium |  | ES is the ratio between the lower daily energy intakes (DEdiet) and the recommended daily energy intake (DErec) (149, 151).  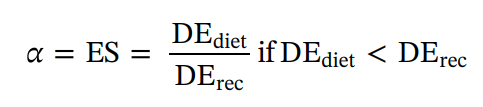  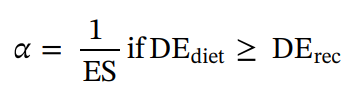  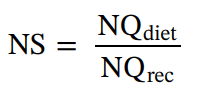NS determines the quality of their diet and is calculated as the ratio between the nutritional quality of the diet (NQdiet) and the recommended one (NQdiet), which has a best score of 1.  The nutritional quality is assessed using the NRD9.3 index. |
| **Cooreman-Algoed et al. (2020)**  **[124]** | Weighted Nutrient Density Score (WNDS) & nutritional thresholds. | WNDS - g per 100kcal (protein, fiber, SFA, unsaturated fat and added sugar).  mg per 100kcal (sodium).  Nutritional threshold - per meal. | WNDS – not clearly stated.  Nutritional threshold previously used in canteen meal assessment (142, 143). | [encourage] protein, fiber, and unsaturated fat  [limit] saturated fat, added sugar, and sodium |  | To quantify the WNDS, the nutritional composition of the meal components the canteen product sheets were consulted, and the below equation was used:  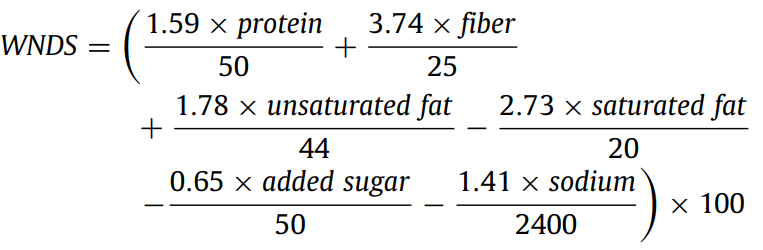  Nutritional thresholds: nutrition composition of meals compared to four nutritional threshold values (144, 145).   - Total fat content should be lower than 35% of the total energy content; - Saturated fat content should be lower than 13% of the total energy content; - Sodium content should be lower than 2.2 mg/kcal; - Energy content of the meal should be lower than 744 kcal.   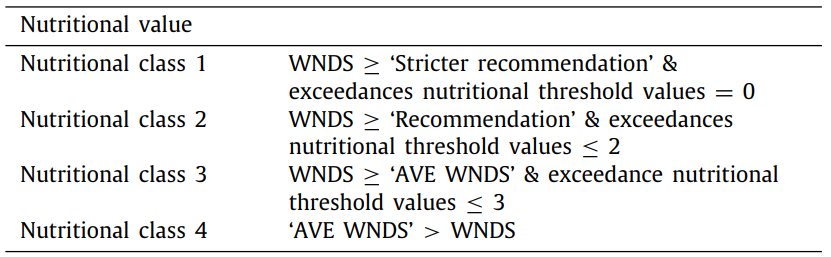The below ranking system for the WNDS and nutritional threshold combined was used: |
| **Healthy and sustainable preparation index (HSPI)**  **[125]** | Energy Density (ED) & Nutritional Density (ND) | ED – kcal per g.  ND – per portion. | ED calculation previously described (132).  ND – not clearly stated. | Retinol equivalents, vitamin D, vitamin E, thiamine, riboflavin, vitamin C, niacin, vitamin B6, vitamin B12, folate, phosphorus, iron, potassium, calcium, magnesium, selenium and zinc |  | ED: dividing the total energy value of the preparation (meal) (Kcal) by its weight in grams (g).  Centres for Disease Control and Prevention (CDC) classification used (133):   - high energy density (4 to 9 kcal/g), - medium energy density (1.5 to 4 kcal/g), - low energy density (0.7 to 1.5 kcal/g) - very low energy density (0 to 0.6 kcal/g)   ND: the content of vitamins and minerals present in each food were summed and then divided by the weight of the portion of the preparation. |
| **Dourmad et al. (2019) [126]** | Not clearly stated. | per 100g | Not clearly stated. | Dry matter, energy, protein, fat, carbohydrates, fiber, saturated fat, monounsaturated fat, polyunsaturated fat, omega3 AG, g omega6, eicosapentaenoic acid, docosahexaenoic acid, linoleic acid, linolenic acid, lauric acid, myristic acid, pentadecanoic acid, palmitic acid, calcium, phosphorus, iron, zinc, selenium, iodine. |  | Nutritional values of foods on menus previously reported were determined from the French reference table on the nutritional composition of foods. |
| **[new] sustainable diet index (SDI)**  **[127]** | Nutritional Quality Index | Not clearly stated. | 2015–2020 Dietary Guideline for Americans Index (2015DGAI) previously applied (152) & based on 2015-202 Dietary Guidelines for Americans (DGA) (153). |  | [sub-score one]  vegetables (with 5 subgroups: dark green vegetables, red or orange vegetables, starchy vegetables, other vegetables, and legumes), fruit, vegetable and fruit diversity, cereals, meat and eggs, fish and seafood, dairy products, and added sugar.  [sub-score 2] included components such as percentage of whole grains, fiber intake, total fat, saturated fatty acids, cholesterol, low-fat products (dairy and meat), sodium intake, alcohol consumption, and trans-fat intake. | Two scores are included in the 2015DGAI.   1. Food consumption according to recommended energy intake 2. The absolute quantity or the percentage of energy of heathy choice suggestions   The 2015DGAI includes 21 components and is scored from 0 (lowest adherence) to 1 (highest adherence) and scores are summed. Sub score 2 is scored inversely. |
| **Performance-weighted environmental sustainability (PwES) [128]** | Not clearly stated. | Per kg | PwES previous method referenced [145]. | Energy (kcal), protein, fiber, and  portions of fruit and vegetables (to represent micronutrients, where  one portion is 80 g of a fruit or vegetable). |  | Safe operating space for the UK food supply is shown in Eq 1-3 in paper.  The SoSOS for UK food supply was then separated into the contributing functions of energy, protein, fiber, and  portions of fruit and vegetables.  The nutrition of each foodstuff was  converted into a nutritional unit (NU, per kg) after dividing by demand for that nutrition (calories, protein, fiber, or portions of fruit and vegetable).  The example of broccoli calories is provided in the paper and below.  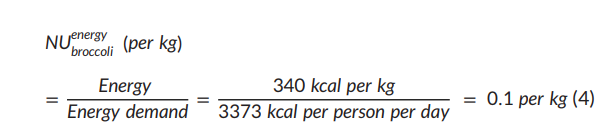  The sum of the nutrition per food item or ingredient is used to evaluate meals. |
| **The Food-Triad**  **[134]** | Nutrient Percentage | per 100g | Nutrient percentage (154). | [beneficial] protein, fiber, vitamin A, vitamin C, vitamin D, vitamin E, vitamin K, vitamin B6, B vitamin 12, calcium, iron, magnesium, zinc, phosphorous, potassium, copper, selenium, monounsaturated fat.  [adverse] trans-fat, saturated fat and sodium, carbohydrate^*^ |  | The nutrient percentage was calculated in relation to the daily intake recommended for an adult, and its representativeness in the chosen food. The following equation for nutritional dimension (Nd) was used:  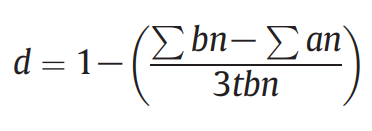  *bn* represents the punctuation for each beneficial nutrient^*^.  *an* represents nutrients related to adverse health effects and its respective values based in percentage from a portion.   - 3 points were added for the Nd when the percentage of a nutrient in a portion of 100 g was higher than 20% of the recommended daily intake. - 2 points were added for the Nd when the percentage of a nutrient in a portion of 100 g was between 10 and 20% (including 20%).   1 point was added for the Nd when the percentage of a nutrient in a portion of 100 g was between 0–10% (including 10%). |
| **Eco-nutritional-efficiency (ENE)**  **[130]** | Extend of Compliance Indicator (ECI) | kcal per edible portion. | Not clearly stated. | Energy, protein, fat and carbohydrates |  | Nutrient values obtained from database (USDA, 201118 ; dietowin® 7.3 software). And nutritional assessment of each daily menu was carried out by two approaches.  Approach one: absolute macronutrient content.  Approach two: recommended daily intake (RDI) of fractions of macronutrients.  RDI used (146):   - 12-15% of all calories should be delivered by proteins - 30-35% of all calories should be delivered by fat - 50-55% of all calories should be delivered by carbohydrates.   Nutritional performance was calculated using an ECI following the below equation:  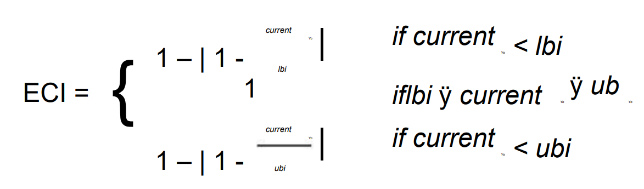  Values that lie within the ECI (ECI=1) represent a nutrient content within the range of the EDI.  Values below the ECI (ECI<1) represent if the current content of nutrient *I* (percentage of total calorific input) is below the lower bound (lb) or above the upper bound (ub). |
| **Sustainability index**  **[131]** | Nutrient Rich Foods Index 9.3 (NRF9.3) | Per 100kcal | NRF9.3 development and validation (139, 150). | [encourage] Protein, fiber, vitamin A, vitamin C, vitamin E, calcium, iron,  magnesium, potassium  [limit] saturated fats, added sugar, and sodium |  | The NRF9.3 was calculated using the following formula:  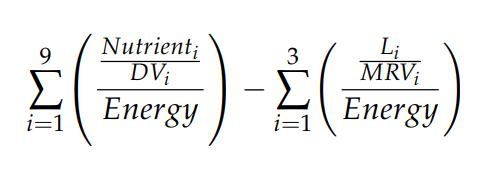   - Nutrient*i* is the amount of nutrient to encourage *i* in 100 g of the food item. - DV*i* is the daily value amount for nutrient *i*. - Energy is the number of calories in 100g of the food item. - L*i* is the amount of nutrient to limit *i* in 100 g of the food item.   MRV*i* is the maximum recommended value for the nutrient to limit *i*. |
| **Food consumption sustainability index [132]** | Nutritional Quality | Per captia | Each nutritional quality indicator used was referenced [146-148] |  | Cereals  Edible oils  Vegetables  Meat and poultry products  Aquatic products  Eggs  Dairy products  Dried and fresh fruits  Sugar | 1. Per capita stable food consumption = Grains include cereals, potatoes, beans 2. Dietary structure ratio = Animal-based food consumption/ plant-based 3. Nutritional balance gap degree = Food actual consumption - recommended intake 4. Berry index of dietary diversity = Calculated by the Simpson index |
| **Comprehensive assessment index (CAI)**  **[133]** | Diet Balance Index_16 (DBI_16) & Dietary Diversity Score (DDS). | DBI - per food group based on weight consumed. | DBI_16 (134).  DDS (135-137). |  | Cereal, vegetable and fruit, milk and soybean products, animal food, empty energy food, condiments, diet variety, and drinking water | DBI_16 reflects dietary nutrition and health status of residents according to the consumption level of different foods (n=8 indicators). DBI_16 includes four sub-indicators: total score (TS), high bound score (HBS), low bound score (LBS) and diet quality distance (DQD). The DQD was adopted in the CAI.  DQD: the cumulative value of the absolute value of eight indicators, reflecting the comprehensive diet quality.  DDS is a measure of diet diversity (DD).  Where food intake data was limited dietary diversity (DD) was used.  DDS was the frequency of consumption of different types of foods consumed (n=8 indicators). |
| **Nutritional Footprint**  **[134]** | Health Indicator (nutritional footprint health (NFhealth)) | 100g or per portion (meal) | Threshold levels referenced (156, 157). | Energy, sodium, dietary fiber, saturated fat |  | 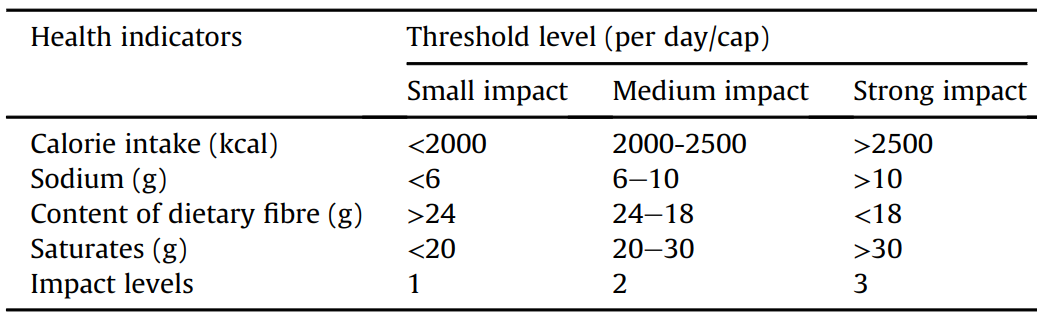The share of the meals nutritional value of the whole day is calculated, and results are transferred for each nutrient into an effect level (1-3) as seen below.  The average of the meals four effect levels is then calculated using the below equation:  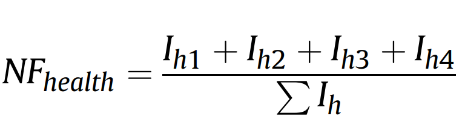 |
| **Planet Health Conformity Index (PHC) [135]** | Not clearly stated - based on nutriRECIPE | Per 100g | nutriRECIPE previous method referenced [149]. | Maco (Protein, SFAs, MUFAs + PUFAs, dietary fiber)  Vits ( Thiamine, Riboflavin, Vitamin B6, Folate, Vitamin B12, Vitamin C, Vitamin D, Vitamin E)  Minerals (Salt, calcium, Magnesium, Iron, Zinc, Iodine). |  | Use of previously described NutriRECIPE methodology, with the exclusion of sugar.  Summation of nutrients or nutritional sub-scores. Then weighting of the nutrients using a distance-to-target approach to valorize nutrients with higher public health relevance.  Before standardising for energy to make the food/meal independent of portion size and including capping where necessary. |
| **Rӧӧs et al. (2015)**  **[136]** | Method One: Nutrient Intake | Not clearly stated. | Not clearly stated. | Energy, total fat, saturated fats, monounsaturated fats, polyunsaturated fats, cholesterol, protein, carbohydrates, fiber, salt, vitamin B12, folate, vitamin C, vitamin E, vitamin D, calcium, phosphorus, iron, zinc |  | The score expresses the normalised content of different nutrients in the diet based on recommended intake (RI) or upper levels for average daily intake (Upper Intake Levels, UL) according to the Nordic Nutrition Recommendations (NNR) (138). |
| **Rӧӧs et al. (2015)**  **[136]** | Method Two: Nutrient-Rich Diet Score 9.3 (NRD 9.3), NRD 11.4-Riksmaten & NRD10.3-LCHF | Not clearly stated. | All nutrient density scores are based on the NRD9.3 index (100, 139). | NRD 9.3: [encourage] Protein, fiber, vitamin A, vitamin C, vitamin E, calcium, iron, magnesium, potassium, [limit] saturated fats, added sugar, and sodium.  NRD11.4 Riksmaten: [encourage] protein, fiber, vitamin A, vitamin C, vitamin E, vitamin D, vitamin B9, calcium, iron, magnesium, potassium, [limit] saturated fats, added sugar, sodium, phosphorus.  NRD 10.3-LCHF: [encourage] protein, vitamin A, vitamin C, vitamin E, vitamin D, vitamin B9, calcium, iron, magnesium, potassium, [limit] added sugar, sodium and phosphorus. |  | The nutritional quality of the diets was considered by calculating their nutrient density score (all based on the NRD9.3 index) using the below equation:  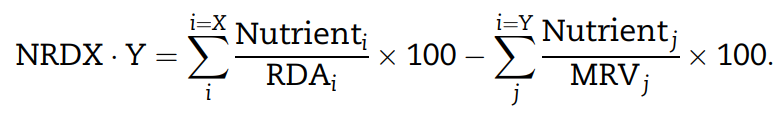   - X is the number of qualifying nutrients - Y is the number of disqualifying nutrients - Nutrienti/j is the average (total yearly intake/365) daily intake of nutrient i or j - RDA is the Recommended Daily Allowance of qualifying nutrient i - MRV is the Maximum Recommended Value of the nutrient to limit j.   RDA and MRV are defined using reference values from NNR (140). |
| **Schaubroeck et al. (2018)**  **[137]** | Not clearly stated | Nutrients (g) per 100kcal | Reference to other nutrition criteria to underpin Schaubroeck et al. approach (154, 155). | Energy, protein, fat, saturated fat, carbohydrates, sugar, salt (NaCl). |  | One penalty point is allocated for each parameter which is not in line with a predefined criterion and a nutritional score is then calculated by simply adding up all points, plus one extra, to obtain a score from 1 to x+1, with x the number of criteria. The formula for nutritional score is:  $Nutritional score of meal=1+number of nutritional criteria met$  This leads to a score range from 1 (positive) to 8 (negative). |
| **Sustainable diet index (SDI)**  **[138]** | PANDiet index & energy | PANDiet – % or (x/100)  Energy – kJ per day. | Evaluation of PANDiet index (141). | proteins, total carbohydrates, total fats, PUFA, fiber, vitamin A, vitamin B1, vitamin B2, vitamin B3, vitamin B6, vitamin B9, vitamin B12, vitamin C, vitamin D and vitamin E, calcium, magnesium, zinc, phosphorus, potassium, iron, saturated fats, cholesterol and sodium. |  | “The PANDiet reflects the probability of adequacy to French recommendations for twenty-four nutrients.  The PANDiet includes two sub-scores:  an adequacy sub-score assessing the probability that nutrient intake satisfied the requirements (above a reference value) and a moderate sub-score assessing the probability that nutrient intake was not excessive (over a reference value). The PANDiet is the average of adequacy and moderate sub-scores.”  Energy is the absolute value of the difference between energy need and intake.  Points (0-5) are allocated as follows for the PANDiet:   - 1 point: ind ≤60·7 - 2 points: 60·7 <ind≤64.4 - 3 points: 64.4<ind≤68.2 - 4 points: 68.2<ind≤ 72·8 - 5 points: ind>72.8   Points (0-5) are allocated as follows for energy are as follows:   - 1 point: ind >4259 - 2 points: 4259≤ind<2849 - 3 points: 2849≤ind<1812 - 4 points: 1812≤ind<883   5 points: ind≤883 |
| **Dietary dependent nutrient quality index (NQI)**  **[139]** | Nutrient Quality Index (NQI) developed from Nutrient Rich Foods Index 9.3 (NRF 9.3) | NQI = per product (g)  NRF9.3 = per 100kcal | NRF9.3 (150).  NQI (158).  Requirements referenced (159). | [qualifying] Protein, fiber, vitamin A, vitamin C, vitamin E, calcium, iron,  magnesium, potassium  [disqualifying] saturated fat, sodium, added sugar |  | NQI is based on the NRF9.3 as includes the same nine qualifying nutrients and the three disqualifying nutrients, however qualifies nutrients are not capped. The NQI is calculated as follows:   - 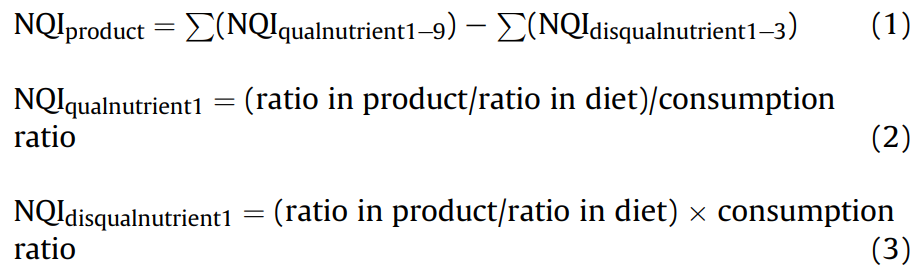Ratio in product = total content of nutrient in mass divided by total mass of product (g/g) - Ratio in diet = total intake of nutrient in mass divided by total dietary intake in mass (g/g) - Consumption ratio = ratio of dietary intake to dietary need for the nutrient   If the NQI=1, then nutrient intake that matches requirements.  If the NQI<1, then there is a deficiency of that nutrient in the diet.  If the NQI>1, then there is an surplus of the nutrient in the diet. |
| **Parallel**  **climate-nutrient score**  **[140]** | Nutrient Rich Foods Index 11.3 (NRF11.3) | per 100kcal and per portion size | NRF index (139, 167).  Evaluation of methods (167). | [encourage] Protein, fiber, vitamin A, vitamin C, vitamin E, vitamin D, calcium, iron,  magnesium, potassium, folate  [limit] saturated fats, added sugar, and sodium |  | The nutrient density of the included food products was calculated using different variants of the Nutrient Rich Foods (NRF) index. NRF11.3 assigns a nutrient density score based on 11 nutrients to encourage and 3 nutrients to discourage using the below equation:  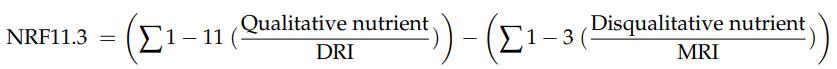   - DRI = A mean of sex and age specific dietary reference intakes - MRI = Maximum recommended intakes.   Both as per the NNR. |
| **Integrated climate-nutrient score**  **[140]** | Nutrient Rich Foods Index 11.3 (NRF11.3) | per 100kcal and per portion size | NRF index (139, 167).  Evaluation of methods (167). | [encourage] Protein, fiber, vitamin A, vitamin C, vitamin E, vitamin D, calcium, iron,  magnesium, potassium, folate  [limit] saturated fats, added sugar, and sodium |  | The nutrient density of the included food products was calculated using different variants of the Nutrient Rich Foods (NRF) index. NRF11.3 assigns a nutrient density score based on 11 nutrients to encourage and 3 nutrients to discourage using the below equation:  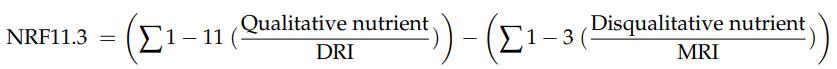   - DRI = A mean of sex and age specific dietary reference intakes - MRI = Maximum recommended intakes.   Both as per the NNR. |
| **Takacs et al. (2025) [141]** | Nutrient Rich Food Index (NRF 9.3 and 17.3) | Per portion | Previously published methodology [150, 151]. | [Encourage (n=9)] : protein,  fibre, vitamin A, vitamin E, vitamin C, calcium, magnesium, iron, and potassium  [Encourage (n=17)]: protein, fibre, seven vitamins (A, B1, B2, B9, B12, C,  and E) and eight minerals (K, Ca, Mg, Fe, I, Cu, Zn, and Se)  [Limit (n=3)] : SFA, addedd sugar and sodium |  | The NRF n.3 scores were calculated for each recipe as the sum of values of the n qualifying nutrients to encourage minus the sum of values of the three nutrients to limit. |
| **World Index for Sustainability and Health (WISH)**  **[142]** | EAT-Lancet Report Recommendations | g per day. | EAT-Lancet report (147, 148). |  | [protective] whole grains, vegetables, fruits, dairy, fish, legumes, nuts and unsaturated oils.  [neutral] eggs, chicken and other poultry  [limit] red meat, saturated oils and added sugars. | A score of 0-10 was assigned for all 13 food groups. 0 indicates no adherence to our set of reference recommendations and 10 indicates complete adherence.  For protective food groups, “Assuming a linear relationship between the component and the health outcomes, for a consumption between the lower recommended intake and the recommended intake, a score between 0 and 10 is assigned according to the following formula”:  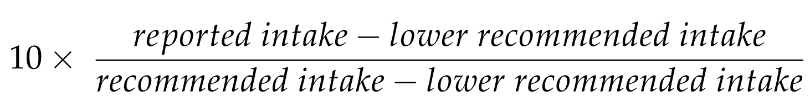  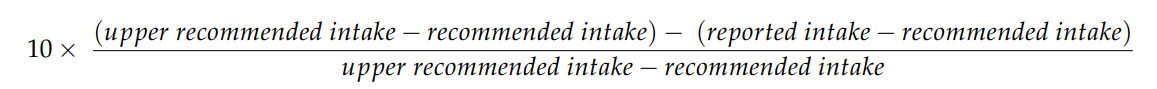For neutral and limiting food groups, the score is calculated as per the below equation:  Two sub-scores were created to assess diet quality.   - “Healthy sub-score: Summing the 8 protective and 2 neutral food groups: whole grains, vegetables, fruits, dairy, fish, eggs, chicken and other poultry, legumes, nuts and unsaturated oils. A higher sub-score for protective foods means a higher adherence to the recommendations for these protective foods and thus a healthier diet.”   “Less healthy sub-score: Summing the 3 limiting food groups: red meat, saturated oils and added sugars. A higher sub-score for limiting foods means a higher adherence to the recommendations for these limiting foods and thus a healthier diet.” |
| **van Dooren et al. (2014)**  **[143]** | Health Score | Vegetables, fruit, fiber, fish, salt = g per diet.  total, trans, SFA, free sugars = % energy of diet.  Energy balance = kcal of diet. | Based on the Healthy Eating Index (161, 162) using Dutch Health Council recommendations (163). | [limit] total fatty acids, saturated fat, trans fats, (free) sugars, salt (sodium chloride), energy. | [encouraged] vegetables, fruits, (fatty) fish, fiber. | 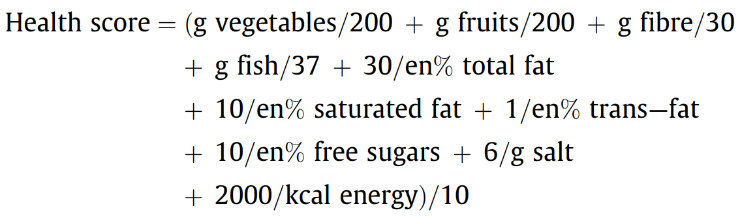The Health Score was calculated based on ten nutritional indicators and using the following formula: |
| **The Nutrient Density of Climate Impact (NDCI) index**  **[144]** | Nutritional value & nutrient density | Per 100g | Nutritional value & nutritional density (140). | protein, carbohydrates, fat, vitamin A, vitamin D, vitamin E, vitamin C, vitamin B12, niacin, thiamin, riboflavin, vitamin B6, folate, magnesium, iron, zinc, phosphorus, potassium, calcium, selenium, iodine. |  | Nutritional value was calculated as the nutrient composition for each food type using Dankost 3,000 dietary assessment software for the 21 nutrients included, as specified by the NNR.  Nutritional density was calculated by summarising the proportions of the recommended daily intake of each nutrient provided by 100 g of the food item multiplied by the proportion of nutrients contributing to more than 15% NNR (as per the below equation):  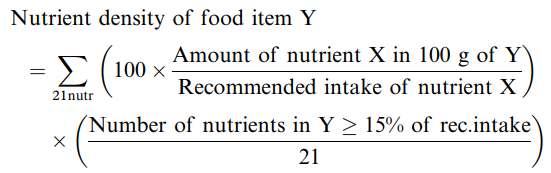 |

*Publication citation used where no index name is available.

# Supplemental Table 7: The environmental scoring method, environmental indicators included in the score, and an overview of the methodology used to quantify the environmental dimension of food-based indices for the assessment of nutritive value and environmental impact of meals and diets.

|  | **Environmental Scoring Method** | | | **Environmental Impacts Included** | | **Methodology Outline** |
| --- | --- | --- | --- | --- | --- | --- |
| **Index Name*** | **Name** | **FU** | **Validation** | **Environmental impact indicators** | **System Boundaries** |  |
| **Environmental Impact Weighted Daily Value score (EIWDVs) model**  **[120]** | LCA^†^ | Per L or kg | Previous methodology referenced [152]. | Ecotoxicity, fossil fuel depletion, global warming, smog, eutrophication, acidification, respiratory effects, ozone depletion, non-carcinogens, carcinogens. | Cradle-to-gate. | LCA followed the following four-stepwise procedure:   1. Goal and scope definition 2. Life cycle inventory 3. Life cycle impact assessment (TRACI 2.1 assessment method was considered) 4. Interpretation of results as explicated by the ISO 2006: 14040/44 standard. |
| **Sustainability Score**  **[121]** | LCA | Per kg food. | Previous methodology referenced [153]. | GHGe^‡^, LU^¶‡^, eutrophication potential, water withdrawal. | Not clearly stated. | No further details given beyond databases used. |
| **Batlle-Bayer et al. (2019)**  **[122]** | LCA | Food basket that contains the daily amount of the representative food products, consumed by an adult, which supplies the daily required amount of energy and nutrients  Per day | LCA data and assumptions when carrying out LCA referenced [154]. | GHGe. | Primary production to consumption (cradle-to-plate). | No further details clearly stated. |
| **Batlle-Bayer et al. (2020)**  **[123]** | LCA | Annual food basket consumed by a Spanish citizen that supplies the required energy and nutrients intake and is affordable. | Built on previous methodology [154]. | GHGe, blue water footprint (BWF) and LU. | Cradle-to-grave. | A life cycle inventory approach was taken where the countries of origin and their contribution to the national supply of each product was determined and an extensive search on input data and environmental outputs for all combinations of food products and countries was performed. |
| **Cooreman-Algoed et al. (2020) [124]** | LCA | Per hot served meal. | Validated databases used- eocinvent, agri-footprint and LCA Food DK and a validated impact assessment method-ReCiPE.  Ranking of ReCiPe values referenced [155]. | GHGe. | Cradle-to-plate. | ReCiPe 2008 Endpoint and single score (H) 1.12 with Europe ReCiPe H/A normalization/weighting and the IPCC 2013 GWP 100a for the environmental assessment was used. Monte Carlo simulations were performed for the environmental assessment in SimaPro.  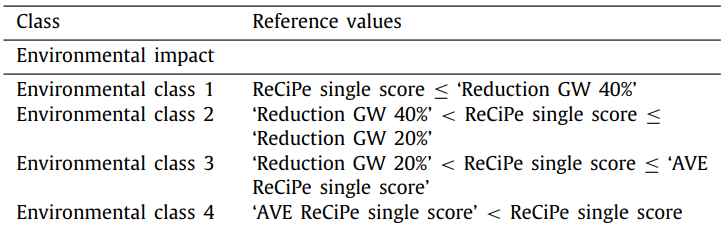Values were ranked from class 1-class 4 based on ReCiPe single score, as per below. |
| **Healthy and sustainable preparation index (HSPI)**  **[125]** | Water, carbon and ecological footprint | Per serving weight. | Not clearly stated for methodology.  Values used were referenced [156]. | WF, CF, EF. | Not clearly stated. | The calculation of water, carbon and ecological footprints was performed using the equation below:  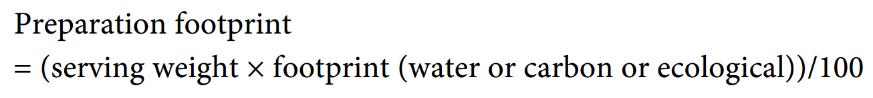 |
| **Dourmad et al. (2019)**  **[126]** | LCA | Per kg food. | Previous methodology referenced [157]. | Climate change, cumulative energy demand, acidification, eutrophication, land occupation and global impact. | Cradle-to-plate. | LCA using a methodology developed by as part of the AGRALID project. |
| **[new] sustainable diet index (SDI)**  **[127]** | Environmental Impact Index (Fresan 2017) | per kg | Previously described methodology used [158]. | Resource use land, water and energy, GHGe. | Agricultural production and food processing  (cradle-to-gate). | For each environmental impact indicator, values were divided into quartiles to rank them from 1 (lowest resource consumption or GHG emission) to 4 (highest resource consumption or GHG emission).   - Quartile 4 = 0 points - Quartile 3 = 1 point - Quartile 2 = 2 points - Quartile 1 = 3 points   The environmental impact index is the sum of the quartiles for each of the four footprint values. |
| **Performance-weighted environmental sustainability (PwES) [128]** | Share of Safe Operating Space (SoSOS) | Per kg per year | PwES previous method referenced [145]. Questions used referenced [159]. | Land use, freshwater use, climate change – GHGe. | cradle-to-comsumption for freshwater use and climate change.   Agriculture only for LU. | Environmental impact indicators were calculated into share of the safe operating space (SoSOS) using the below equation.  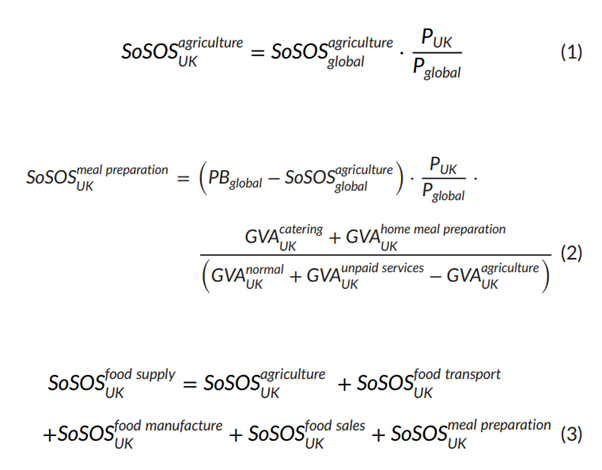 |
| **The Food-Triad [129]** | Environmental Dimension | Not clearly stated. | Previous methodology referenced [160]. | water use, contamination, liquid effluents, solid waste, atmospheric emissions, power use, industrial packaging. | From the raw material in *natura* to the final consumer  (cradle-to-plate). | Only the presence/absence of an environmental impact of an industrialized link from the food chain has been considered.  The following scoring system is implemented:   - 1 point if the environmental impact is present - 0 points if the environmental impact is not present.     The calculation of the environmental dimension is as follows:  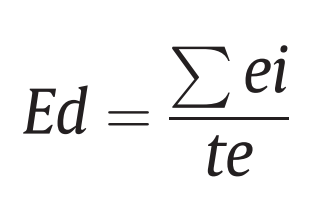  Where e*i* is the presence of an environmental impact, and *te* the number of total impacts evaluated along the chain.  For each impact evaluated the closer to 1 the higher the impact. |
| **Eco-nutritional-efficiency (ENE) [130]** | LCA | per kg of food | Existing methodology referenced [161, 162]. | Carbon footprint. | Primary agricultural production, processing, transportation, packaging and cooking (LCA for each product may have different system boundaries based on data availability).  (cradle-to-plate). | For calculating the carbon footprint potential (CFP) of the daily menus, the preliminary CFP data per kg of food product were multiplied by menu composition.  To reach a geographical specificity where literature from other countries was used, the MEXALCA method for the extrapolation of crop LCA (life cycle assessment) was applied. |
| **Sustainability Index**  **[131]** | Not clearly stated. | Per 100kcals | Environmental impact data referenced [163].  Referencing for methodology not clearly stated. | LU, GHGe, acidifying emissions, eutrophying emissions and stress-weighted water use. | Input (initial effect of producer choice) to retail (cradle-to-shelf). | A composite score for the environmental impacts was calculated such that each of the factors received an equal weighting.  The value for each environmental indicator was normalised using min-max scaling from 0-1. The average of the normalised impact factors was then scaled up by a factor of 100, where 100 represents the highest impact within each factor. |
| **Food consumption sustainability index [132]** | Ecological Effect | Per capita | Each nutritional quality indicator used was referenced [146, 153, 164-167]. | Per capita FCF (food consumption footprint) (gha)  Per capita GHGe (t CO2e)  Equivalent ecosystem service values (100 million yuan)  Per captia FCF intensity (gha/yuan) | Cradle to plate | 1. Per capita FCF (food consumption footprint) = calculated according to formula shown below (full equation explanation is given in the paper).   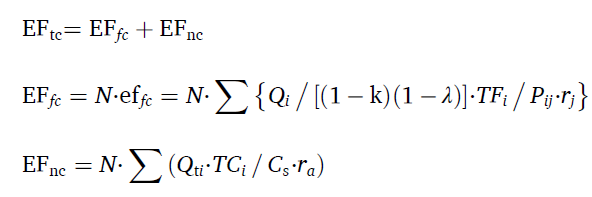  EFtc (total footprint) = direct consumption footprint + indirect (embedded) footprint; both are calculated as population × the per-person sum across food types, where per-person terms account for consumption, waste, land conversion/productivity and carbon-to-land conversions.   1. Per capita GHG emissions = Calculated by emission factor method. 2. Equivalent ecosystem service values = Calculated by equivalent factor method. 3. Per captia FCF intensity = Per capita FCF / Per capita disposable income. |
| **Comprehensive assessment index (CAI)**  **[133]** | LCA^*^ | per unit of food. | LCA and system boundary selection referenced [168]. | Carbon footprint, water footprint, ecological footprint, food waste rate. | Farm-to-waste (cradle-to-grave). | Value calculated for each environmental footprint indicator (carbon, water, ecological) by summing each footprint coefficient of the food at the following stages: production, transportation, storage, retail, consumption and waste treatment of food supply chain (see below equations):  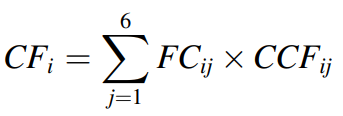   1. Carbon footprint   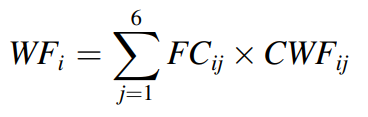CF*i* is the carbon footprint of food *i*; CCF*ij* is the carbon footprint coefficient of food *i* at stage *j*. which stands for the amount of carbon emission per unit of food *i* in stage *j*.   1. Water footprint   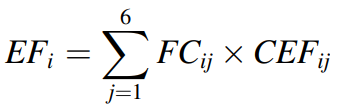Where WF*i* is the water footprint of food *i*; CWF*ij* is the water footprint coefficient of food *i* at stage *j* which stands for the amount of water footprint per unit of food *i* in stage *j*.   1. Ecological footprint   Where EF*i* is the ecological footprint of food *i*; CEF*ij* is the ecological footprint coefficient of food *i* at stage *j* which stands for the amount of ecological footprint per unit of food *i* in stage *j*.  Food waste rate is the proportion of all foods consumed by households that were wasted (as per the equation below).  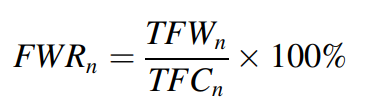  Where FWR*n* is the food waste rate of household *n*; TFC*n* is the total food consumption of household *n*, TFW*n* is total food waste of household *n*. |
| **Nutritional Footprint**  **[134]** | Environmental Indicators (nutritional footprint environment) | Per day per person and per meal. | Threshold levels referenced [169-175]. | Material footprint, carbon footprint, water footprint,  LU. | Not clearly stated. | A value of 1 (small impact) to 3 (strong impact) was assigned to each environmental indicator according to the below thresholds. Per cap/day:  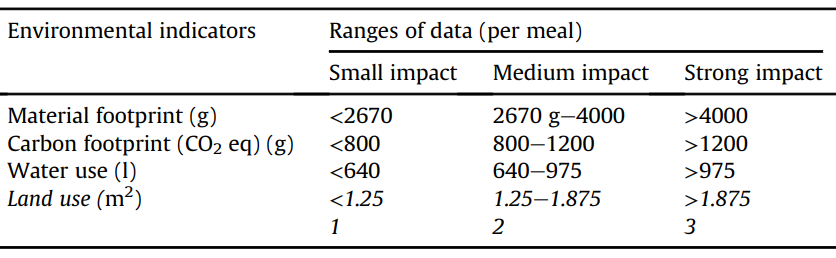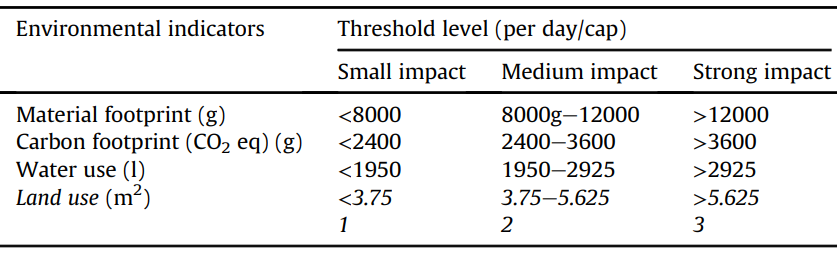Per meal:  The environmental indicator is calculated by summing the environmental impact indicator score over the number of indicators (as per the equation below).  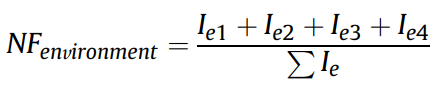 |
| **Planet Health Conformity Index (PHC) [135]** | Not clearly stated | Per 100g of product. | Source of absolute values referenced [176]. | GHGe, cropland use, freshwater use, mineral nitrogen application and mineral phosphorus application. | Cradle-to-farmgate | Absolute values used. |
| **Rӧӧs et al. (2015) – Method one and Method two**  **[136]** | Life Cycle Assessment (LCA). | Per kg food eaten.  per kg of food. | Impact categories and values for each were referenced [172, 177].  Method to assess biodiversity referenced [178]. | Climate change, LU^†^, biodiversity damage potential (BDP). | Cradle-to-plate. | LCA.  LCA results for climate change and LU were normalized according to sustainable levels.   - Sustainable levels for greenhouse gas emissions were set at 750 kg CO2e per person per year. - The sustainable level of land occupation was defined as 0.32 ha per capita and year.   No sustainable per capita limit has been defined for BDP. |
| **Schaubroeck et al. (2018)**  **[137]** | LCA | per served portion. | Previous methodology referenced [179] [180, 181]. | Ecological footprint (LU and water consumption) and carbon footprint. | Cradle-to-shelf/canteen. | Ecological footprint is defined as the land occupied over time (m^2^*year) and was combined with freshwater depletion to also address water consumption.  An ecological score was given from 1-8 based on the ecological footprint, the amount of land occupied over time (as per the table below).  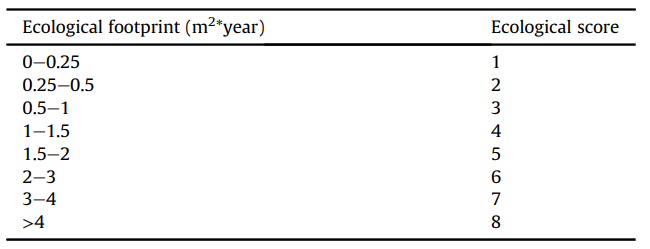  The results of the carbon footprint method were also considered. Carbon footprint was calculated using the carbon footprint method, which expresses impact in kg CO2-equivalents. |
| **Sustainable diet index (SDI) [138]** | LCA | per year | Prior methodology referenced [92]. | GHGe^‡^, primary energy consumption, land occupation, contribution of organic food to diet. | Raw agricultural products to consumer use (cradle-to-plate). | Environmental impact of diet (GHGe, energy consumption and land occupation) was calculated using the Partial ReCiPe (pReCiPe) score calculated as follows:  *pReCiPe = (0·0459×greenhouse gas emissions (in kg CO2eq/kg))+ (0·0025×primary energy consumption (in MJ/kg)) + (0·0439×land occupation (in m2 /kg))*  pReCiPe was multiplied by the quantity of consumed food (g/d), accounting for the method of agricultural production.  Share of organic food in the diet was used as a proxy for biodiversity preservation in farm.  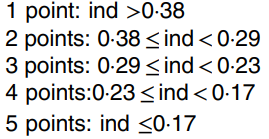Points were then allocated (1-5) based on outcome value for:   1. pReCiPe (weight = ¾)   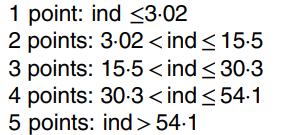   1. Mitigation of biodiversity loss in farm (weight = ¼)   Final environmental impact assessment was calculated as follows:  $Environmental subindex=the sum of points x weight$ |
| **Dietary dependent nutrient quality index (NQI)**  **[139]** | LCA | Per kg food. | Previous methodology referenced [182]. | Global Warming Potential. | Not clearly stated. | No further details clearly stated. |
| **Parallel/integrated climate-nutrient score**  **[140]** | Climate Impact (based on LCA) | Per kg | Not clearly stated. | GHGe (CO2e). | Primary production to raw food products possible processing in industry (excluding packaging and emissions from land use change) (cradle-to-farm gate/plant gate). | Greenhouse gas emissions for all food products estimated data based on LCA from RISE Food Climate Database. |
| **Takacs et al. (2025) [141]** | LCA | Per portion. | Previous methodology referenced [183, 184]. | Global warming potential (GWP), freshwater eutrophication potential (FEP), terrestrial  acidification potential (TAP), and water depletion potential (WDP) | Cradle-to-plate | Methodology (ISO 14040 and ISO  14044). |
| **World Index for Sustainability and Health (WISH) [142]** | Not clearly stated. | N/A | Based on previous study classification of environmental impact of food groups [185]. | Generic environmental impact  GHGe, LU, eutrophication, acidification, scarcity weighted water | Not clearly stated. | Environmental impact of food classified as low, medium or high environmental impact.  For all 13 food groups a score of 0-10 was assigned for both healthiness (protective, neutral and limit) and environmental impact (low, medium and high). 0 indicates no adherence to our set of reference recommendations and 10 indicates complete adherence. |
| **van Dooren et al. (2014)**  **[143]** | Sustainability Score (based on LCA) | Per day. | Methodology framework referenced [186]. | GHGe, LU. | Cradle-to-gate | Th agri-footprint method; methodological framework was used for GHG and LU and calculations were conducted according to the British PAS2050 specification [187].  The sustainability score was defined as the average of the GHG and LU score per diet and calculated using the equation below:  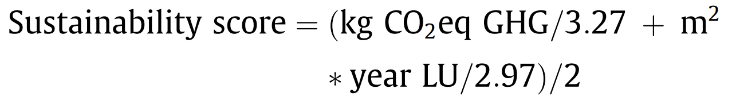  A score of 100 complies with a 20% reduction in GHG (or 3.75kg CO_2_eq/day) and a 44% reduction in LU (from 5.34 m^2^ year/day (the land use of the average Dutch diet to 2.97 m^2^ year/day) (2020 European Commission goal). |
| **The Nutrient Density of Climate Impact (NDCI) index**  **[144]** | Carbon Footprint | per 100 g food item | Not clearly stated. | GHGe. | Primary production to final consumption  (cradle-to-plate). | The total estimated greenhouse gas emission (in g CO2e per day). |

^*^Publication citation used where no index name is available.
^†^Life Cycle Assessment.
^‡^Greenhouse Gas emissions.
^¶^Land Use.

# Supplemental Table 8. Overview of criterion and scoring/ranking of food-based indices for the assessment of nutritive value and environmental impact of meals and diets.

| **Index Name*** | **Index Creation** | | **Index Scoring / Ranking** | | **Validation** |
| --- | --- | --- | --- | --- | --- |
|  | **Methodology outline** | **Weighting** | **Methodology Outline** | **Relative or Absolute** |  |
| **Environmental Impact Weighted Daily Value score (EIWDVs) model**  **[120]** | The EIWDVs is calculated as the ratio of the result for a specific impact category (EIs) (either from the primary LCA results or secondary data) to the WDVs (see equation below).  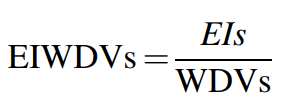 | Not clearly stated. | A lower EIWDV showed an increased compensation of environmental impact via satisfaction of required daily values of nutrients. | Absolute | Validation for each dimension shown in supplementary material. |
| **Sustainability Score**  **[121]** | Two scores were created   1. Total sustainability score 2. Mean sustainability score   Data was converted to values per 1-gram serving and then multiplied by the number of grams recommended for all the servings within that die to give the  total impact for a given diet  The total result of each metric was divided by the number of grams consumed in that diet to give the mean value per gram.  To obtain relative sustainability scores, points were assigned based on the comparisons of the total and mean of individual diet metrics to the overall total average and overall mean average across all diets.  Diets receive one point each if:   - Environmental indicators are lower than the overall metric average across diets - SAIN:LIM is higher than the overall metric average - price is lower than the overall metric average   Diets receive zero points each if:   - environmental indicators are higher than the overall metric average across diets - SAIN:LIM if lower than the overall metric average - price if higher than the overall metric average   Scores were then summed for each diet to give a total and a mean sustainability score. | Equal weighting to the following 6 metrics:   1. GHGe^†^ 2. Land use 3. Eutrophication potential 4. Water withdrawals 5. SAIN:LIM ratio 6. Price of meals for 7 days   Weighting:   - Nutrition: 0.166 ˙ - Environmental impact: 0.66 ˙ - Affordability: 0.166 ˙ | Two sustainability scores, one for the mean and one for the total, were calculated for each diet, with a maximum of 6 points indicating high sustainability and a minimum of 0 points indicating low sustainability.   - 0-2 = not sustainable for at least one metric. - 3-4 = environmentally friendly but may not be good for other metrics. - 5 = high sustainability diet.   6 = diet is sustainable across all metrics and represents FAO's definition of a sustainable diet. | Relative | Based on previous literature [153].  Scoring based off FAO’s definitions of a sustainable diets [188]. |
| **Batlle-Bayer et al. (2019)**  **[122]** | GHGe are corrected by the energy and nutritional scores so that they fulfil the requirements defined in the FU (a caloric and nutrient corrected FU).  The equation below is used:  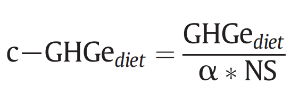   - α = the energy score (if diets have a lower caloric intake than recommended). - α = the inverse of the energy score (if the diets have a higher caloric intake than recommended) - NS = nutritional score   FU of the diet was defined as the food basket that contains the daily amount of the representative food products, consumed by an adult, which supplies the daily required amount of energy and nutrients. | Not clearly stated. | Not clearly stated. | Absolute | Previous studies used as basis for methodology [95, 105, 107, 189, 190]. |
| **Batlle-Bayer et al. (2020)**  **[123]** | Nutritional quality (required energy and nutrient intake) and affordability used as a functional unit of LCA (alongside residual income).  The environmental impacts (EI) resulting from the food basket were corrected by nutrition and economic attributes using the below equation:  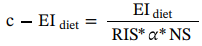   - EI diet = environmental impacts of the diet. - RIS = Residual income representing food affordability. - α = energy intake   NS = The nutritional score | Not clearly stated. | Not clearly stated. | Absolute | Reference to a previously used methodology [122]. |
| **Cooreman-Algoed et al. (2020)**  **[124]** | No clear mention of methodology beyond individual dimension methodology. | Not applicable for individual dimension scoring indices. | Nutritional class 1 and environmental class 1 represent meals that are ‘good’ for nutrition and environmental sustainability.  Nutritional class 4 and environmental class 4 represent meals that are ‘bad’ for nutrition and environmental sustainability.  Classes 2 and 3 represent meals that are ‘intermediate’ for nutrition and environmental sustainability. | Relative | Validation for each dimension shown in appendix S6 and S7. |
| **Healthy and sustainable preparation index (HSPI)**  **[125]** | 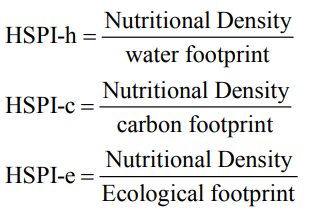The nutritional density was divided by each environmental indicator to give HSPI-h, HSPI-c and HSPI-e (equations shown below). | - Nutrition: 0.5 - Environment: 0.5   Not clearly stated. | The preparations that presented the highest HSPI-h, HSPI-c, HSPI-e were considered the best options because they presented a better combination of nutritional profile and lower environmental impact. | Absolute | Combination methodology based on Semdman *et al*. 2010 [191]. |
| **Dourmad et al. (2019)**  **[126]** | Nutritive value and environmental impact value presented without further methodology. | Not applicable for individual dimension scoring indices. | Values conveyed as a percentage of set recommendations for each dimension.  Scores of 100 for both dimensions comply with set recommendations. | Absolute | Validation for each dimension shown in appendix S6 and S7. |
| **[new] sustainable diet index (SDI)**  **[127]** | Scores for all sub-indices were divided into quartiles (Q) as per the below   - nutritional quality index   - Q1 = 0 points (least healthy)   - Q4 = 3 points (healthiest) - Environmental impact   - Q1 = 3 points (most environment-friendly)   - Q4 = 0 points (least environment-friendly) - Market price   - Q1 = 3 points (least expensive)   - Q4 = 0 points (most expensive)   Quartiles 2 and 3 were given scores relative to the assigned scores for quartiles 1 and 4 in each component.  The [new] SDI is the sum of all sub index scores (nutritive, environmental and market price). | - Nutritional quality: 0.33˙ - Environment: 0.33˙   Market price: 0.33˙ | The SDI scores ranged from 0 (diet with the lowest sustainability) to 9 (the most sustainable diet). | Relative | Reference to a previously used similar methodology [192]. |
| **Performance-weighted environmental sustainability (PwES) [128]** | Environmental impact weighted by their nutritional content, which is then normalized with the share of Safe Operating Space (SoSOS) and a functional unit from demand for nutrition is derived.  The PwES calculation is performed using the below equation:  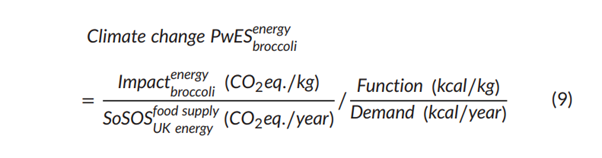  (the mean GHG emission impact of the energy of steamed broccoli was used as example). | Environmental impact weighted by their nutritional content. | Results are unitless.  Values greater than 100% are considered unsustainable with respect to impact category under assessment. | Absolute | Previous method for PwES referenced [145] |
| **The Food-Triad**  **[129]** | Values for nutritional, environmental and health dimensions were plotted on a graph and the generated area within the triangle is the ‘food-triad’ and is the value used to determine the level of sustainability. | - Nutritional: 0.33˙ - Environmental: 0.33˙ - Health: 0.33˙ | The value range is from 0 to 1.   - 1.3u^2^ (the maximum area possible, with 1 in all dimensions) = less sustainable. - 0.01u^2^ (the most minimum area possible, with 0.1 in all dimensions) = sustainable   (u is the unit of measurement). | Absolute | Previous method referenced [193, 194]. |
| **Eco-nutritional-efficiency (ENE)**  **[130]** | Data Envelopment Analysis (DEA) is used to aggregate the multiple nutrition value outputs, with the CCR-model 21 assuming that the relationship between nutritional contents and carbon footprint is constant. | Subjective weighting is avoided. | The resulting ENE lies between 0-1 with 1 representing an efficient menu. | Absolute^‡^ | Use of DEA [195, 196]. |
| **Sustainability Index**  **[131]** | A composite score for each of the four sustainability indicators was calculated. These values were normalized via mix-max scaling so that all values ranged from 0-1, with 1 representing the highest impact within each factor.  Scores were then summed. | - Nutrient density: 0.25 - Environmental impact: 0.25 - Monetary cost: 0.25 - Social and cultural acceptability: 0.25 | Foods and beverages received one point for being above the food group-specific median for nutrient density and frequency and below the median for cost and environmental impact. Otherwise, the items received a zero for that indicator.  Scores ranged from 0-4.  4 = Foods groups or beverages that were optimally sustainable. | Relative | Following previous methods  [1, 153]. |
| **Food consumption sustainability index [132]** | The original data were normalised using the Min-Max Normalization method. The equation for the food consumption sustainability method is shown in the below equation (full equation explanation is given in the paper).:  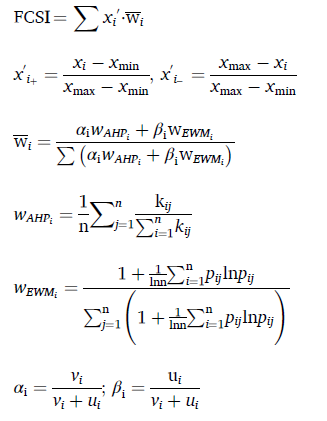 | - Nutritional weighting: 0.2296 - Environmental weighting: 0.3222 - Economic benefit: 0.2048 - Social influence: 0.2435 | Ranking from 0-1.  A city’s food-consumption footprint is within an external ecological threshold if it's less than 1.  If the value is above 1 then a city’s food-consumption footprint exceeds an external ecological threshold. | Absolute | Based on the EESN framework (no reference given) and referenced previous assessment indicators [133, 197]. |
| **Comprehensive assessment index (CAI)**  **[133]** | Each assessment indicator score (nutritive, environmental, economic & socio-cultural) were standardized on a scale of 0-100 points.  The assessment dimension indicator score (CAI score = CAISC) was calculated based on its calculated assessment indicator scores and the corresponding weights  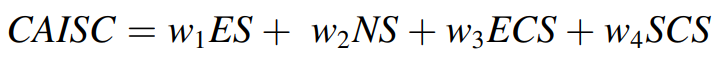using the below equation:   - Es: environmental dimension - Ns: nutritional dimension - Ecs economic dimension - SCs: socio-cultural dimension | - Nutrition: 0.3 - Environmental: 0.26 - Economy: 0.25 - Socio-culture:0.19 | Scoring can be for each assessment indicator or combined:   - High sustainability / green zone (75-100 points) - Yellow zone - (50-75 points) - Orange Zone (25-50 points)   Low sustainability / red zone (25-50 points) | Absolute | Standardization approach based on Gustafson et al. (2016) [52].  Weighting determined by surveyed experts [133].  Scoring based on previously reported work [198, 199]. |
| **Nutritional Footprint**  **[134]** | 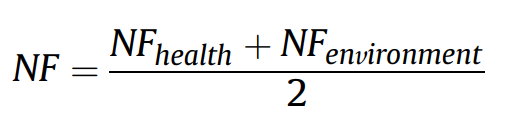Both effect levels are summed up and the average is determined as per the equation below: | - Health indicator: 0.5 - Environmental indicator: 0.5 | The results are transferred into qualitative effect levels:   - Low effect = 1–1.6 - Medium effect = 1.6–2.2. - High effect = 2.2-3.0   Low effect level is recommended, without restrictions.  High effect is recommended once or twice a week. | Absolute | Qualitative effect levels and recommendations are inspired by German recommendation levels (no reference clearly stated). |
| **Planet Health Conformity Index (PHC) [135]** | For each nutrient, the environmental impact of a product was divided by its nutrient content. This ratio was then normalised by dividing it by the ratio of the recommended daily planetary allowance for that environmental impact to the recommended daily nutrient intake. The resulting values for all nutrients were then aggregated - either by calculating their arithmetic mean or median, with or without weighting - to produce the overall Planetary Health Contribution (PHC) Index. See below equations:  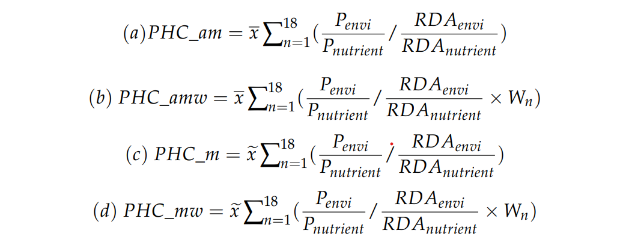 | Equal weighting assumed  N.B. Different weighting factors for each nutrient included | Boundary Transgressions were created - Nutrient-related Planetary Boundaries (NuPlaBos) for each environmental indicator.  A PHC factor of 1  or lower ensures that the planetary boundary of an environmental impact factor is preserved when consuming a product while meeting the recommended daily requirements RDA  of a nutrient.   - A = <0.5 - B = <1 - C = between 1 and <2 - D = between 2 and <4 - E = more than 4x the - corresponding NuPlaBo transgression | Absolute | Design of the index followed previously published “points-of-differentiation” steps [200] and compared PHC with other indices. |
| **Rӧӧs et al. (2015) – Method one**  **[136]** | Environmental impact data were normalized according to estimated sustainable levels as per below for each environmental impact indicator.   - GHGe: 750 kg CO2e per person and year - land occupation was defined as Swedish agricultural land availability in 2013 (0.32 ha per capita and year, based on Swedish population in 2014) (SBA, 2013; SS, 2014)   It was not possible to normalise BDP, since no sustainable per capita limit has been defined for BDP (Tuomisto et al., 2012) [201].  Nutrient intake data were normalised but methodology for this is not clearly stated.  Normalized environmental impact and nutritional data were presented in parallel as two separate values. | Not applicable for individual dimension scoring indices. | Value of 1 for normalized environmental impacts represents per capita sustainable level of climate impact and agricultural land occupation and a value of 1 for normalized nutrient intake corresponds to the recommended intake according to the Nordic Nutrition Recommendations. | Relative | Sustainable levels for environmental impacts based on IPCC (2013) [202], SBA, (2012) [203] and SS, 2014) [204].  Recommended intakes for nutrients based on NNR[205]. |
| **Rӧӧs et al. (2015) – Method two**  **[136]** | Methodology for calculating the environmental impact per nutrient density score is not clearly stated. | Not clearly stated. | Climate impact of diets is presented in relation to the SNӦ diet (100%) which is the cut off for an environmentally and nutritionally sustainable diet.  The SNӦ diet is a diet corresponding to the NNR and reflects food preferences in Sweden [206]. | Relative |  |
| **Schaubroeck et al. (2018)**  **[137]** | Nutritional score and ecological value presented without further methodology. | Not applicable for individual dimension scoring indices. | Lower scores are better for each dimension (range 1-8). | Relative | Validation for each dimension shown in appendix S6 and S7. |
| **Sustainable diet index (SDI)**  **[138]** | Each sub-index dimension (n=4) was scored from 1-5 using population quintile values as cut-offs. The following  calculation was used to give each sub-index a final value:  $Sub index=the sum of points x weight$  The value of the four sub-indices were summed and then divided by the number of sub-indices included. | Index weighting: equal weighting between n=4 sub-indices.   - nutritional: 0.25 - environmental: 0.25 - Economic: 0.25 - Sociocultural: 0.25   Indicators in sub-index weighting:  *Nutritional:*   - Energy: 0.5 - PANDiet index: 0.5   *Environmental:*   - ReCiPe: 0.75 - Organic food contribution: 0.15   *Economic:*   - % income to diet: 1   *Socio-cultural:*   - Place of food purchase: 0.5 - Read-made products: 0.5 | - 1 point assigned if indicators considered ‘valuable’ for sustainability were in the first quartile. - 2 points assigned if indicators considered ‘valuable’ for sustainability were in the second quartile. - 3 points assigned if indicators considered ‘valuable’ for sustainability were in the third quartile. - 4 points assigned if indicators considered ‘valuable’ for sustainability were in the fourth quartile.   For indicators presumed to be ‘damaging’ to sustainability, the allocations were reversed.  The score ranged from 4-20.  A higher score represents a more nutritionally adequate and environmentally friendly diet. | Relative | External sustainable guidelines were used to investigate the SDI construct validity [207, 208]. |
| **Dietary dependent nutrient quality index (NQI)**  **[139]** | The NQI was used as a complementary FU in an LCA to give the climate impact per kg of food. | Not clearly stated. | The lower the NQI-adjusted GWP/kg the more nutritional value present in that dietary context with a lower environmental impact. | Absolute | Not clearly stated. |
| **Parallel climate-nutrient score**  **[140]** | Relative nutrient density was plotted on one axis and relative climate impact on the other.  Food subgroups were evaluated in relation to the median performance of all food subgroups and expressed as a percent of the median. | Equal weighting assumed. | The assessment was based on foods with the best and worst performance.  Graph split into quadrants. The best performing foods represented by food products in the higher nutrient density and lower climate impact quadrant. With the worst performing in the lower nutrient density and lower climate impact quadrant.  Food subgroups would then be labelled “green” (increase consumption of), “yellow” (healthier options that unhealthier ones should be exchanged to) and “red” (limit). | Relative | Validation for each dimension shown in appendix S6 and S7. |
| **Integrated climate-nutrient score**  **[140]** | For the integrated climate-nutrient score, the ratio of climate impact to nutrient density was calculated by dividing kg CO2e per kg food product by NRF11.3 per reference unit of food product. | Equal weighting assumed. | The integrated score ranked the subgroups according to climate impact relative to nutrient density.  The assessment was based on foods with the best and worst performance.  Q1 (best performing) and Q5 (worst performing) ranked based on integrated climate-nutrient score.  Food subgroups would then be labelled “green” (increase consumption of), “yellow” (healthier options that unhealthier ones should be exchanged to) and “red” (limit). | Relative | Assessment of results based on previous method [209]. |
| **Takacs et al. (2025) [141]** | The results of each individual assessment were normalised separately using the min–max technique and summed.  Following normalisation, recipes were ranked relative to each other based on their  final environmental impact score, final nutritional quality score, and recipe cost score. The  final sustainability ranking integrated these normalised scores. | Equal weighting | The worst option for each attribute received a score of 0 and the best option received  a score of 1. The remaining options were assigned scores between 0 and 1, depending on  how they compared to the worst and best options. | Relative | Validation for each dimension shown in appendix S6 and S7. |
| **World Index for Sustainability and Health (WISH)**  **[142]** | For the calculation of the total WISH score, all components are summed up and are given equal weight in the total score.  Four sub-scores are also calculated to overcome a potential diluting effect.   - healthy sub-score - less healthy sub-score - low environmental impact sub-score   high environmental impact sub score | Equal weighting of the thirteen dietary components. | Maximum total score of 130.  Higher mean scores on the total and sub-scores indicate the population consumes a   - healthier and more sustainable diet. | Absolute | Validation for each dimension shown in appendix S6 and S7. |
| **van Dooren et al. (2014)**  **[143]** | Health score and sustainability score presented without further methodology. | Not applicable for individual dimension scoring indices. | Scores of 100 for both dimensions comply with set recommendations for each dimension. | Relative | Validation for each dimension shown in appendix S6 and S7. |
| **The Nutrient Density of Climate Impact (NDCI) index**  **[144]** | 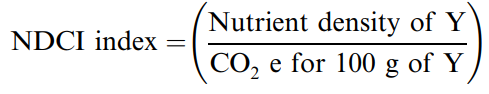The NDCI index is calculated as the nutrient density divided by the CO2e for 100 g of food items without including a cut-off value following the equation below: | Not clearly stated. | Food items with the highest NDCI index values had the highest nutrient density score in relation to GHGe. | Absolute | Validation for each dimension shown in appendix S6 and S7. |

*Publication citation used where no index name is available.
^†^Greenhouse gas emissions.
^‡^One approach used in the nutritional dimension was relative to recommended daily intake (RDI) of fractions of macronutrients, whereas a second approach used in the nutritional dimension was the absolute macronutrient content. Environmental scoring in the ENE was absolute.

# Supplemental Table 9: Index replicability assessment

| **Replicability** | **Description** | **Score** |
| --- | --- | --- |
| Replicable | Methods clearly explained (nutritional and environmental score used and encompassed indicators) with equations provided (including weighting), and all datasets are referenced and available. System boundaries and reference amounts are detailed and cut-offs or scoring of the final outcomes are provided. | 1 |
|  | Methods clearly explained (nutritional and environmental score used and encompassed indicators) with equations provided (or referenced). Data used are either posted online or type of data used are clearly described. System boundaries and reference amounts are detailed but cut-offs or scoring are not included in the main paper. | 2 |
|  | Method is clearly explained (nutritional and environmental score used and encompassed indicators) without equations provided, and a link to the database or original paper that contains data used in the present analysis is available. System boundaries or reference amounts may be detailed but cut-offs or scoring are not included in the main paper. | 3 |
| Possible replicable | Methods less clearly detailed (equations for nutritional score or environmental score not included, or information around system boundaries, reference amounts or weighting not detailed).  Methods and datasets are described such that the missing information or code could likely be found or recreated. | 4 |
| Probably not replicable | Method is not well-described, but there are enough results in the text (or available in a supplement) that the method could be recreated through trial and error. | 5 |
|  | Method is not well described, and limited results are available to recreate the method through trial and error. | 6 |
| Not replicable | Anything else | 7 |

# References:

1. Aceves-Martins M, Bates RL, Craig LCA, Chalmers N, Horgan G, Boskamp B, et al. Food-Level Analysis to Identify Dietary Choices With the Highest Nutritional Quality and Lowest Greenhouse Gas Emissions and Price. Frontiers in Nutrition. 2022;9. doi: 10.3389/fnut.2022.851826.

2. Agustina R, Febriyanti E, Putri M, Martineta M, Hardiany NS, Mustikawati DE, et al. Development and preliminary validity of an Indonesian mobile application for a balanced and sustainable diet for obesity management. BMC Public Health. 2022;22(1). doi: 10.1186/s12889-022-13579-x.

3. Agyemang P, Kwofie EM, Aidoo R, Allotey DK, Ngadi M. A multi-dimensional assessment of sustainable foods and the influence of stakeholder perceptions during nutrition interventions. Food Policy. 2023;118. doi: ARTN 102475

10.1016/j.foodpol.2023.102475. PubMed PMID: WOS:001024651300001.

4. Aidoo R, Abe-Inge V, Kwofie EM, Baum JI, Kubow S. Sustainable healthy diet modeling for a plant-based dietary transitioning in the United States. npj Science of Food. 2023;7(1). doi: 10.1038/s41538-023-00239-6.

5. Aldaya MM, Ibañez FC, Domínguez-Lacueva P, Murillo-Arbizu MT, Rubio-Varas M, Soret B, et al. Indicators and Recommendations for Assessing Sustainable Healthy Diets. Foods. 2021;10(5):999. doi: 10.3390/foods10050999.

6. Allen T, Prosperi P, Cogill B, Padilla M, Peri I. A Delphi Approach to Develop Sustainable Food System Metrics. Social Indicators Research. 2019;141(3):1307-39. doi: 10.1007/s11205-018-1865-8.

7. Angelsen A, Starke AD, Trattner C. Healthiness and environmental impact of dinner recipes vary widely across developed countries. Nature Food. 2023;4(5):407-15. doi: 10.1038/s43016-023-00746-5.

8. Aytekin-Sahin G, Besparmak A, Sagir SS, Somtas A, Ozturk D. Relationship between nutrient profiles, carbon footprint and water footprint of hospital menus. Nutrition &amp; Food Science. 2024;54(2):319-33. doi: 10.1108/nfs-07-2023-0154.

9. Azzini E, Maiani G, Turrini A, Intorre F, Lo Feudo G, Capone R, et al. The health‐nutrition dimension: a methodological approach to assess the nutritional sustainability of typical agro‐food products and the Mediterranean diet. Journal of the Science of Food and Agriculture. 2018;98(10):3684-705. doi: 10.1002/jsfa.8877.

10. Bälter K, Sjörs C, Sjölander A, Gardner C, Hedenus F, Tillander A. Is a diet low in greenhouse gas emissions a nutritious diet? – Analyses of self-selected diets in the LifeGene study. Archives of Public Health. 2017;75(1). doi: 10.1186/s13690-017-0185-9.

11. Barré T, Perignon M, Gazan R, Vieux F, Micard V, Amiot M-J, et al. Integrating nutrient bioavailability and co-production links when identifying sustainable diets: How low should we reduce meat consumption? PLoS One. 2018;13(2):e0191767. doi: 10.1371/journal.pone.0191767.

12. Baudry J, Neves F, Lairon D, Allès B, Langevin B, Brunin J, et al. Sustainability analysis of the Mediterranean diet: results from the French NutriNet-Santé study. Br J Nutr. 2023;130(12):2182-97. doi: 10.1017/s0007114523001411.

13. Benvenuti L, De Santis A, Cacchione P. Multi-indicator design and assessment of sustainable diet plans. Journal of Cleaner Production. 2021;313:127699. doi: 10.1016/j.jclepro.2021.127699.

14. Bianchi M, Strid A, Winkvist A, Johansson I, Sonesson U. Evaluating foods and diets from a multi-dimensional perspective: nutrition, health and environment. Proc Nutr Soc. 2020;79(OCE2). doi: 10.1017/s0029665120002840.

15. Bjornarå HB, Torstveit MK, Bere E. Healthy and sustainable diet and physical activity: the rationale for and experiences from developing a combined summary score. Scand J Public Health. 2019;47(5):583-91. doi: 10.1177/1403494818785056. PubMed PMID: WOS:000476525000015.

16. Broekema R, Blonk H. Determining the balance between nutrition and sustainability of 173 food products using stepwise optimisation. Proc Nutr Soc. 2020;79(OCE2). doi: 10.1017/s0029665120003730.

17. Bryan T, Hicks A, Barrett B, Middlecamp C. An Environmental Impact Calculator for 24-h Diet Recalls. Sustainability. 2019;11(23):6866. doi: 10.3390/su11236866.

18. Cáceres RP, Troncoso PC, Buhring BR, Lataste QC. Sustainable food dishes: Selection of indicators for their evaluation and communication in Chilean foodservices. International Journal of Gastronomy and Food Science. 2024;35. doi: ARTN 100873

10.1016/j.ijgfs.2024.100873. PubMed PMID: WOS:001169121600001.

19. Cambeses-Franco C, González-García S, Feijoo G, Moreira MT. Driving commitment to sustainable food policies within the framework of American and European dietary guidelines. Sci Total Environ. 2022;807. doi: ARTN 150894

10.1016/j.scitotenv.2021.150894. PubMed PMID: WOS:000711161700014.

20. Ceyhun Sezgin A, Eroğlu FE, Şanlıer N. Sürdürülebilir Beslenme Modellerinin Karşılaştırılması. Turkish Journal of Agriculture - Food Science and Technology. 2023;11(3):603-16. doi: 10.24925/turjaf.v11i3.603-616.5726.

21. Chaudhary A, Marinangeli CPF, Tremorin D, Mathys A. Nutritional Combined Greenhouse Gas Life Cycle Analysis for Incorporating Canadian Yellow Pea into Cereal-Based Food Products. Nutrients. 2018;10(4):490. doi: 10.3390/nu10040490.

22. Clodoveo ML, Tarsitano E, Crupi P, Pasculli L, Piscitelli P, Miani A, et al. Towards a new food labelling system for sustainable food production and healthy responsible consumption: The Med Index Checklist. J Funct Foods. 2022;98. doi: ARTN 105277

10.1016/j.jff.2022.105277. PubMed PMID: WOS:000874035100005.

23. Clodoveo ML, Tarsitano E, Sabbà C, Gesualdo L, Corbo F. Med-index: a food product labeling system to promote adherence to the mediterranean diet encouraging producers to make healthier and more sustainable food products. Italian Journal of Food Science. 2021;33(4):67-83. doi: 10.15586/ijfs.v33i4.2127. PubMed PMID: WOS:000745614200007.

24. Conrad Z, Stern A, Love DC, Salesses M, Cyril A, McDowell A, et al. Data Integration for Diet Sustainability Analyses. Sustainability. 2021;13(14):8082. doi: 10.3390/su13148082.

25. Cozzio C, Volgger M, Taplin R. Point-of-consumption interventions to promote virtuous food choices of tourists with self-benefit or other-benefit appeals: a randomised field experiment. Journal of Sustainable Tourism. 2022;30(6):1301-19. doi: 10.1080/09669582.2021.1932936.

26. Dahmani J, Nicklaus S, Grenier J-M, Marty L. Nutritional quality and greenhouse gas emissions of vegetarian and non-vegetarian primary school meals: A case study in Dijon, France. Frontiers in Nutrition. 2022;9. doi: 10.3389/fnut.2022.997144.

27. De Bauw M, Matthys C, Poppe V, Franssens S, Vranken L. A combined Nutri-Score and ‘Eco-Score’ approach for more nutritious and more environmentally friendly food choices? Evidence from a consumer experiment in Belgium. Food Qual Prefer. 2021;93:104276. doi: 10.1016/j.foodqual.2021.104276.

28. De Carvalho AM, Verly E, Marchioni DM, Jones AD. Measuring sustainable food systems in Brazil: A framework and multidimensional index to evaluate socioeconomic, nutritional, and environmental aspects. World Development. 2021;143. doi: ARTN 105470

10.1016/j.worlddev.2021.105470. PubMed PMID: WOS:000641982300003.

29. De Laurentiis V, Hunt DVL, Lee SE, Rogers CDF. EATS: a life cycle-based decision support tool for local authorities and school caterers. The International Journal of Life Cycle Assessment. 2019;24(7):1222-38. doi: 10.1007/s11367-018-1460-x.

30. Dernini S, Meybeck A, Burlingame B, Gitz V, Lacirignola C, Debs P, et al. Developing a methodological approach for assessing the sustainability of diets: The Mediterranean diet as a case study. A Mediterranean Journal of Economics, Agriculture and Environment. 2013;12(3):28-36.

31. Dernini S, Lairon D, Berry EM, Brunori G, Capone R, Donini LM, et al. The Med Diet 4.0 Framework: A Multidimensional Driver for Revitalising the Mediterranean Diet as a Sustainable Diet Model. Food Science and Nutrition Cases. 2023. doi: doi.org/10.1079/fsncases.2023.000.

32. Dogbe W, Revoredo-Giha C. Nutritional and Environmental Assessment of Increasing the Content of Fruit and Vegetables in the UK Diet. Sustainability. 2021;13(3):1076. doi: 10.3390/su13031076.

33. Donati M, Menozzi D, Zighetti C, Rosi A, Zinetti A, Scazzina F. Towards a sustainable diet combining economic, environmental and nutritional objectives. Appetite. 2016;106:48-57. doi: 10.1016/j.appet.2016.02.151. PubMed PMID: WOS:000383940400007.

34. Donini LM, Dernini S, Lairon D, Serra-Majem L, Amiot M-J, Del Balzo V, et al. A Consensus Proposal for Nutritional Indicators to Assess the Sustainability of a Healthy Diet: The Mediterranean Diet as a Case Study. Frontiers in Nutrition. 2016;3. doi: 10.3389/fnut.2016.00037.

35. Doran-Browne NA, Eckard RJ, Behrendt R, Kingwell RS. Nutrient density as a metric for comparing greenhouse gas emissions from food production. Clim Change. 2015;129(1-2):73-87. doi: 10.1007/s10584-014-1316-8.

36. Drewnowski A, Rehm CD, Martin A, Verger EO, Voinnesson M, Imbert P. Energy and nutrient density of foods in relation to their carbon footprint. Am J Clin Nutr. 2015;101(1):184-91. doi: 10.3945/ajcn.114.092486. PubMed PMID: WOS:000346895700021.

37. Drewnowski A. Measures and metrics of sustainable diets with a focus on milk, yogurt, and dairy products. Nutr Rev. 2018;76(1):21-8. doi: 10.1093/nutrit/nux063. PubMed PMID: WOS:000423835800002.

38. Eini-Zinab H, Sobhani SR, Rezazadeh A. Designing a healthy, low-cost and environmentally sustainable food basket: an optimisation study. Public Health Nutr. 2021;24(7):1952-61. doi: 10.1017/s1368980020003729.

39. El-Abbadi N, Peters C, Griffin T, Nelson M, Jacques P. Development of a dietary environmental index to assess nutritional quality versus environmental effect of foods and dietary patterns. The Lancet. 2017;389:S6. doi: 10.1016/s0140-6736(17)31118-2.

40. Esteve-Llorens X, Dias AC, Moreira MT, Feijoo G, González-García S. Evaluating the Portuguese diet in the pursuit of a lower carbon and healthier consumption pattern. Clim Change. 2020;162(4):2397-409. doi: 10.1007/s10584-020-02816-0.

41. Esteve-Llorens X, Darriba C, Moreira MT, Feijoo G, González-García S. Towards an environmentally sustainable and healthy Atlantic dietary pattern: Life cycle carbon footprint and nutritional quality. Sci Total Environ. 2019;646:704-15. doi: 10.1016/j.scitotenv.2018.07.264. PubMed PMID: WOS:000445164800068.

42. Esteve-Llorens X, Moreira MT, Feijoo G, González-García S. Linking environmental sustainability and nutritional quality of the Atlantic diet recommendations and real consumption habits in Galicia (NW Spain). Sci Total Environ. 2019;683:71-9. doi: 10.1016/j.scitotenv.2019.05.200. PubMed PMID: WOS:000471657600008.

43. Fernández-Ríos A, Laso J, Batlle-Bayer L, Amo-Setién F, Abajas-Bustillo R, Ortego-Maté C, et al. Design of a nutrient profiling model for life cycle assessment of “superfoods” to address nutritional deficiencies and enhance environmental protection in Spain. The International Journal of Life Cycle Assessment. 2025;30(4):694-706. doi: 10.1007/s11367-024-02426-3.

44. Fresán U, Craig WJ, Martínez-González MA, Bes-Rastrollo M. Nutritional Quality and Health Effects of Low Environmental Impact Diets: The “Seguimiento Universidad de Navarra” (SUN) Cohort. Nutrients. 2020;12(8):2385. doi: 10.3390/nu12082385.

45. García S, Bouzas C, Mateos D, Pastor R, Álvarez L, Rubín M, et al. Carbon dioxide (CO2) emissions and adherence to Mediterranean diet in an adult population: the Mediterranean diet index as a pollution level index. Environ Health. 2023;22(1). doi: 10.1186/s12940-022-00956-7.

46. Gazan R, Barré T, Perignon M, Maillot M, Darmon N, Vieux F. A methodology to compile food metrics related to diet sustainability into a single food database: Application to the French case. Food Chem. 2018;238:125-33. doi: 10.1016/j.foodchem.2016.11.083. PubMed PMID: WOS:000411351200019.

47. González-García S, Esteve-Llorens X, Moreira MT, Feijoo G. Carbon footprint and nutritional quality of different human dietary choices. Sci Total Environ. 2018;644:77-94. doi: 10.1016/j.scitotenv.2018.06.339. PubMed PMID: WOS:000445164000010.

48. González-Santana RA, Gómez-Urios C, Blesa J, Cortés C, Esteve MJ, Frígola A. Assessment of the Nutritional Composition and Environmental Impact of Menus Served in a University Cafeteria During an Academic Year. Dietetics. 2025;4(1):2. doi: 10.3390/dietetics4010002.

49. Graham F, Russell J, Holdsworth M, Menon M, Barker M. Exploring the Relationship between Environmental Impact and Nutrient Content of Sandwiches and Beverages Available in Cafés in a UK University. Sustainability. 2019;11(11):3190. doi: 10.3390/su11113190.

50. Green A, Nemecek T, Mathys A. A proposed framework to develop nutrient profiling algorithms for assessments of sustainable food: the metrics and their assumptions matter. The International Journal of Life Cycle Assessment. 2023;28(10):1326-47. doi: 10.1007/s11367-023-02210-9.

51. Gustafson DI, Decker EA, Drewnowski A, Hamm MW, Hwang J, Merrigan KA. Making Healthy, Sustainable Diets Accessible and Achievable: A New Framework for Assessing the Nutrition, Environmental, and Equity Impacts of Packaged Foods. Current Developments in Nutrition. 2022;6(10). doi: ARTN nzac136

10.1093/cdn/nzac136. PubMed PMID: WOS:000863740000001.

52. Gustafson D, Gutman A, Leet W, Drewnowski A, Fanzo J, Ingram J. Seven Food System Metrics of Sustainable Nutrition Security. Sustainability. 2016;8(3):196. doi: 10.3390/su8030196.

53. Hallström E, Bergman K, Mifflin K, Parker R, Tyedmers P, Troell M, et al. Combined climate and nutritional performance of seafoods. Journal of Cleaner Production. 2019;230:402-11. doi: 10.1016/j.jclepro.2019.04.229. PubMed PMID: WOS:000474676300036.

54. Hallström E, Röös E, Börjesson P. Sustainable meat consumption: A quantitative analysis of nutritional intake, greenhouse gas emissions and land use from a Swedish perspective. Food Policy. 2014;47:81-90. doi: doi.org/10.1016/j.foodpol.2014.04.002.

55. Harray A, Boushey C, Pollard C, Delp E, Ahmad Z, Dhaliwal S, et al. A Novel Dietary Assessment Method to Measure a Healthy and Sustainable Diet Using the Mobile Food Record: Protocol and Methodology. Nutrients. 2015;7(7):5375-95. doi: 10.3390/nu7075226.

56. Harray AJ, Boushey CJ, Pollard CM, Dhaliwal SS, Mukhtar SA, Delp EJ, et al. Healthy and Sustainable Diet Index: Development, Application and Evaluation Using Image-Based Food Records. Nutrients. 2022;14(18):3838. doi: 10.3390/nu14183838.

57. Herrmann M, Mehner E, Egger L, Portmann R, Hammer L, Nemecek T. A comparative nutritional life cycle assessment of processed and unprocessed soy-based meat and milk alternatives including protein quality adjustment. Frontiers in Sustainable Food Systems. 2024;8. doi: 10.3389/fsufs.2024.1413802.

58. Hobbs DA, Lovegrove JA, Givens DI. The role of dairy products in sustainable diets: modelling nutritional adequacy, financial and environmental impacts. Proc Nutr Soc. 2015;74(OCE5). doi: 10.1017/s0029665115003572.

59. Jolliet O. Integrating Dietary Impacts in Food Life Cycle Assessment. Frontiers in Nutrition. 2022;9. doi: 10.3389/fnut.2022.898180.

60. Kägi T, Zschokke M, Dinke F. Nutrient based functional unit for meals. 8th International Conference on LCA in the Agri-Food Sector; Rennes, France.2012.

61. Keding GB, Sarfo J, Pawelzik E. Healthy Diets from Sustainable Food Systems: Calculating the WISH Scores for Women in Rural East Africa. Nutrients. 2023;15(12):2699. doi: 10.3390/nu15122699.

62. Kesse-Guyot E, Chaltiel D, Wang J, Pointereau P, Langevin B, Allès B, et al. Sustainability analysis of French dietary guidelines using multiple criteria. Nature Sustainability. 2020;3(5):377-85. doi: 10.1038/s41893-020-0495-8.

63. Kesse-Guyot E, Allès B, Brunin J, Fouillet H, Dussiot A, Mariotti F, et al. Nutritionally adequate and environmentally respectful diets are possible for different diet groups: an optimized study from the NutriNet-Sante cohort. Am J Clin Nutr. 2022;116(6):1621-33. doi: 10.1093/ajcn/nqac253. PubMed PMID: WOS:000868955400001.

64. Kluczkovski A, Menezes CA, Da Silva JT, Bastos L, Lait R, Cook J, et al. An Environmental and Nutritional Evaluation of School Food Menus in Bahia, Brazil That Contribute to Local Public Policy to Promote Sustainability. Nutrients. 2022;14(7):1519. doi: 10.3390/nu14071519.

65. Kyttä V, Kårlund A, Pellinen T, Tuomisto HL, Kolehmainen M, Pajari AM, et al. Extending the product-group-specific approach in nutritional life cycle assessment. International Journal of Life Cycle Assessment. 2023. doi: 10.1007/s11367-023-02235-0. PubMed PMID: WOS:001075496400001.

66. Kyttä V, Kårlund A, Pellinen T, Pietiläinen O, Tuomisto HL, Kolehmainen M, et al. Product-group-specific nutrient index as a nutritional functional unit for the Life Cycle Assessment of protein-rich foods. The International Journal of Life Cycle Assessment. 2023;28(12):1672-88. doi: 10.1007/s11367-023-02217-2.

67. Kyttä V, Ghani HU, Pellinen T, Kårlund A, Kolehmainen M, Pajari AM, et al. Integrating nutrition into environmental impact assessments reveals limited sustainable food options within planetary boundaries. Sustainable Production and Consumption. 2025;56:142-55. doi: 10.1016/j.spc.2025.03.018. PubMed PMID: WOS:001462791300001.

68. Lima LB, Akutsu RDCD, Botelho RA, Nakano EY. AVACARD-Menu evaluation index: Construction and validation. International Journal of Gastronomy and Food Science. 2023;31. doi: ARTN 100671

10.1016/j.ijgfs.2023.100671. PubMed PMID: WOS:000927411900001.

69. Liyanapathirana NN, Grech A, Li MY, Malik A, Lenzen M, Raubenheimer D. Nutrient-sensitive approach for sustainability assessment of different dietary patterns in Australia. Am J Clin Nutr. 2022;115(4):1048-58. doi: 10.1093/ajcn/nqab429. PubMed PMID: WOS:000756145900001.

70. Long Y, Huang L, Fujie R, He P, Chen Z, Xu X, et al. Carbon footprint and embodied nutrition evaluation of 388 recipes. Scientific Data. 2023;10(1). doi: 10.1038/s41597-023-02702-1.

71. Lukas M, Palzkill A, Rohn H, C L. The nutritional footprint: an innovative management approach for the food sector WIT Transactions on Ecology and The Environment. 2013;170. doi: doi:10.2495/FENV130011.

72. Martins ML, Tepper S, Marques B, Abreu S. The SHED Index: A Validation Study to Assess Sustainable HEalthy Diets in Portugal. Nutrients. 2023;15(24):5071. doi: 10.3390/nu15245071.

73. Marty L, Teil F, Lange C, Bellassen V, Visalli M. Development and validation of a web application to collect food supply data associated with their nutritional composition and environmental impacts. MethodsX. 2024;13:102891. Epub 20240813. doi: 10.1016/j.mex.2024.102891. PubMed PMID: 39263360; PubMed Central PMCID: PMCPMC11388661.

74. McAuliffe GA, Takahashi T, Beal T, Huppertz T, Leroy F, Buttriss J, et al. Protein quality as a complementary functional unit in life cycle assessment (LCA). The International Journal of Life Cycle Assessment. 2023;28(2):146-55. doi: 10.1007/s11367-022-02123-z.

75. Mungkung R, Dangsiri S, Satmalee P, Surojanametakul V, Saejew K, Gheewala SH. The nutrition-environment nexus assessment of Thai Riceberry product for supporting environmental product declaration. Environ Dev Sustainability. 2023;26(2):4487-503. doi: 10.1007/s10668-022-02892-5.

76. Nakamura K, Itsubo N. Environmental and Health-Related Lifecycle Impact Assessment of Reduced-Salt Meals in Japan. Sustainability. 2022;14(14):8265. doi: 10.3390/su14148265.

77. Nguyen SD, Biesbroek S, Le TD, Feskens EJM, Brouwer ID, Talsma EF. Environmental impact and nutrient adequacy of derived dietary patterns in Vietnam. Frontiers in Nutrition. 2023;10. doi: 10.3389/fnut.2023.986241.

78. O'Malley K, Willits-Smith A, Aranda R, Heller M, Rose D. Vegan vs Paleo: Carbon Footprints and Diet Quality of 5 Popular Eating Patterns as Reported by US Consumers (P03-007-19). Current Developments in Nutrition. 2019;3(nzz047):03-007-19. doi: 10.1093/cdn/nzz047.P03-007-19.

79. Perignon M, Masset G, Ferrari G, Barré T, Vieux F, Maillot M, et al. How low can dietary greenhouse gas emissions be reduced without impairing nutritional adequacy, affordability and acceptability of the diet? A modelling study to guide sustainable food choices. Public Health Nutr. 2016;19(14):2662-74. doi: 10.1017/s1368980016000653.

80. Petruzzelli M, García-Herrero L, De Menna F, Vittuari M. Towards sustainable school meals: integrating environmental and cost implications for nutritious diets through optimisation modelling. Sustainability Science. 2023. doi: 10.1007/s11625-023-01346-9.

81. Philippi Rosane B, Matthiessen LE, Góralska-Walczak R, Kopczyńska K, Średnicka-Tober D, Kazimierczak R, et al. Development of a methodology to compare and evaluate health and sustainability aspects of dietary intake across countries. Frontiers in Sustainable Food Systems. 2023;7. doi: 10.3389/fsufs.2023.1147874.

82. Pink AE, Stylianou KS, Lee LL, Jolliet O, Cheon BK. The effects of presenting health and environmental impacts of food on consumption intentions. Food Qual Prefer. 2022;98. doi: ARTN 104501

10.1016/j.foodqual.2021.104501. PubMed PMID: WOS:000743157100017.

83. Potter C, Pechey R, Cook B, Bateman P, Stewart C, Frie K, et al. Effects of environmental impact and nutrition labelling on food purchasing: An experimental online supermarket study. Appetite. 2023;180:106312. Epub 20220921. doi: 10.1016/j.appet.2022.106312. PubMed PMID: 36150553.

84. Prosperi P. Sustainability and food and nutrition security: an indicator-based vulnerability and resilience approach for the Mediterranean Region. Italian Review of Agricultural Economics. 2016;71. doi: 10.13128/rea-18677.

85. Rocha A, Viegas C. KIMEHS—Proposal of an Index for Qualitative Evaluation of Children’s Menus—A Pilot Study. Foods. 2020;9(11):1618. doi: 10.3390/foods9111618.

86. Röös E, Jacobsen M, Karlsson L, Wanecek W, Spångberg J, Mazac R, et al. Introducing a comprehensive and configurable tool for calculating environmental and social footprints for use in dietary assessments. Journal of Cleaner Production. 2025;519. doi: ARTN 146002

10.1016/j.jclepro.2025.146002. PubMed PMID: WOS:001537879900002.

87. Rosi A, Biasini B, Monica E, Rapetti V, Deon V, Scazzina F. Nutritional Composition and Environmental Impact of Meals Selected in Workplace Canteens before and after an Intervention Promoting the Adherence to the Mediterranean Diet. Nutrients. 2022;14(21):4456. doi: 10.3390/nu14214456.

88. Ruini LF, Ciati R, Pratesi CA, Marino M, Principato L, Vannuzzi E. Working toward Healthy and Sustainable Diets: The “Double Pyramid Model” Developed by the Barilla Center for Food and Nutrition to Raise Awareness about the Environmental and Nutritional Impact of Foods. Frontiers in Nutrition. 2015;2. doi: 10.3389/fnut.2015.00009.

89. Saarinen M, Fogelholm M, Tahvonen R, Kurppa S. Taking nutrition into account within the life cycle assessment of food products. Journal of Cleaner Production. 2017;149:828-44. doi: 10.1016/j.jclepro.2017.02.062. PubMed PMID: WOS:000403330200073.

90. Saleki N, Kulaksiz SB, Arslan F, Coskun MG. The evaluation of menus' adherence to sustainable nutrition and comparison with sustainable menu example in a Turkish university refectory. Nutr Food Sci. 2023;53(8):1293-303. doi: 10.1108/Nfs-10-2022-0364. PubMed PMID: WOS:000998612800001.

91. Schumacher TL, Alderton CA, Brown LJ, Heaney S, Alston L, Kent K, et al. Development of a Scoring Tool for Australian Rural Food Retail Environments. Nutrients. 2023;15(21):4660. doi: 10.3390/nu15214660.

92. Seconda L, Baudry J, Allès B, Boizot-Szantai C, Soler L-G, Galan P, et al. Comparing nutritional, economic, and environmental performances of diets according to their levels of greenhouse gas emissions. Clim Change. 2018;148(1-2):155-72. doi: 10.1007/s10584-018-2195-1.

93. Simon X, Copena D, Pérez-Neira D. Assessment of the diet-environment-health-cost quadrilemma in public school canteens. an LCA case study in Galicia (Spain). Environ Dev Sustainability. 2023;25(11):12543-67. doi: 10.1007/s10668-022-02578-y.

94. Singh-Povel CM, Van Gool MP, Gual Rojas AP, Bragt MC, Kleinnijenhuis AJ, Hettinga KA. Nutritional content, protein quantity, protein quality and carbon footprint of plant-based drinks and semi-skimmed milk in the Netherlands and Europe. Public Health Nutr. 2022;25(5):1416-26. doi: 10.1017/s1368980022000453.

95. Sonesson U, Davis J, Flysjö A, Gustaysson J, Witthöft C. Protein quality as functional unit - A methodological framework for. inclusion in life cycle assessment of food. Journal of Cleaner Production. 2017;140:470-8. doi: 10.1016/j.jclepro.2016.06.115. PubMed PMID: WOS:000388775200008.

96. Strasburg VJ, Prattes G, Acevedo B, Suárez C. Calidad nutricional e impacto en medio ambiente por los insumos de un comedor universitario en Uruguay. Arch Latinoam Nutr. 2023;73(2):90-101. doi: 10.37527/2023.73.2.001.

97. Strid A, Hallström E, Sonesson U, Sjons J, Winkvist A, Bianchi M. Sustainability Indicators for Foods Benefiting Climate and Health. Sustainability. 2021;13(7):3621. doi: 10.3390/su13073621.

98. Sturtewagen L, De Soete W, Dewulf J, Lachat C, Lauryssen S, Heirman B, et al. Resource use profile and nutritional value assessment of a typical Belgian meal, catered or home cooked, with pork or Quorn™ as protein source. Journal of Cleaner Production. 2016;112:196-204. doi: 10.1016/j.jclepro.2015.09.006. PubMed PMID: WOS:000368206700021.

99. Sugimoto M, Temme EHM, Biesbroek S, Kanellopoulos A, Okubo H, Fujiwara A, et al. Exploring culturally acceptable, nutritious, affordable and low climatic impact diet for Japanese diets: proof of concept of applying a new modelling approach using data envelopment analysis. Br J Nutr. 2022;128(12):2438-52. doi: 10.1017/s0007114522000095.

100. Takacs B, Kalea AZ, Borrion A. An integrated assessment of the environmental and nutritional impacts of different types of meals using life cycle assessment. Proc Nutr Soc. 2021;80(OCE5). doi: 10.1017/s0029665121002901.

101. Tepper S, Geva D, Shahar DR, Shepon A, Mendelsohn O, Golan M, et al. The SHED Index: a tool for assessing a Sustainable HEalthy Diet. Eur J Nutr. 2021;60(7):3897-909. doi: 10.1007/s00394-021-02554-8.

102. Totland TH, Øvrebø B, Brantsæter AL, Holvik K, Bere ET, Torheim LE, et al. Development and evaluation of an index assessing adherence to the Norwegian food-based dietary guidelines: the Norwegian Dietary Guideline Index (NDGI). BMC Nutrition. 2024;10(1). doi: 10.1186/s40795-024-00900-7.

103. Travassos GF, Da Cunha DA, Coelho AB. Environmental and nutritional perspective of a more sustainable meat consumption in Brazil. Environ Dev Sustainability. 2023. doi: 10.1007/s10668-023-03941-3.

104. Van De Kamp M, Temme E. Plant-Based Lunch at Work: Effects on Nutrient Intake, Environmental Impact and Tastiness—A Case Study. Sustainability. 2018;10(1):227. doi: 10.3390/su10010227.

105. van Dooren C, Douma A, Aiking H, Vellinga P. Proposing a Novel Index Reflecting Both Climate Impact and Nutritional Impact of Food Products. Ecol Econ. 2017;131:389-98. doi: 10.1016/j.ecolecon.2016.08.029. PubMed PMID: WOS:000388248600035.

106. Van Dooren C, Aiking H. Defining a nutritionally healthy, environmentally friendly, and culturally acceptable Low Lands Diet. The International Journal of Life Cycle Assessment. 2016;21(5):688-700. doi: 10.1007/s11367-015-1007-3.

107. Van Kernebeek HRJ, Oosting SJ, Feskens EJM, Gerber PJ, De Boer IJM. The effect of nutritional quality on comparing environmental impacts of human diets. Journal of Cleaner Production. 2014;73:88-99. doi: 10.1016/j.jclepro.2013.11.028. PubMed PMID: WOS:000337852000011.

108. Van Mierlo K, Rohmer S, Gerdessen JC. A model for composing meat replacers: Reducing the environmental impact of our food consumption pattern while retaining its nutritional value. Journal of Cleaner Production. 2017;165:930-50. doi: 10.1016/j.jclepro.2017.07.098.

109. Walker C, Pfister S, Hellweg S. Methodology and optimization tool for a personalized low environmental impact and healthful diet specific to country and season. Journal of Industrial Ecology. 2021;25(5):1147-60. doi: 10.1111/jiec.13131.

110. Walker C, Gibney ER, Hellweg S. Comparison of Environmental Impact and Nutritional Quality among a European Sample Population – findings from the Food4Me study. Sci Rep. 2018;8(1). doi: 10.1038/s41598-018-20391-4.

111. Wilson N, Nghiem N, Ni Mhurchu C, Eyles H, Baker MG, Blakely T. Foods and Dietary Patterns That Are Healthy, Low-Cost, and Environmentally Sustainable: A Case Study of Optimization Modeling for New Zealand. PLoS One. 2013;8(3):e59648. doi: 10.1371/journal.pone.0059648.

112. Wrieden WL, Leinonen I, Barton KL, Halligan J, Goffe L. Is the UK diet sustainable? Assessing the environmental impact, cost and nutritional quality of household food purchases. Proc Nutr Soc. 2017;76(OCE3). doi: 10.1017/s0029665117001811.

113. Wright EC, Van Oort B, Bjøntegaard MM, Carlsen MH, Andersen LF. Environmental and nutritional assessment of young children’s diets in Norway: comparing the current diet with national dietary guidelines and the EAT-Lancet reference diet. Eur J Nutr. 2023;62(8):3383-96. doi: 10.1007/s00394-023-03243-4.

114. Xu ZY, Xu WJ, Peng ZJ, Yang QY, Zhang ZH. Effects of different functional units on carbon footprint values of different carbohydrate-rich foods in China. Journal of Cleaner Production. 2018;198:907-16. doi: 10.1016/j.jclepro.2018.07.091. PubMed PMID: WOS:000442973100079.

115. Yue WC, Tan ZK, Zhang JM, Zeng JN, Xu M, Rong QQ, et al. Optimization of residents' dietary structure with consideration of greenhouse gas mitigation and nutritional requirements. Sustainable Production and Consumption. 2022;32:424-35. doi: 10.1016/j.spc.2022.04.030. PubMed PMID: WOS:000806368300007.

116. Żakowska-Biemans S, Pieniak Z, Kostyra E, Gutkowska K. Searching for a Measure Integrating Sustainable and Healthy Eating Behaviors. Nutrients. 2019;11(1):95. doi: 10.3390/nu11010095.

117. Zhan JD, Bui L, Hodge RA, Zimmer M, Pham T, Rose D, et al. Planetary Health Diet Index Trends and Associations with Dietary Greenhouse Gas Emissions, Disease Biomarkers, Obesity, and Mortality in the United States (2005-2018). Am J Clin Nutr. 2025;121(3):580-8. doi: 10.1016/j.ajcnut.2025.01.007. PubMed PMID: WOS:001439172600001.

118. Zhao R, Yang X, Lin J, Xu Z, Chen Q. A Comparative Study on Carbon Footprints between Wheat Flour and Potato in China Considering the Nutrition Function of Foods. IOP Conf Series: Earth and Environmental Science. 2021;726. doi: doi:10.1088/1755-1315/726/1/012004.

119. Zulkefli N, Moy F-M. Development and Validation of a Sustainable Diet Index among Malaysian Adults: Protocol. Sains Malaysiana. 2021;50(6):1697-705. doi: 10.17576/jsm-2021-5006-16.

120. Aidoo R, Romana CK, Kwofie EM, Baum JI. An integrated environmental nutrition model for dietary sustainability assessment. Journal of Cleaner Production. 2023;399. doi: ARTN 136473

10.1016/j.jclepro.2023.136473. PubMed PMID: WOS:000953464700001.

121. Bach LY, Jana BE, Egwatu CFA, Orndorff CJ, Alanakrih R, Okoro J, et al. A sustainability analysis of environmental impact, nutritional quality, and price among six popular diets. Frontiers in Sustainable Food Systems. 2023;7. doi: ARTN 1021906

10.3389/fsufs.2023.1021906. PubMed PMID: WOS:000963236000001.

122. Battle-Bayer L, Bala A, Lemaire E, Albertí J, García-Herrero I, Aldaco R, et al. An energy- and nutrient-corrected functional unit to compare LCAs of diets. Sci Total Environ. 2019;671:175-9. doi: 10.1016/j.scitotenv.2019.03.332. PubMed PMID: WOS:000466090500020.

123. Batlle-Bayer L, Bala A, Albertí J, Xifré R, Aldaco R, Fullana-i-Palmer P. Food affordability and nutritional values within the functional unit of a food LCA. An application on regional diets in Spain. Resources Conservation and Recycling. 2020;160. doi: ARTN 104856

10.1016/j.resconrec.2020.104856. PubMed PMID: WOS:000540613000014.

124. Cooreman-Algoed M, Huysveld S, Lachat C, Dewulf J. How to integrate nutritional recommendations and environmental policy targets at the meal level: A university canteen example. Sustainable Production and Consumption. 2020;21:120-31. doi: 10.1016/j.spc.2019.10.004. PubMed PMID: WOS:000529312000009.

125. Costa Da Silva TT, Falco BB, Gomes De Castro I, Zanon RB, Guerra VVJ, Yaginuma YK, et al. Carbon, Water, Ecological Footprints, Energy and Nutritional Densities of Omnivore and Vegan Culinary Preparations. Food and Nutrition Sciences. 2023;14(7). doi: 10.4236/fns.2023.147041

126. Dourmad J, Y. , van der Werf H, M,G., Mairesse G, Schmitt B, Chesneau G, Kerhoas N, et al. Multidimensional evaluation and development of a tool for the improvement of sustainability of menus. Cahiers de Nutrition et de Diététique. 2019;54:223-9. doi: 10.1016/j.cnd.2019.03.002.

127. Fresan U, Martinez-Gonzalez MA, Segovia-Siapco G, Sabate J, Bes-Rastrollo M. A three-dimensional dietary index (nutritional quality, environment and price) and reduced mortality: The "Seguimiento Universidad de Navarra" cohort. Prev Med. 2020;137:106124. Epub 20200508. doi: 10.1016/j.ypmed.2020.106124. PubMed PMID: 32437702.

128. Goss MA, Sherwood J. An absolute environmental sustainability assessment of food. Food Frontiers. 2024;5(3):855-66. doi: 10.1002/fft2.371.

129. Guido YDS, Fonseca G, Soares AD, da Silva ECN, Ostanik PAG, Perobelli JE. Food-triad: An index for sustainable consumption. Sci Total Environ. 2020;740. doi: ARTN 140027

10.1016/j.scitotenv.2020.140027. PubMed PMID: WOS:000562379300005.

130. Haupt M. RSJ, Clemente Polo G., García-Segovia P., Sanjuán Pellicer N. Approach to the integration of the carbon footprint and nutritional aspects for sustainable food consumption. Revista Española de Nutrición Comunitaria. 2016;22(1):2-9. doi: 10.14642/RENC.2016.22.1.5124.

131. Hooker K, Sanjeevi N, Monsivais P. Identifying Optimally Sustainable Foods: A Four-Dimensional Analysis of Sustainable Foods in the American Diet. Sustainability. 2024;16(2). doi: ARTN 551

10.3390/su16020551. PubMed PMID: WOS:001152966400001.

132. Kong FZ, Cui WL, Bao SW. Dynamic changes and sustainability assessment of food consumption footprint in megacities: A comparative analysis from four Chinese municipalities. Sustainable Cities and Society. 2025;127. doi: ARTN 106433

10.1016/j.scs.2025.106433. PubMed PMID: WOS:001497901300003.

133. Li YY, Filimonau V, Wang LE, Cheng SK. A set of preliminary indicators for holistic sustainability assessment of household food consumption in rural and urban China. Resources Conservation and Recycling. 2023;188. doi: ARTN 106727

10.1016/j.resconrec.2022.106727. PubMed PMID: WOS:000886559600001.

134. Lukas M, Rohn H, Lettenmeier M, Liedtke C, Wiesen K. The nutritional footprint - integrated methodology using environmental and health indicators to indicate potential for absolute reduction of natural resource use in the field of food and nutrition. Journal of Cleaner Production. 2016;132:161-70. doi: 10.1016/j.jclepro.2015.02.070. PubMed PMID: WOS:000380624400013.

135. Meier T, Schade S, Forner F, Eberle U. Bridging Nutritional and Environmental Sustainability Within Planetary Boundaries in Food Life Cycle Assessments: SWOT Review and Development of the Planet Health Conformity Index. Sustainability. 2024;16(23):10658. doi: 10.3390/su162310658.

136. Röös E, Karlsson H, Witthöft C, Sundberg C. Evaluating the sustainability of diets-combining environmental and nutritional aspects. Environ Sci Policy. 2015;47:157-66. doi: 10.1016/j.envsci.2014.12.001. PubMed PMID: WOS:000349581400015.

137. Schaubroeck T, Ceuppens S, Luong AD, Benetto E, De Meester S, Lachat C, et al. A pragmatic framework to score and inform about the environmental sustainability and nutritional profile of canteen meals, a case study on a university canteen. Journal of Cleaner Production. 2018;187:672-86. doi: 10.1016/j.jclepro.2018.03.265. PubMed PMID: WOS:000432102500057.

138. Seconda L, Baudry J, Pointereau P, Lacour C, Langevin B, Hercberg S, et al. Development and validation of an individual sustainable diet index in the NutriNet-Santé study cohort. Br J Nutr. 2019;121(10):1166-77. doi: 10.1017/s0007114519000369.

139. Sonesson U, Davis J, Hallström E, Woodhouse A. Dietary-dependent nutrient quality indexes as a complementary functional unit in LCA: A feasible option? Journal of Cleaner Production. 2019;211:620-7. doi: https://doi.org/10.1016/j.jclepro.2018.11.171.

140. Strid A, Hallström E, Sonesson U, Sjons J, Winkvist A, Bianchi M. Sustainability Indicators for Foods Benefiting Climate and Health. Sustainability. 2021;13(7). doi: ARTN 362110.3390/su13073621. PubMed PMID: WOS:000638930300001.

141. Takacs B, Kalea AZ, Borrion A. Menu Dilemmas: An Integrated Assessment of the Nutritional Quality, Environmental Impact, and Cost of Vegan, Vegetarian, and Meat-Based Versions of Meals. Nutrients. 2025;17(9):1569. doi: 10.3390/nu17091569.

142. Trijsburg L, Talsma EF, Crispim SP, Garrett J, Kennedy G, de Vries JHM, et al. Method for the Development of WISH, a Globally Applicable Index for Healthy Diets from Sustainable Food Systems. Nutrients. 2021;13(1). doi: ARTN 93

10.3390/nu13010093. PubMed PMID: WOS:000611091600001.

143. van Dooren C, Marinussen M, Blonk H, Aiking H, Vellinga P. Exploring dietary guidelines based on ecological and nutritional values: A comparison of six dietary patterns. Food Policy. 2014;44:36-46. doi: 10.1016/j.foodpol.2013.11.002. PubMed PMID: WOS:000332751200004.

144. Werner LB, Flysjö A, Tholstrup T. Greenhouse gas emissions of realistic dietary choices in Denmark: the carbon footprint and nutritional value of dairy products. Food Nutr Res. 2014;58. doi: ARTN 20687

10.3402/fnr.v58.20687. PubMed PMID: WOS:000338349700001.

145. Sherwood J. Calculating the sustainability of products based on their efficiency and function. One Earth. 2022;5(11):1260-70. doi: 10.1016/j.oneear.2022.10.011.

146. Shi CF, Wu C, Zhang JY, Zhang CJ, Xiao QQ. Impact of urban and rural food consumption on water demand in China-From the perspective of water footprint. Sustainable Production and Consumption. 2022;34:148-62. doi: 10.1016/j.spc.2022.09.006. PubMed PMID: WOS:000869003800001.

147. Huang YY, Tian X. Food accessibility, diversity of agricultural production and dietary pattern in rural China. Food Policy. 2019;84:92-102. doi: 10.1016/j.foodpol.2019.03.002. PubMed PMID: WOS:000466454000009.

148. Quan S, Li M, Zhu W. Dietary diversity: A systematic measurement. World Agriculture. 2024;1:52–65. doi: https://doi.org/10.13856/j.cn11-1097/s.2024.01.005.

149. Forner F, Volkhardt I, Meier T, Christen O, Stangl GI. The nutriRECIPE-Index – development and validation of a nutrient-weighted index for the evaluation of recipes. BMC Nutrition. 2021;7(1). doi: 10.1186/s40795-021-00483-7.

150. Drewnowski A. Defining Nutrient Density: Development and Validation of the Nutrient Rich Foods Index. J Am Coll Nutr. 2009;28(4):421S-6S. doi: 10.1080/07315724.2009.10718106.

151. Fulgoni VL, Keast DR, Drewnowski A. Development and Validation of the Nutrient-Rich Foods Index: A Tool to Measure Nutritional Quality of Foods. J Nutr. 2009;139(8):1549-54. doi: 10.3945/jn.108.101360. PubMed PMID: WOS:000268362000018.

152. Üçtuğ FG. The Environmental Life Cycle Assessment of Dairy Products. Food Engineering Reviews. 2019;11(2):104-21. doi: 10.1007/s12393-019-9187-4.

153. Masset G, Soler L-G, Vieux F, Darmon N. Identifying Sustainable Foods: The Relationship between Environmental Impact, Nutritional Quality, and Prices of Foods Representative of the French Diet. J Acad Nutr Diet. 2014;114(6):862-9. doi: 10.1016/j.jand.2014.02.002.

154. Batlle-Bayer L, Bala A, García-Herrero I, Lemaire E, Song G, Aldaco R, et al. The Spanish Dietary Guidelines: A potential tool to reduce greenhouse gas emissions of current dietary patterns. Journal of Cleaner Production. 2019;213:588-98. doi: 10.1016/j.jclepro.2018.12.215.

155. Commission. E. A Roadmap for moving to a competitive low carbon economy in 2050. Brussels: 2011.

156. Garzillo JMF, Machado PP, Louzada MLCL, Levy RB, Monteiro CA. Pegadas dos alimentos e das preparações culinárias consumidos no Brasil. 2019.

157. Pernollet F, Coelho CRV, van der Werf HMG. Methods to simplify diet and food life cycle inventories: Accuracy versus data-collection resources. Journal of Cleaner Production. 2017;140:410-20. doi: 10.1016/j.jclepro.2016.06.111. PubMed PMID: WOS:000388775200003.

158. Fresán U, Martínez-Gonzalez M-A, Sabaté J, Bes-Rastrollo M. The Mediterranean diet, an environmentally friendly option: evidence from the Seguimiento Universidad de Navarra (SUN) cohort. Public Health Nutr. 2018;21(8):1573-82. doi: 10.1017/s1368980017003986.

159. Ryberg MW, Andersen MM, Owsianiak M, Hauschild MZ. Downscaling the planetary boundaries in absolute environmental sustainability assessments - A review. Journal of Cleaner Production. 2020;276. doi: ARTN 123287

10.1016/j.jclepro.2020.123287. PubMed PMID: WOS:000579500800091.

160. Djekic I, Sanjuán N, Clemente G, Jambrak AR, Djukic-Vukovic A, Brodnjak UV, et al. Review on environmental models in the food chain - Current status and future perspectives. Journal of Cleaner Production. 2018;176:1012-25. doi: 10.1016/j.jclepro.2017.11.241. PubMed PMID: WOS:000423648000090.

161. Jungbluth N, Tietje O, Scholz RW. Food purchases: Impacts from the consumers’ point of view investigated with a modular LCA. The International Journal of Life Cycle Assessment. 2000;5(3):134-42. doi: 10.1007/bf02978609.

162. Roches A, Nemecek T, Gaillard G, Plassmann K, Sim S, King H, et al. MEXALCA: a modular method for the extrapolation of crop LCA. The International Journal of Life Cycle Assessment. 2010;15(8):842-54. doi: 10.1007/s11367-010-0209-y.

163. Poore J, Nemecek T. Reducing food’s environmental impacts through producers and consumers. Science. 2018;360(6392):987-92. doi: 10.1126/science.aaq0216.

164. Chen DD, Gao WS, Chen YQ, Zhang Q. Ecological footprint analysis of food consumption of rural residents in China in the latest 30 years. International Conference on Agricultural Risk and Food Security 2010. 2010;1:106-15. doi: 10.1016/j.aaspro.2010.09.013. PubMed PMID: WOS:000287099300012.

165. Naja F, Jomaa L, Itani L, Zidek J, El Labban S, Sibai AM, et al. Environmental footprints of food consumption and dietary patterns among Lebanese adults: a cross-sectional study. Nutr J. 2018;17. doi: ARTN 85

10.1186/s12937-018-0393-3. PubMed PMID: WOS:000444532400001.

166. Costanza R, dArge R, deGroot R, Farber S, Grasso M, Hannon B, et al. The value of the world's ecosystem services and natural capital. Nature. 1997;387(6630):253-60. doi: DOI 10.1038/387253a0. PubMed PMID: WOS:A1997WZ16700043.

167. Xie GD, Zhang CX, Zhen L, Zhang LM. Dynamic changes in the value of China's ecosystem services. Ecosystem Services. 2017;26:146-54. doi: 10.1016/j.ecoser.2017.06.010. PubMed PMID: WOS:000414208000015.

168. Kim B, Neff R. Measurement and communication of greenhouse gas emissions from U.S. food consumption via carbon calculators. Ecol Econ. 2009;69(1):186-96. doi: 10.1016/j.ecolecon.2009.08.017.

169. Lettenmeier M, Liedtke C, Rohn H. Eight Tons of Material Footprint—Suggestion for a Resource Cap for Household Consumption in Finland. Resources. 2014;3(3):488-515. doi: 10.3390/resources3030488.

170. Macdiarmid JI, Kyle J, Horgan GW, Loe J, Fyfe C, Johnstone A, et al. Sustainable diets for the future: can we contribute to reducing greenhouse gas emissions by eating a healthy diet? Am J Clin Nutr. 2012;96(3):632-9. doi: 10.3945/ajcn.112.038729. PubMed PMID: WOS:000307863800023.

171. Mekonnen MM, Hoekstra AY. A Global Assessment of the Water Footprint of Farm Animal Products. Ecosystems. 2012;15(3):401-15. doi: 10.1007/s10021-011-9517-8.

172. Rockström J, Steffen W, Noone K, Persson Å, Chapin FS, Lambin EF, et al. A safe operating space for humanity. Nature. 2009;461(7263):472-5. doi: 10.1038/461472a.

173. Agency U-FE. Klimaneutral leben: Verbraucher starten durch beim Klimaschutz. 2010.

174. Noleppa S. Climate Change on Our Plates. Berlin: WWF; 2012 [cited 2024 07 Oct]. Available from: www.wwf.de/fileadmin/fm-wwf/Publikationen-PDF/Klimawandel_auf_dem_ Teller.pdf.

175. Von Witzke H, Noleppa S, Zhirkova I. Meat Eats Land. . Berlin, Germany: WWF, 2011.

176. Willett W, Rockström J, Loken B, Springmann M, Lang T, Vermeulen S, et al. Food in the Anthropocene: the EAT–Lancet Commission on healthy diets from sustainable food systems. The Lancet. 2019;393(10170):447-92. doi: 10.1016/s0140-6736(18)31788-4.

177. Röös E. Mat-klimat-listan. Uppsala, Sweden: Sveriges lantbruksuniversitet, 2012 040.

178. De Baan L, Alkemade R, Koellner T. Land use impacts on biodiversity in LCA: a global approach. The International Journal of Life Cycle Assessment. 2013;18(6):1216-30. doi: 10.1007/s11367-012-0412-0.

179. Dewulf J, Bösch ME, Meester BD, Vorst GVd, Langenhove HV, Hellweg S, et al. Cumulative Exergy Extraction from the Natural Environment (CEENE): a comprehensive Life Cycle Impact Assessment method for resource accounting. Environ Sci Technol. 2007;41(24):8477-83. doi: 10.1021/es0711415.

180. Alvarenga RAF, Dewulf J, Van Langenhove H, Huijbregts MAJ. Exergy-based accounting for land as a natural resource in life cycle assessment. The International Journal of Life Cycle Assessment. 2013;18(5):939-47. doi: 10.1007/s11367-013-0555-7.

181. Pfister S, Koehler A, Hellweg S. Assessing the Environmental Impacts of Freshwater Consumption in LCA. Environ Sci Technol. 2009;43(11):4098-104. doi: 10.1021/es802423e.

182. Floren B, Amani P, Davis J. Climate Database Facilitating Climate Smart Meal Planning for the Public Sector in Sweden International Journal on Food System Dynamics. 2017;8(1):72-80. doi: 10.18461/ijfsd.v8i1.816

183. ISO. ISO 14040:2006. Environmental management — Life cycle assessment — Principles and framework. Geneva, Switzerland: International Organization for Standardization, 2006 ISO 14040:2006.

184. ISO. ISO 14044:2006. Environmental management — Life cycle assessment — Requirements and guidelines. Geneva, Switzerland: International Organization for Standardization, 2006.

185. Clark MA, Springmann M, Hill J, Tilman D. Multiple health and environmental impacts of foods. Proc Natl Acad Sci U S A. 2019;116(46):23357-62. doi: 10.1073/pnas.1906908116. PubMed PMID: WOS:000496506600069.

186. Blonk H, Ponsioen T, Kool A, Marinussen M. The Agri-Foorprint Method; Methodological LCA Framework, Assumptions and Applied Data, Version 1.0. Gouda, Netherlands: 2011.

187. BSI. PAS 2050:2008 Specification for The Assessment of The Life Cycle Greenhouse Gas Emissions of Goods and Services. . London, UK: British Standards, Carbon Trust, Defra, 2008.

188. Nations. FaAOotU. Sustainable Food and Agriculture. 2023 [cited 2024 22 Nov]. Available from: https://www.fao.org/sustainability/en/.

189. Masset G, Vieux F, Darmon N. Which functional unit to identify sustainable foods? Public Health Nutr. 2015;18(13):2488-97. doi: 10.1017/s1368980015000579.

190. Masset G, Vieux F, Verger EO, Soler LG, Touazi D, Darmon N. Reducing energy intake and energy density for a sustainable diet: a study based on self-selected diets in French adults. Am J Clin Nutr. 2014;99(6):1460-9. doi: 10.3945/ajcn.113.077958. PubMed PMID: WOS:000336470100019.

191. Smedman A, Lindmark-Månsson H, Drewnowski A, Edman A-KM. Nutrient density of beverages in relation to climate impact. Food &amp; Nutrition Research. 2010;54(1):5170. doi: 10.3402/fnr.v54i0.5170.

192. Fresán U, Martínez-González MA, Sabaté J, Bes-Rastrollo M. Global sustainability (health, environment and monetary costs) of three dietary patterns: results from a Spanish cohort (the SUN project). BMJ Open. 2019;9(2):e021541. doi: 10.1136/bmjopen-2018-021541.

193. Chapman PM, Anderson B, Carr S, Engle V, Green R, Hameedi J, et al. General guidelines for using the sediment quality triad. Mar Pollut Bull. 1997;34(6):368-72. doi: Doi 10.1016/S0025-326x(96)00138-5. PubMed PMID: WOS:A1997XR02600011.

194. Mortensen M, Langtangen HP. High performance Python for direct numerical simulations of turbulent flows. Computer Physics Communications. 2016;203:53-65. doi: 10.1016/j.cpc.2016.02.005.

195. Cooper WW, Seiford LM, Tone K. Introduction to Data Envelopment Analysis and Its Uses. USA: Springer; 2006.

196. Ray CS. Data Envelopment Analysis.

Theory and Techniques for Economics and Operations Research.: Cambridge University Press.; 2004.

197. Liu F, Zhang HQ. Novel methods to assess environmental, economic, and social sustainability of main agricultural regions in China. Agronomy for Sustainable Development. 2013;33(3):621-33. doi: 10.1007/s13593-012-0131-8. PubMed PMID: WOS:000319878300017.

198. Müller M, Wolfe SD, Gaffney C, Gogishvili D, Hug M, Leick A. An evaluation of the sustainability of the Olympic Games. Nature Sustainability. 2021;4(4):340-8. doi: 10.1038/s41893-021-00696-5.

199. Zhang X, Yao G, Vishwakarma S, Dalin C, Komarek AM, Kanter DR, et al. Quantitative assessment of agricultural sustainability reveals divergent priorities among nations. One Earth. 2021;4(9):1262-77. doi: 10.1016/j.oneear.2021.08.015.

200. Green A, Nemecek T, Mathys A. A proposed framework to develop nutrient profiling algorithms for assessments of sustainable food: the metrics and their assumptions matter. International Journal of Life Cycle Assessment. 2023. doi: 10.1007/s11367-023-02210-9. PubMed PMID: WOS:001168544000001.

201. Tuomisto HL, Hodge ID, Riordan P, Macdonald DW. Exploring a safe operating approach to weighting in life cycle impact assessment - a case study of organic, conventional and integrated farming systems. Journal of Cleaner Production. 2012;37:147-53. doi: 10.1016/j.jclepro.2012.06.025. PubMed PMID: WOS:000309375200015.

202. Change IPoC. Climate Change 2013. The Physical Science Basis. . Intergovernmental Panel on Climate Change, 2013.

203. Agriculture SBo. Statistical database. : Jordbruksverket; 2012 [cited 2024 07 Oct]. Available from: http://www.jordbruksverket.se/etjanster/etjanster/ statistikdatabas.4.6a459c18120617aa58a80001011.html.

204. Befolkningsstatistik. Befolkningsstatistik. (Population statistics) Örebro, Sweden 2014. Available from: http://www.scb.se/sv_/ Hitta-statistik/Statistik-efter-amne/Befolkning/ Befolkningens-sammansattning/Befolkningsstatistik/.

205. Ministers NCo. Nordic Nutrition Recommendations 2012. Copenhagn: 2014.

206. Enghardt Barbieri H, Lindvall C. De svenska näringsrekommendationerna översatta till livsmedel : underlag till generella råd på livsmedels- och måltidsnivå för friska vuxna. (The Swedish nutrition recommendations translated into food-documentation for general advice on food and meal level for healthy adults). Uppsala, Sweden: Livsmedelsverket; 2003.

207. Streiner DL, Norman GR, Cairney J. Health Measurement Scales: A practical guide to their development and use. 5th Edition ed. Oxford Oxford University Press.; 2015.

208. Bland JM. Statistics Notes: Validating scales and indexes. BMJ. 2002;324(7337):606-7. doi: 10.1136/bmj.324.7337.606.

209. Bianchi M, Strid A, Winkvist A, Lindroos A-K, Sonesson U, Hallström E. Systematic Evaluation of Nutrition Indicators for Use within Food LCA Studies. Sustainability. 2020;12(21):8992. doi: 10.3390/su12218992.
